# Neoadjuvant immunotherapy in mismatch-repair-proficient colon cancers

**DOI:** 10.1038/s41586-025-09679-4

**Published:** 2025-10-20

**Authors:** Pedro B. Tan, Yara L. Verschoor, José G. van den Berg, Sara Balduzzi, Niels F. M. Kok, Marieke E. Ijsselsteijn, Kat Moore, Adham Jurdi, Antony Tin, Paulien Kaptein, Monique E. van Leerdam, John B. A. G. Haanen, Emile E. Voest, Noel F. C. C. de Miranda, Ton N. Schumacher, Lodewyk F. A. Wessels, Myriam Chalabi

**Affiliations:** 1 Department of Gastrointestinal Oncology, Netherlands Cancer Institute, Amsterdam, the Netherlands; 2 Department of Pathology, Netherlands Cancer Institute, Amsterdam, the Netherlands; 3 Department of Biometrics, Netherlands Cancer Institute, Amsterdam, the Netherlands; 4 Department of Surgery, Netherlands Cancer Institute, Amsterdam, the Netherlands; 5 Department of Pathology, Leiden University Medical Center, Leiden, the Netherlands; 6 Department of Molecular Carcinogenesis, Netherlands Cancer Institute, Amsterdam, the Netherlands; 7 Natera Inc, Austin, TX USA; 8 Department of Molecular Oncology and Immunology, Netherlands Cancer Institute, Amsterdam, the Netherlands; 9 Department of Gastroenterology and Hepatology, Leiden University Medical Center, Leiden, the Netherlands; 10 Department of Medical Oncology, Netherlands Cancer Institute, Amsterdam, the Netherlands; 11 Oncode Institute, Utrecht, the Netherlands; 12 Department of Hematology, Leiden University Medical Center, Leiden, the Netherlands

**Keywords:** Colon cancer, Cancer microenvironment, Cancer immunotherapy, Translational research, Predictive markers

## Abstract

Immune checkpoint blockade has led to paradigm shifts in the treatment of various tumour types^1–4^, yet limited efficacy has been observed in patients with metastatic mismatch-repair-proficient (pMMR) colorectal cancer^5^. Here we report clinical results and in-depth analysis of patients with early-stage pMMR colon cancer from the phase II NICHE study (ClinicalTrials.gov: NCT03026140). A total of 31 patients received neoadjuvant treatment of nivolumab plus ipilimumab followed by surgery. The response rate was 26% and included six patients with a major pathological response (10% or less residual viable tumour). One patient with an ongoing clinical complete response did not undergo surgery. Circulating tumour DNA was positive in 26 of 31 patients at baseline, and clearance was observed in 5 of 6 responders before surgery, whereas 19 of 20 non-responders remained circulating tumour DNA positive. Responses were observed despite a low tumour mutational burden in all tumours, whereas chromosomal genomic instability scores were significantly higher in responders than in non-responders. Furthermore, responding tumours had significantly higher baseline expression of proliferation signatures and TCF1, and imaging mass cytometry revealed a higher percentage of Ki-67^+^ cancer and Ki-67^+^CD8^+^ T cells in responders than in non-responders. These results provide a comprehensive analysis of response to neoadjuvant immune checkpoint blockade in early-stage pMMR colon cancers and identify potential biomarkers for patient selection.

## Abstract (web summary)

The results from the phase II NICHE trial on immunotherapy in mismatch-proficient colon cancer are presented.

## Main

Colorectal cancers (CRCs) can be divided into two biologically distinct subtypes based on the status of the DNA MMR system: pMMR and MMR-deficient (dMMR) tumours. Although immune checkpoint blockade (ICB) has become standard of care in patients with metastatic dMMR tumours^1,6^, metastatic pMMR tumours remain largely unresponsive to ICB^5^. This disparity has, at least in part, been attributed to the substantially lower tumour mutational burden (TMB) seen in pMMR tumours.

In recent years, neoadjuvant ICB has shown high pathological response rates that were associated with improved survival in various solid tumour types^2–4,7,8^. Of note, in patients with non-metastatic dMMR CRC, neoadjuvant ICB induced remarkably high response rates without disease recurrences^9–13^. Recent studies have also suggested that neoadjuvant ICB induces responses in a subgroup of patients with non-metastatic pMMR CRC^14–16^. Underlying mechanisms probably contributing to this high efficacy in earlier disease stages include a higher abundance of tumour-infiltrating immune cells, improved T cell function and a lower level of local and systemic immune suppression^17–21^.

Considering the lack of efficacy of ICB in patients with metastatic pMMR CRC, and considering that pMMR tumours constitute 85% of non-metastatic colon cancers^22^, strategies aimed at the development of effective immunotherapy regimens for patients with non-metastatic pMMR CRC are of high interest. Preclinical data have showed that the inhibition of prostaglandin E_2_ synthesis, a product of the cyclooxygenase-2 (COX-2) pathway, suppresses tumour-promoting inflammation and may enhance immunotherapy response^23^.

Given these data, we sought to explore the effects of neoadjuvant nivolumab plus ipilimumab, with or without selective COX-2 inhibition using celecoxib, in patients with early-stage pMMR colon adenocarcinoma. Here we report clinical outcomes as well as in-depth translational analyses from patients treated in the pMMR cohort of the phase II NICHE platform trial (NCT03026140).

## Patient and treatment characteristics

**Extended Data Fig. 1:**
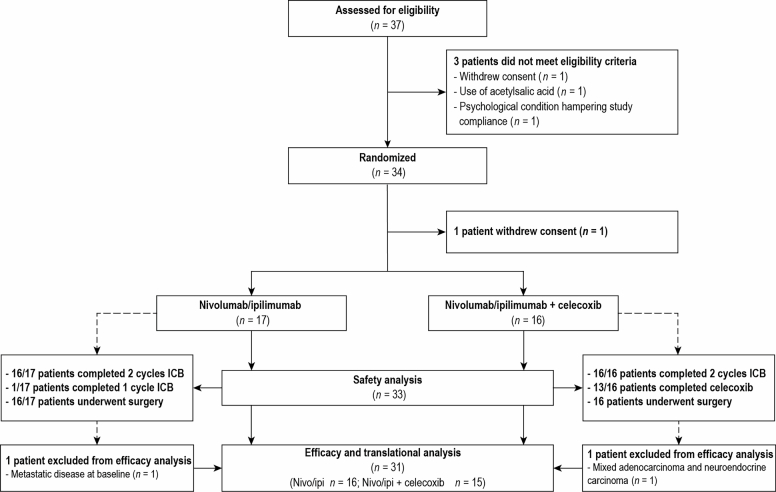
Consort Flow Diagram. Flowchart of patients screened and randomized in the NICHE study.

From 29 June 2017 until 26 July 2021, 37 patients were screened for eligibility, of whom 33 were randomized and started treatment with either nivolumab and ipilimumab alone (*n* = 17) or combined with celecoxib (*n* = 16). The relatively slow accrual rate for this cohort, despite the high incidence of pMMR colon cancers, is probably attributable to the generally assumed unresponsiveness of pMMR colon cancers to immunotherapy, combined with the COVID-19 pandemic. The median age was 62 years (range 44–77 years) and 45% of patients were female. Among 33 patients, 18 patients (55%) had lymph node-positive disease as assessed on baseline computed tomography (CT) scan (Table 1). Two patients, one from each treatment group, who retrospectively did not meet inclusion criteria, were excluded from efficacy and translational analyses. One patient was found to have a mixed adenocarcinoma and neuroendocrine carcinoma in the resection specimen and the other had metastatic disease at preoperative imaging, which was retrospectively visible on baseline imaging. Both patients had completed two cycles of ICB according to protocol and were included in the safety analyses (Extended Data Fig. 1).

**Table 1:** Baseline patient and tumour characteristics

Characteristic	Overall (*n* = 33)
**Age (years), median (range)**	62 (44–77)
**Sex (*n*** **(%))**	
Female	15 (45%)
Male	18 (55%)
**WHO performance status (*n*** **(%))**	
0	31 (94%)
1	2 (6%)
**Clinical disease stage (*n*** **(%))**	
I	4 (12%)
II	11 (33%)
III	18 (55%)
**Primary tumour location (*n*** **(%))**	
Right	9 (27%)
Left	24 (73%)

Baseline characteristics for all patients who received at least one cycle of study treatment.

**Fig. 1:**
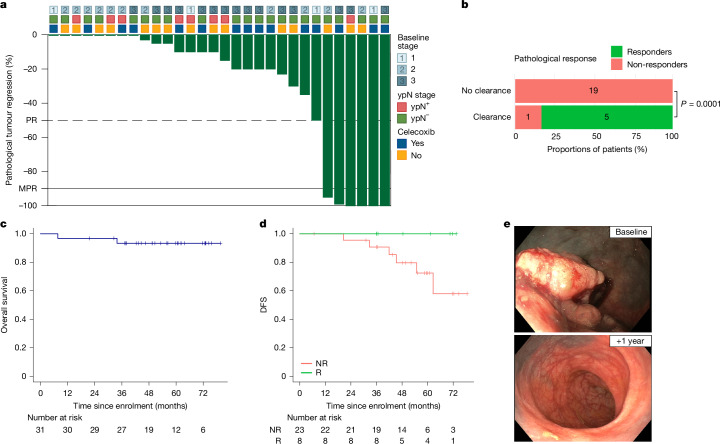
Pathological responses and survival outcomes after short-course neoadjuvant ICB. **a**, Percentage of pathological regression shown per tumour. The dashed line demarcates pathological response (PR) at 50% regression, and the black horizontal line depicts the demarcation for MPR at 90% regression. The clinical stage at baseline (upper boxes), the post-treatment pathological lymph node status (middle boxes) and whether patients received celecoxib (lower boxes) are also shown above the graph. **b**, Association between pathological response and clearance of ctDNA pre-surgery, either after one or two cycles of treatment (*n* = 25). One responder who was ctDNA^+^ after cycle 1 and for whom no pre-surgical data were available was excluded from this analysis. Significance was tested using a one-sided Fisher’s exact test. **c**, Kaplan–Meier plot for overall survival in patients included in the efficacy analysis. **d**, Kaplan–Meier plot for DFS for pathological responders (R; green) versus non-responders (NR; red) included in the efficacy analysis. **e**, Endoscopic images of the patient undergoing non-operative management with active surveillance, showing the tumour at baseline (top) and a clinical complete response at 1 year after treatment (bottom).

Treatment with short-course neoadjuvant ICB was generally well tolerated (Extended Data Table 1) and extensive safety data have been previously reported^9,24^. Both cycles of ICB were administered in 32 of 33 patients (97%). Celecoxib was discontinued in 3 of 16 patients randomized to that arm (19%) after 15, 21 and 24 days due to toxicity that was deemed at least possibly related to this treatment, including dyspepsia, dyspnoea and rash. Surgery was performed in 32 of 33 patients (97%) with a median interval of 4.6 weeks (range 3.7–6.0 weeks) between the first cycle of immunotherapy and resection, all without delays and with tumour-free margins. The remaining patient had a delay in surgery due to grade 3 myositis and required long-term immunosuppressive treatment before symptoms resolved. During this time, the patient underwent endoscopies and CT scans every 3 months, and by the time surgery could be performed safely, there was evidence of an ongoing clinical complete response and the patient waived surgery. The patient is under active surveillance without signs for local or distant recurrence, with a current follow-up of 42 months after treatment (Fig. 1e).

## Clinical outcomes and ctDNA

Out of the 31 patients included in the efficacy analyses, 30 patients underwent surgery. Response to treatment was observed in 8 of 31 patients (26%, 95% confidence interval (CI) 12–45%; Fig. 1a), which included one patient with a clinical complete response who did not undergo surgery. Among the 30 patients who underwent surgery, 7 patients (23%) had a pathological response, defined as 50% or less residual viable tumour (RVT). Six of these 7 patients (6 of 30; 20%) had a major pathological response (MPR; defined as 10% or less RVT), of which 3 (10%) were pathological complete responses (pCR), defined as no RVT in both the primary tumour and the lymph nodes (see Extended Data Table 2 for responses in individual patients, including American Joint Committee on Cancer (AJCC) stage and RVT). Responses were observed across stages as assessed at baseline, with 4 out of 8 (50%) responders having stage 3, 2 (25%) having stage 2 and 2 (25%) having stage 1 tumours, indicating that clinical high-risk pMMR tumours are also amenable to response to neoadjuvant ICB. Response rates were comparable in patients treated with or without celecoxib (4 of 15 (27%) and 4 of 16 (25%), respectively).

Eight patients had tumour-positive lymph nodes in their resection specimen and seven of these patients, all non-responders, received adjuvant chemotherapy. The remaining patient, a responder with a pCR in the primary tumour and partial response in lymph nodes, declined adjuvant chemotherapy after counselling. One patient received adjuvant chemotherapy based on baseline clinical staging and local pathological staging of an ypT4b tumour macroscopically invading the abdominal wall, whereas central histopathological assessment revealed an ypT3 tumour.

**Extended Data Fig. 2:**
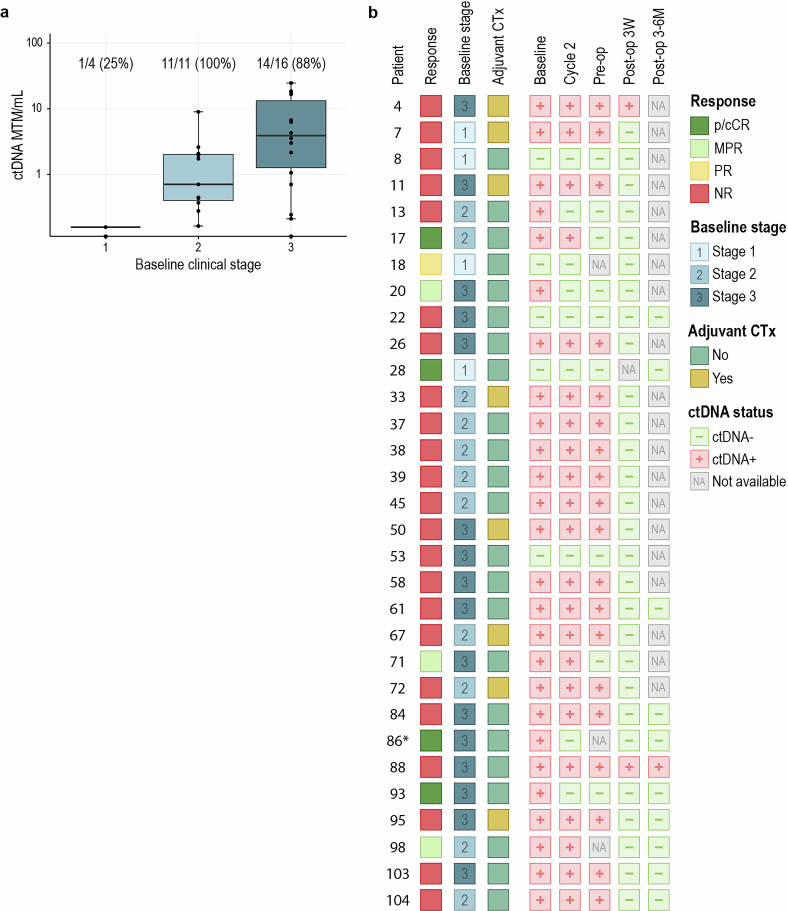
Overview of ctDNA dynamics per patient. **a**, Baseline ctDNA concentration according to baseline clinical stage. The number and percentage of patients with positive ctDNA is indicated above each box. Boxplots represent the median and interquartile range, whiskers extend from the hinge to the largest value within 1.5 x interquartile range from the hinge. **b**, For each patient, the colored boxed indicate the post-treatment response (p/cCR: pathological/clinical complete response; MPR: major pathological response; PR: partial response; NR: non-response), baseline stage based on CT scan, whether adjuvant chemotherapy (CTx) was given along with the ctDNA status at baseline, prior to cycle 2, pre-operatively (pre-op), 3 weeks post-operatively (post-op 3 W) and 3 to 6 months post-operatively (post-op 3–6 M). Due to patient 86 not undergoing surgery, no sample was available for the pre-operative time point and samples obtained at 3 and 6 months after the first cycle of immunotherapy were used as post-operative time points.

Circulating tumour DNA (ctDNA) was detected in 26 of 31 patients (84%) at baseline, including 6 responders and 20 non-responders. ctDNA concentrations were associated with baseline clinical staging, with the highest concentrations observed in patients with stage 3 disease (Extended Data Fig. 2a). Analysis of ctDNA dynamics in patients who were ctDNA^+^ at baseline showed clearance of ctDNA before surgery either after the first (*n* = 3) or second (*n* = 2) cycle in 5 out of 6 responders. One responder remained ctDNA^+^ after the first cycle and no pre-surgical data were available to assess clearance after the second cycle. By contrast, 19 of 20 (95%) non-responders remained ctDNA^+^ at both time points before surgery (Fig. 1b and Extended Data Fig. 2b). Two patients, both non-responders, remained ctDNA^+^ at 3 weeks post-surgery.

With a median follow-up of 56 months (range 8–80 months), 25 out of 31 patients in the efficacy analysis (81%) were alive and disease-free, whereas 6 patients (19%) experienced disease recurrence, of which 1 patient died due to disease progression. Median overall survival and disease-free survival (DFS) were not reached (Fig. 1c). Among all patients in the efficacy analysis, the 3-year DFS was 93% (95% CI 85–100%). The two patients excluded from the efficacy analysis both received chemotherapy, and one patient also underwent local treatment for liver metastases. Both patients were disease free and alive at the time of data cut-off, with a follow-up of 61 and 69 months, respectively.

Of note, all responders remained disease free, whereas all six patients with disease recurrence were pathological non-responders and all were still ctDNA^+^ before surgery (Fig. 1d). Of the two patients that remained ctDNA^+^ post-surgery, one patient had disease recurrence, whereas the other patient, who had received adjuvant chemotherapy due to tumour-positive lymph nodes, remained disease free.

## Response-associated genomic features

**Fig. 2:**
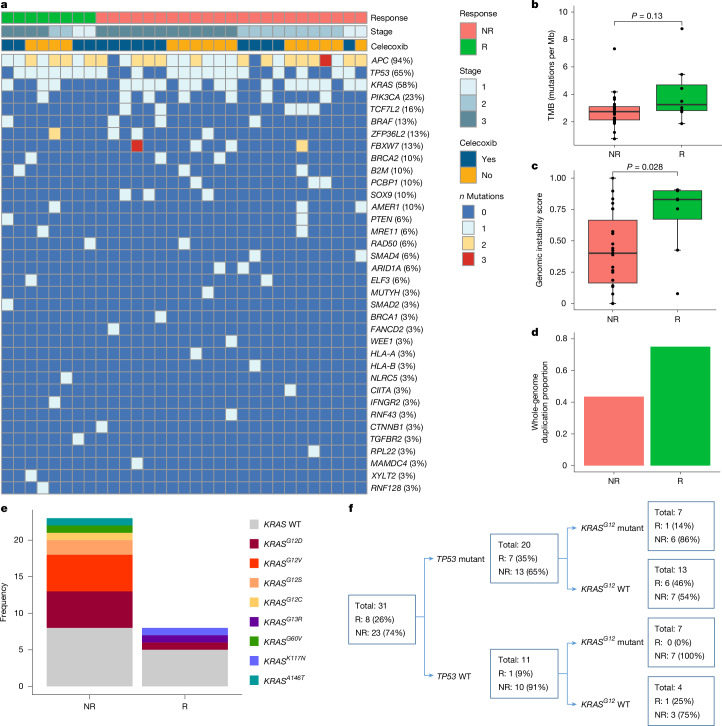
Genomic features of pMMR responders and non-responders. **a**, Mutational landscape of pMMR tumours (*n* = 31), filtered for pathogenicity or likely functional impact (see Methods). The heatmap is coloured by the number of mutations detected in each gene and ordered by mutation frequency. Each column represents a patient, with baseline clinical staging, celecoxib treatment and response annotations presented above. **b**,**c**, Comparison of TMB (**b**) and ASCAT genomic instability scores (**c**) between responders (*n* = 8) and non-responders (*n* = 23). The boxplots represent the median and interquartile range, and whiskers extend from the hinge to the largest value within 1.5× the interquartile range from the hinge. Distributions were compared with a two-sided Wilcoxon rank-sum test. **d**, Proportion of tumours with whole-genome duplication in response groups. **e**, Frequency of *KRAS* mutations in responders (*n* = 8) and non-responders (*n* = 23). **f**, Combination of *TP53* and *KRAS* mutational status and associations with response. *KRAS*^*G12*^ WT groups include *KRAS* WT (*n* = 13) and other *KRAS* mutants (*n* = 4).

**Extended Data Fig. 3:**
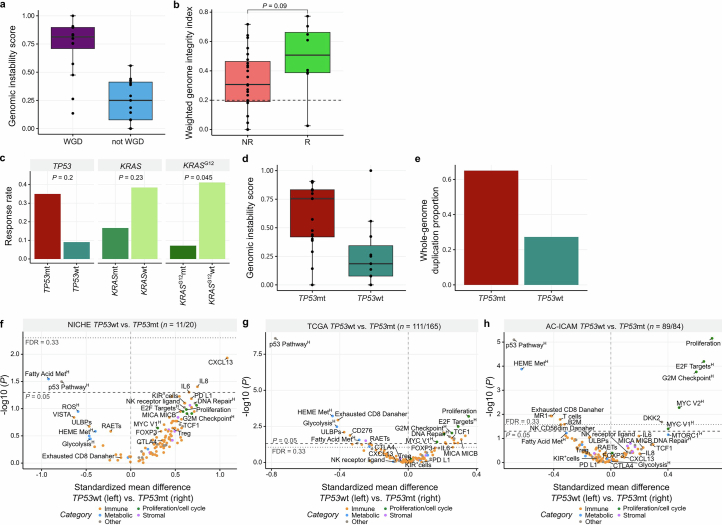
Genomic and transcriptomic features of *TP53*mt tumours. **a**, Comparison of ASCAT genomic instability scores between tumours with whole-genome duplication (WGD) (*n* = 16) and without (*n* = 15). **b**, Comparison of weighted genome integrity index between responders (*n* = 8) and non-responders (*n* = 23). Dashed horizontal line indicates a cut-off of 0.2 as a surrogate measure for chromosomal instability. Distributions were compared with a two-sided Wilcoxon rank-sum test. **c**, Response rates across *TP53*, *KRAS* and *KRAS*^G12^ mutated groups. Significance was tested using a two-sided Fisher’s exact test. **d**, Comparison of genomic instability scores between *TP53*mt (*n* = 20) and *TP53*wt tumours (*n* = 11). **e**, Proportion of tumours with whole-genome duplication in *TP53* groups. **f**, Volcano plot comparing expression of gene sets for *TP53*mt (*n* = 20) and *TP53*wt (*n* = 11) NICHE tumours pre-treatment. **g**,**h**, Volcano plot comparing expression of gene sets for *TP53*mt (*n* = 165) and *TP53*wt (*n* = 111) tumours in TCGA (**g**) and *TP53*mt (*n* = 84) and *TP53*wt (*n* = 89) tumours in AC-ICAM^29^ (**h**). **a**,**b**,**d**, Boxplots represent the median and interquartile range, whiskers extend from the hinge to the largest value within 1.5 x interquartile range from the hinge. **f-h**, Gene sets are coloured based on distinct TME categories. Distributions were compared with two-sided Wilcoxon rank-sum tests. Adjustment for multiple comparisons was performed to obtain the FDR. The dashed horizontal line indicates a *P* value of 0.05 and the dotted line indicates an FDR of 0.33. The dashed vertical line indicates null effects.

Whole-exome sequencing was performed to characterize genomic features of pMMR tumours and to identify potential biomarkers of response to ICB in an exploratory analysis. All patients had a low TMB (range 0.8–8.8 mutations per Mb), and TMB was not significantly higher in responders than in non-responders (median 3.3 versus 2.7 mutations per Mb, *P* = 0.13; Fig. 2b). In addition to mutational burden, tumour immunity may be affected by the landscape of chromosomal and copy number alterations, which reflects the level of genomic instability. Responders presented significantly higher chromosomal genomic instability scores than non-responders (median 0.83 versus 0.40; *P* = 0.028; Fig. 2c), which was furthermore associated with more frequent whole-genome duplication events (75% versus 43%; Fig. 2d and Extended Data Fig. 3a). Using a weighted genome instability index cut-off of 0.2 as a surrogate metric for chromosomal instability (CIN^+^)^25,26^, 23 of 31 (74%) tumours were labelled as CIN^+^. Of note, the median weighted genome instability index score was higher in responders than in non-responders, with 7 out of 8 tumours classified as CIN^+^ (Extended Data Fig. 3b).

Analysis of known CRC driver mutations revealed frequent mutations in *APC* (94%), *TP53* (65%) and *KRAS* (58%; Fig. 2a), all determined to have a functional impact (Supplementary Table 1). All 20 *TP53* mutations had OncoKB loss of function or ClinVar pathogenic annotations. Most responders had mutations in *TP53* (7 of 8, 88%), which were detected at a lower frequency in non-responders (13 of 23, 57%; Fig. 2f). Correspondingly, *TP53*-mutant tumours had a numerically higher but not statistically significant response rate than *TP53* wild-type (WT) tumours (7 of 20, 35% versus 1 of 11, 9%, respectively; *P* = 0.20; Extended Data Fig. 3c). These data are in line with the overall higher rate of genomic instability and the proportion of whole-genome duplications in responders, both frequently observed in *TP53*-mutant tumours^27^ (Extended Data Fig. 3d,e).

Among the 18 tumours with pathogenic *KRAS* mutations, *KRAS*^*G12*^ substitutions were most frequent (*n* = 14, 78%) and included 6 *KRAS*^*G12D*^, 5 *KRAS*^*G12V*^, 2 *KRAS*^*G12S*^ and 1 *KRAS*^*G12C*^ substitutions. In four patients, other *KRAS* mutations were observed and included *KRAS*^*G13R*^, *KRAS*^*G60V*^, *KRAS*^*A146T*^ and *KRAS*^*K117N*^. All 18 *KRAS* mutations were gain of function. When considering all *KRAS* mutations, a non-significant trend towards increased responses was observed in patients with *KRAS* WT tumours (5 of 13, 38%) compared with those with *KRAS*-mutant tumours (3 of 18, 17%; *P* = 0.23). When specifically considering *KRAS*^*G12*^, a response was observed in 1 of 14 (7%) tumours with a *KRAS*^*G12*^ mutation, whereas 7 of 17 (41%; *P* = 0.045) *KRAS*^*G12*^ WT tumours responded (Fig. 2e). Together, these data suggest a possible enrichment for response in *TP53*-mutant; *KRAS*^*G12*^ WT tumours (6 of 13; 46%; Fig. 2f) compared with limited responses in *TP53*-mutant;*KRAS*^*G12*^-mutant tumours (1 of 7, 14%) and *TP53* WT;*KRAS*^*G12*^-mutant tumours (0 of 7, 0%).

## Baseline proliferation and response

**Fig. 3:**
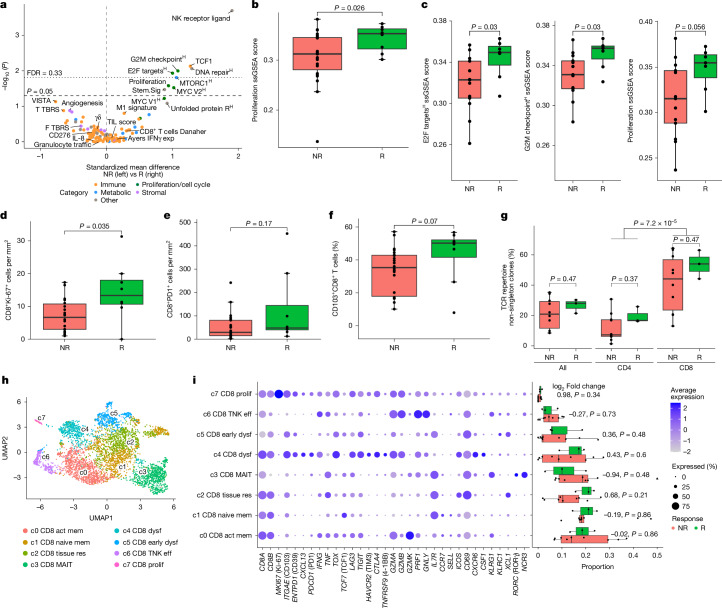
Baseline gene signatures, proliferation and T cell populations in responders and non-responders. **a**, Volcano plot comparing gene set expression for responders (*n* = 8) and non-responders (*n* = 23). Gene sets are coloured by TME categories. The dashed horizontal line indicates *P* = 0.05, the dotted line denotes false discovery rate (FDR) = 0.33 and the dashed vertical line shows null effects. exp, expanded; F TBRS, fibroblast TGFβ response signature; NK, natural killer; Stem.Sig, stemness signature; T TBRS, T cell TBRS; TIL, tumour-infiltrating lymphocyte; VISTA, V-domain Ig suppressor of T cell activation. Superscript H indicates hallmark signatures. **b**, Proliferation single sample gene set enrichment analysis (ssGSEA) scores between responders (*n* = 8) and non-responders (*n* = 23). **c**, E2F targets, G2M checkpoint and proliferation signature in *TP53*-mutant responders (*n* = 7) and non-responders (*n* = 13). **d**, Density of Ki-67^+^CD8^+^ in IMC samples for responders (*n* = 8) and non-responders (*n* = 22). **e**, Density of CD8^+^PD1^+^ T cells in immunohistochemistry for responders (*n* = 8) and non-responders (*n* = 21). **f**, Percentage of CD103^+^ in CD8^+^ cells in IMC biopsies for responders (*n* = 8) and non-responders (*n* = 22). **g**, Percentage of non-singleton scRNA-seq TCR repertoires for all, CD8^+^ and CD4^+^ T cells in responders (*n* = 3) and non-responders (*n* = 10). Comparisons include response groups and CD8^+^ versus CD4^+^ for all patients (*n* = 13). **h**, Uniform manifold approximation and projection (UMAP) of CD8^+^ T cell clusters (*n* = 4,588). **i**, Dotplot of CD8^+^ T cell clusters and within-sample cluster distributions between responders (*n* = 3) and non-responders (*n* = 9), indicating log_2_ fold change between mean cluster proportions. One non-responder was excluded due to low CD8^+^ T cells. The triangle denotes a non-responder with a 0.71 proportion of c3 CD8^+^ mucosal-associated invariant T (MAIT) cells. Full cluster names: c0 activated memory, c1 naive memory, c2 tissue-resident, c3 mucosal-associated invariant, c4 exhausted/dysfunctional, c5 early exhausted/dysfunctional, c6 CD8^+^ T natural killer-like (TNK) effector and c7 proliferating. The boxplots represent the median and interquartile range, and whiskers extend from the hinge to the largest value within 1.5× the interquartile range from the hinge (**b**–**g**,**i**). Distributions were compared with two-sided Wilcoxon rank-sum tests (**a**–**g**,**i**).

**Extended Data Fig. 4:**
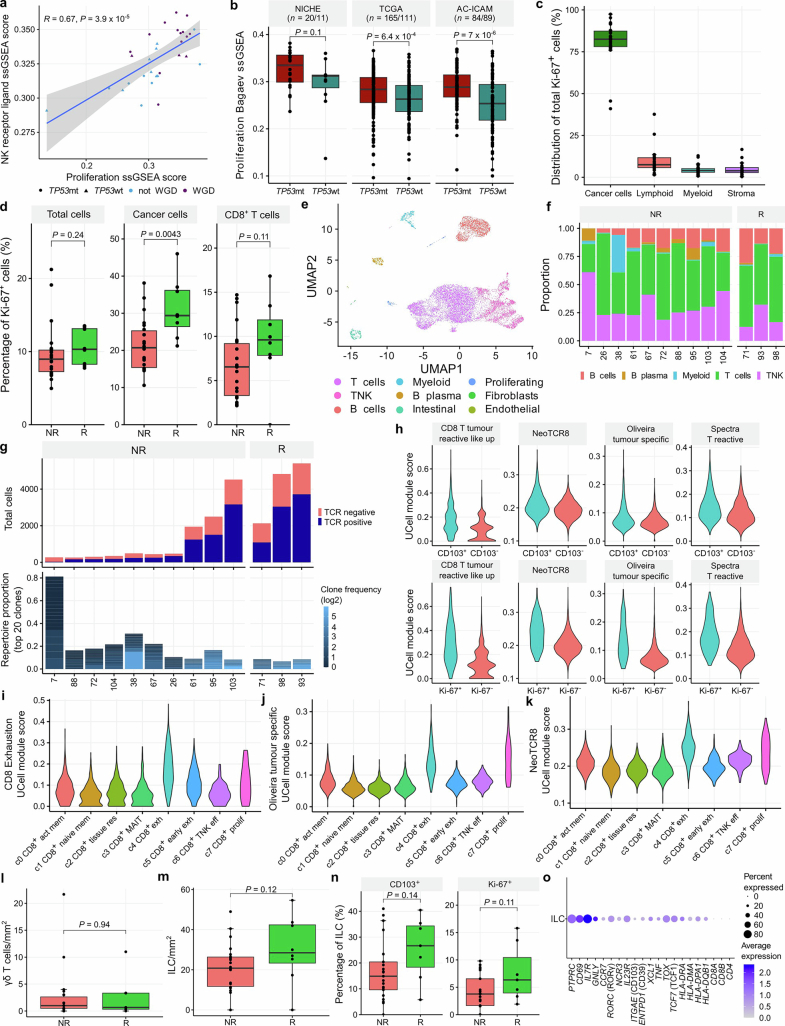
Pre-treatment associations of proliferation, NK receptor ligands, tumour-infiltrating lymphocyte phenotypes and response. **a**, Correlation between NK receptor ligand and proliferation signature (*n* = 31). Data points are coloured and shaped by *TP53* and WGD status. The grey area indicates the 95% confidence interval of the blue regression line. Pearson’s r values and fits are shown. **b**, Proliferation in *TP53*mt vs. *TP53*wt tumours in NICHE, AC-ICAM and TCGA cohorts. **c**, Percentage of total Ki-67^+^ cells across cell categories in IMC (*n* = 30) **d**, Percentage of Ki-67^+^ cells in total, cancer and CD8^+^ cells in IMC for responders (*n* = 8) and non-responders (*n* = 22). **e**, scRNAseq UMAP (*n* = 23947), coloured by broad celltypes. **f**, Quantification of immune proportions for responders (*n* = 3) and non-responders (*n* = 10). The TNK cluster contains CD8^+^, NK and innate lymphoid cells. **g**, Total cells coloured by TCR detection, and proportion of TCR repertoires occupied by the top 20 clones, coloured by log2 frequencies for responders (*n* = 3) and non-responders (*n* = 10). **h**, Enrichment of scRNAseq tumour-reactivity signatures in CD103^+^ and Ki-67^+^ CD8^+^ T cells, **i-k**, Distribution of (**i**) CD8 Exhaustion, (**j**) Oliveira tumour specific and (**k**) NeoTCR8 signatures in CD8^+^ T cell clusters. **l-m** Comparison of γδ T (**l**) and innate lymphoid cell (ILC) density (**m**) in IMC for responders (*n* = 8) and non-responders (*n* = 22). **n**, Percentage of ILC positive for CD103 and Ki-67 in IMC for responders (*n* = 7) and non-responders (*n* = 21). **o**, Dotplot of ILC marker expression pooling all cells (*n* = 80).**b**,**d**, **l-n** Distributions were compared with two-sided Wilcoxon’s rank sum tests. Boxplots represent the median and interquartile range,whiskers extend from the hinge to the largest value within 1.5 x interquartile range from the hinge.

To characterize molecular features of the tumour microenvironment (TME), a comprehensive set of gene expression signatures was investigated (Supplementary Table 2). Transcriptomic analysis of pre-treatment samples showed that immune-related signatures, including those associated with IFNγ, T cell infiltration and activity, were not predictive of response (Fig. 3a). However, signatures related to cell proliferation and cell cycle were enriched in responders, including hallmarks for G2M checkpoint (*P* = 0.004), E2F (*P* = 0.012) and MYC targets (*P* = 0.03), as well as a proliferation signature (*P* = 0.026; Fig. 3b). Of note, the most pronounced association with response was a signature of natural killer cell receptor ligands (*P* = 2 × 10^−4^), which was in turn correlated with the proliferation signature (*P* = 3.9 × 10^−5^; Extended Data Fig. 4a). In addition, expression of TCF1 (encoded by *TCF7*), a marker of naive or stem-like T cells with proliferative potential^28^, was significantly higher in responders than in non-responders (*P* = 0.0074). Furthermore, an enrichment of the hallmark signature for DNA repair was observed in responders (*P* = 0.0087), consistent with the increased genomic instability in these tumours, as described above.

To explore TME features of untreated *TP53*-mutant versus *TP53* WT pMMR colon cancer, we analysed transcriptomic and genomic data from The Cancer Genome Atlas (TCGA) and atlas and compass of immune–colon cancer–microbiome interactions (AC-ICAM)^29^ cohorts (Extended Data Table 3). This revealed higher expression of proliferation and cell cycle signatures in *TP53*-mutant tumours (Extended Data Figs. 3f–h and 4b). In our study, even within tumours harbouring *TP53* mutations, scores for these signatures were still higher in responders than in non-responders, suggesting that proliferation may be an independent predictive biomarker (Fig. 3c).

To further investigate the cellular origin of the proliferation signal, Ki-67^+^ immunodetection alongside several cellular markers was performed by imaging mass cytometry (IMC). The majority of Ki-67^+^ cells corresponded to cancer cells (Extended Data Fig. 4c) and, in line with the transcriptomic data, a significantly higher percentage of Ki-67^+^ cancer cells was observed in responders than in non-responders (*P* = 0.004; Extended Data Fig. 4d). Although cancer cell proliferation was predominant, the density of Ki-67^+^CD8^+^ T cells was also significantly higher in responders than in non-responders (*P* = 0.035; Fig. 3d), an observation that is of particular relevance in view of the previously described association between intratumoural T cell proliferation and tumour reactivity^30^. Of note, the previously reported finding of a higher baseline CD8^+^PD1^+^ infiltration in responders^24^ was numerically but not statistically significantly higher in this complete cohort and thus previous results could not be confirmed (*P* = 0.17; Fig. 3e). However, a higher percentage of CD103 positivity, a tissue-residency marker consistent with tumour-reactive populations in CD8^+^ T cells^31^, was associated with response, albeit not significant (*P* = 0.07; Fig. 3f). To further dissect T cell phenotypes, single-cell RNA sequencing (scRNA-seq) and TCR-seq were performed on CD45^+^ cells in pre-treatment biopsies, which were available for 3 responders and 10 non-responders. The majority of immune cells was represented by T and T/natural killer (TNK) clusters (Extended Data Fig. 4e,f). Most TCR clones corresponded to singletons, with CD8^+^ T cells generally showing significantly greater clonal expansion than CD4^+^ T cells (*P* = 7.2 × 10^−5^). When comparing CD4^+^ and CD8^+^ TCR clonality between responders and non-responders, no significant differences were observed (*P* = 0.37 and *P* = 0.47; Fig. 3g and Extended Data Fig. 4g). CD8^+^ T cells displayed distinct transcriptomic states that translated to eight clusters, annotated with a comprehensive combination of markers and scRNA-seq signatures (Fig. 3h and Supplementary Table 2). CD8 cluster c4, a tissue-resident population with the highest CD103 expression (encoded by *ITGAE*), displayed transcriptomic markers of dysfunctional/exhausted cells, such as *ENTDP1*, *PDCD1*, *TOX* and *CXCL13*, as well as checkpoints such as *TIGIT*, *LAG3* and *CTLA4* (Fig. 3i and Extended Data Fig. 4i). Cluster c4 shared many of these transcriptomic features with the small proliferating cluster c7. Furthermore, most TCR clones from the proliferating compartment were shared with those observed in the c4 exhausted cell compartment (Fig. 3g,i). Consistently, both CD8^+^CD103^+^ and CD8^+^Ki-67^+^ T cells, as well as clusters c4 and c7, were enriched for tumour-reactivity signatures (Extended Data Fig. 4h,j,k). Although differences in CD8^+^ T cell cluster proportions were subtle between responders and non-responders profiled with scRNA-seq, these data support observations in IMC, by showing enrichment for tumour-reactive cell states in the CD103^+^ and Ki-67^+^CD8^+^ T cell pools that were associated with response in the larger IMC dataset.

To investigate innate-like immune cells relevant for the colon mucosa, γδ T cells and innate lymphoid cell (ILC) counts were retrieved from the IMC data. γδ T cells were present at low frequencies and did not differ at baseline (*P* = 0.94; Extended Data Fig. 4l). A trend towards higher density of ILC was observed in responders (*P* = 0.12), with higher CD103 and Ki-67 expression in these cells, similar to observations in CD8^+^ T cells (Extended Data Fig. 4m,n). Although representing the smallest TCR-negative cluster, ILC could be identified in scRNA-seq data (Extended Data Fig. 4o) as ILC3, positive for the transcription factor RORγt (encoded by *RORC*). Furthermore, the cluster had detectable expression of MHC class II, indicating an ability for antigen presentation.

## Immune activation regardless of response

**Fig. 4:**
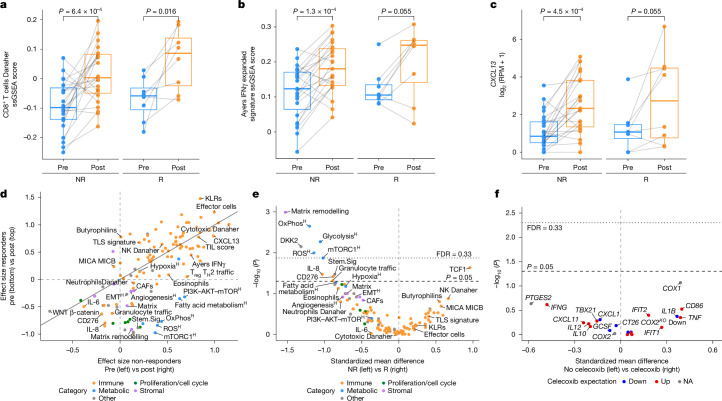
Immune mobilization after ICB in responders and non-responders. **a**–**c**, Comparison of paired pre-treatment and post-treatment expression for CD8^+^ T cells Danaher (**a**), Ayers IFNγ expanded (**b**) and *CXCL13* (**c**) in responders (*n* = 8) and non-responders (*n* = 23). The boxplots represent the median and interquartile range, and the whiskers extend from the hinge to the largest value within 1.5× the interquartile range from the hinge. Distributions were compared with a two-sided Wilcoxon signed-rank test. RPM, reads per million. **d**, Comparison of paired treatment effect sizes between response groups. The solid diagonal line indicates a correlation of 1, and the dashed lines indicate null effects. Gene sets are coloured by TME categories. EMT, epithelial-to-mesenchymal transition; KLR, killer cell lectin-like receptor; MICA, MHC class I polypeptide-related sequence A; OxPhos, oxidative phosphorylation; ROS, reactive oxygen species; T_H_2, T helper 2; T_reg_, regulatory T; TLS, tertiary lymphoid structure. **e**, Volcano plot comparing post-treatment expression of gene sets for responders (*n* = 8) and non-responders (*n* = 23). Gene sets are coloured by TME categories. Superscript H indicates hallmark signatures in **d**,**e**. **f**, Comparisons of post-treatment gene set expression between patients treated with celecoxib (*n* = 15) or without (*n* = 16). Gene sets are coloured based on expectation of COX-2 inhibition or knockout from literature. NA, not available. Distributions were compared with two-sided Wilcoxon rank-sum tests (**e**,**f**). Adjustment for multiple comparisons was performed to obtain the FDR. The dashed horizontal line indicates *P* = 0.05, the dotted line denotes FDR = 0.33 and the dashed vertical line indicates null effects (**e**,**f**).

**Extended Data Fig. 5:**
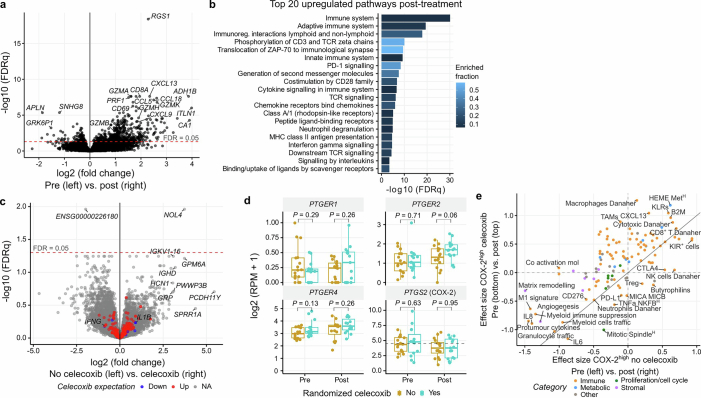
Differential expression of ICB and celecoxib treatment. **a**, Paired differential gene expression of matched pre vs. post-treatment samples. Dashed horizontal line indicates an FDR of 0.05 (*n* = 31). **b**, Top 20 Reactome pathways enriched in differentially upregulated genes after treatment, based on log2(fold change) > 0 and FDR < 0.05. Bars are coloured by the fraction of the gene set that was differentially upregulated. **c**, Differential gene expression of post-treatment samples from patients treated with celecoxib (*n* = 15) or without (*n* = 16). Dashed horizontal line indicates an FDR of 0.05. Genes are coloured based on expectation of COX-2 inhibition/knockout from literature^23^. **d**, Comparison of pre- and post-treatment expression of prostaglandin receptors and COX-2 in patients randomized to receive celecoxib (*n* = 15) or not (*n* = 16). Boxplots represent the median and interquartile range, whiskers extend from the hinge to the largest value within 1.5 x interquartile range from the hinge. Distributions were compared with two-sided Wilcoxon’s rank sum tests. Dashed line indicates the median pre-treatment COX-2 expression for high and low expression groups. **e**, Comparison of paired treatment effect sizes between patients with high baseline COX-2 expression treated with celecoxib (*n* = 9) or without (*n* = 6). Solid diagonal line indicates a correlation of 1, dashed lines indicate null effects. Gene sets are coloured based on distinct TME categories.

A paired transcriptomic analysis of matched pre-treatment and post-treatment samples was conducted to understand TME dynamics after ICB. This revealed clear immune mobilization in the majority of patients, as evidenced by differentially upregulated genes of interest that are associated with anticancer immunity and cytotoxicity such as *CXCL13* (*P* = 5.1 × 10^−5^), CD8 (*P* = 1.4 × 10^−5^) and IFNγ signatures (*P* = 9.2 × 10^−6^; Fig. 4a–c and Extended Data Fig. 5a,b). This immune activation was observed in both pathological responders as well as non-responders, and the expression of immune-related signatures increased with comparable magnitudes in both groups (Fig. 4d).

Despite the similar immune mobilization upon ICB, the dynamics of metabolic and stromal signatures differed between responders and non-responders. Specifically, mean scores for fatty acid metabolism and mTOR signalling increased in non-responders after treatment (*P* = 0.0067 and *P* = 0.048), indicative of metabolic reprogramming, whereas these signatures decreased in responders (*P* = 0.55 and *P* = 0.016; Fig. 4d). In addition, a significant decrease in the matrix remodelling signature was detected in responders (*P* = 0.016), potentially reflecting tumour cell clearance, whereas this signal remained relatively stable in non-responders (*P* = 0.8). When comparing post-treatment expression in responders versus non-responders, matrix remodelling (*P* = 0.001) and metabolic signatures such as oxidative phosphorylation (*P* = 0.0022) and glycolysis (*P* = 0.0054) were higher in non-responders (Fig. 4e), whereas TCF1 expression was higher in responders (*P* = 0.023).

Treatment with celecoxib was aimed at reducing prostaglandin E2 production^23^ and promoting antitumour immunity, yet when comparing patients treated with versus without celecoxib, immunomodulatory effects of celecoxib were not observed post-treatment (Fig. 4f and Extended Data Fig. 5c). In addition, when considering patients with high baseline COX-2 expression, no differences in post-treatment immunomodulation were observed in patients who were treated with or without celecoxib (Extended Data Fig. 5d,e).

**Fig. 5:**
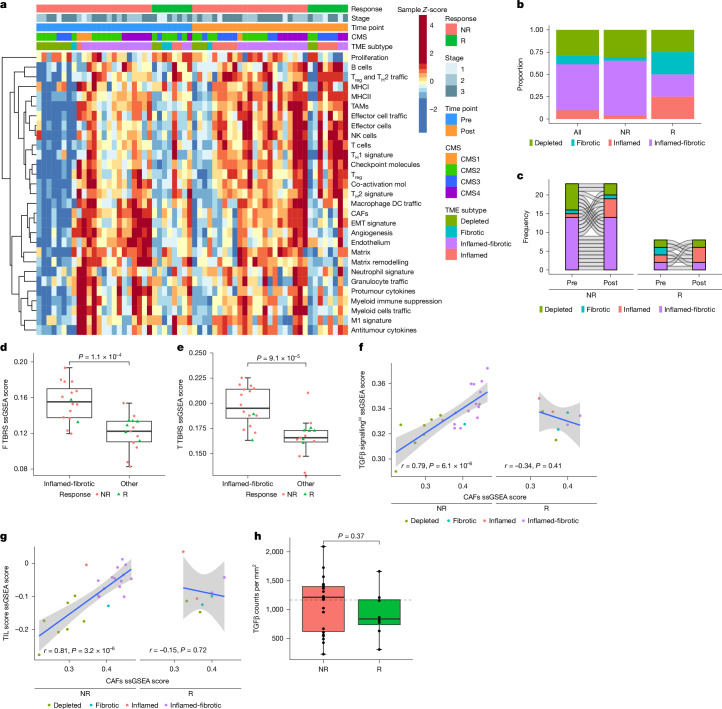
Association of TME subtypes and TGFβ signalling with response to ICB. **a**, Heatmap of TME signature scores^32^. Values are *Z*-scored and coloured by discrete quantiles. Each column represents a tumour sample (*n* = 62), with response, time point, baseline clinical staging, CMS^33^ and TME classifications annotated above. DC, dendritic cell; MHCI, MHC class I; TAM, tumour-associated macrophage. **b**, Proportion of TME subtypes in pre-treatment samples for all tumours (*n* = 31), responders (*n* = 8) and non-responders (*n* = 23). **c**, Alluvial plots indicating TME classification between pre-treatment and post-treatment samples for each patient in response groups. **d**,**e**, Comparison of pre-treatment ssGSEA scores for the F TBRS (**d**) and ssGSEA scores for the T TBRS (**e**) in inflamed-fibrotic (*n* = 16) and non-inflamed fibrotic (*n* = 15) tumours. The green triangles indicate responders, and the red dots denote non-responders. **f**,**g**, Correlation between pre-treatment ssGSEA scores for CAFs and TGFβ hallmark signatures (**f**), and for CAFs and TIL score signatures (**g**) for responders (*n* = 8) and non-responders (*n* = 23). The dots are coloured by TME classification. The grey area indicates the 95% CI of the blue regression line. Pearson’s *r* values and fits are shown. **h**, Comparison of total TGFβ counts per area in pre-treatment IMC samples for responders (*n* = 8) and non-responders (*n* = 22). The dashed line indicates the median TGFβ density. The boxplots represent the median and interquartile range, and the whiskers extend from the hinge to the largest value within 1.5× the interquartile range from the hinge. Distributions were compared with a two-sided Wilcoxon rank-sum test.

To evaluate interactions of stromal features and immune infiltration with response, the TME of each sample was classified into four distinct subtypes, that is, depleted, fibrotic, inflamed or inflamed-fibrotic, based on previously described signatures^32^, and tumours were additionally classified using the consensus molecular subtypes (CMSs)^33^ (Fig. 5a).

**Extended Data Fig. 6:**
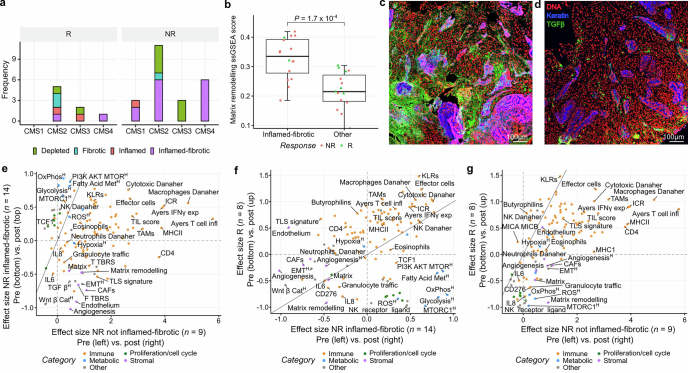
Association of inflamed-fibrotic state with TGFβ signalling and matrix remodelling. **a**, Frequency of consensus molecular subtypes (CMS) in pre-treatment samples for responders (*n* = 8) and non-responders (*n* = 23), coloured by TME subtypes. **b**, Comparison of pre-treatment ssGSEA scores for matrix remodelling in inflamed-fibrotic (*n* = 16) and non-inflamed fibrotic tumours (*n* = 15). The green triangles indicate responders and the red dots indicate non-responders. Distributions were compared with a two-sided Wilcoxon’s rank sum test. Boxplots represent the median and interquartile range, whiskers extend from the hinge to the largest value within 1.5 x interquartile range from the hinge. **c,d** Illustrative IMC regions of interest for (c) a TGFβ high non-responder and (d) a TGFβ low responder sample. 2-3 regions of interest were obtained per patient biopsy in one independent experiment. Nuclei (DNA) are coloured in red, Keratin in blue and TGFβ in green. Scale bar: 100 µM. **e-g** Comparison of paired treatment effect sizes between inflamed-fibrotic non-responders (*n* = 14) and non inflamed-fibrotic non-responders (*n* = 9) (**e**), responders (*n* = 8) and inflamed-fibrotic non-responders (*n* = 14) (**f**) and responders (*n* = 8) and non inflamed-fibrotic non-responders (*n* = 9) (**g**). Solid diagonal line indicates a correlation of 1, dashed lines indicate null effects. Gene sets are coloured based on distinct TME categories.

**Extended Data Fig. 7:**
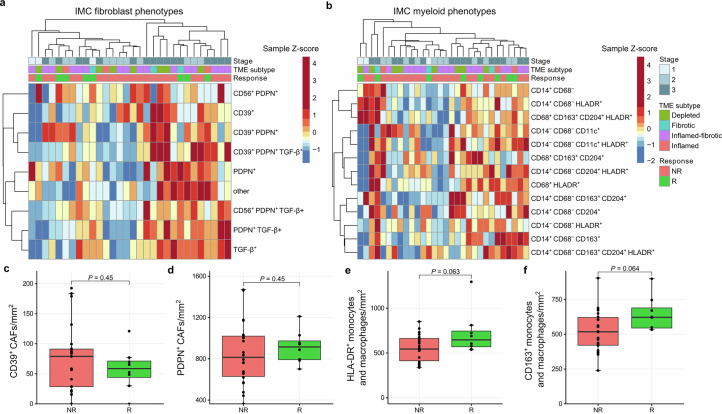
Cancer-associated fibroblast (CAFs) and myeloid subtypes. **a**,**b**, Heatmap of cancer-associated fibroblasts (**a**) and myeloid (**b**) phenotypes in IMC in pre-treatment biopsies for responders (*n* = 8) and non-responders (*n* = 22). Response, baseline clinical staging, and TME classifications are annotated above. **c-f** Comparison of density of CD39^+^ CAFs **(a)**, PDPN^+^ CAFs **(b)**, HLA-DR^+^ monocytes/macrophages **(c)** and CD163^+^ monocytes/macrophages (**d**) in IMC in pre-treatment biopsies for responders (*n* = 8) and non-responders (*n* = 22). **c-f** Distributions were compared with two-sided Wilcoxon’s rank-sum tests. Boxplots represent the median and interquartile range, whiskers extend from the hinge to the largest value within 1.5 x interquartile range from the hinge.

At baseline, there was evidence for two main TME subtypes: immune depleted and inflamed-fibrotic (Fig. 5b). Non-responding tumours were more often of the inflamed-fibrotic subtype (14 of 23, 61%) than responding tumours (2 of 8, 25%; Fig. 5c). Non-responders were more often of the CMS2 (11 of 23, 48%) and CMS4 (6 of 23, 26%) subtypes (Extended Data Fig. 6a), and each accounted for 6 of the 14 inflamed-fibrotic non-responding tumours. Inflamed-fibrotic tumours exhibited significantly higher expression of signatures related to fibroblast and T cell TGFβ signalling (*P* = 1.1 × 10^−4^ and *P* = 9.1 × 10^−5^; Fig. 5d,e), and this was in turn positively associated with signatures for cancer-associated fibroblasts (CAFs; Fig. 5f), immune infiltration (Fig. 5g) and matrix remodelling (Extended Data Fig. 6b). Furthermore, IMC analysis of pre-treatment biopsies revealed high TGFβ counts in a subgroup of non-responders (*P* = 0.37; Fig. 5h), with high levels of TGFβ in both cancer cells and CAFs, as well as extracellular matrix deposits (Extended Data Fig. 6c,d). On the basis of these findings, IMC markers were compared across CAF and myeloid populations to further profile these cell subsets. Although responders did not display an enrichment for distinct CAFs (Extended Data Fig. 7a,c,d), a trend for higher density of monocytes and macrophages positive for CD163 (*P* = 0.063) and HLA-DR (*P* = 0.064) was observed in responders (Extended Data Fig. 7b,e,f).

To further dissect differences within non-responders and the effects of ICB according to TME subtype, TME dynamics were investigated by comparing pre-treatment and post-treatment samples. Inflamed-fibrotic non-responding tumours still displayed enrichment of immune signatures post-treatment, accompanied by an increase in mTOR signalling and fatty acid metabolism (Extended Data Fig. 6e,f). Meanwhile, non-responders that were not inflamed-fibrotic at baseline showed stronger immune activation after ICB, along with increases in stromal signatures (Extended Data Fig. 6e,g). Together, these results suggest that TGFβ and stromal features correlate with expression of immune signatures in an important proportion of non-responders and may represent immune-suppressive mechanisms hampering response to ICB.

## Discussion

In this study, we have showed that neoadjuvant nivolumab plus ipilimumab exhibits promising antitumour activity in patients with pMMR colon cancer, a tumour type previously considered refractory to immunotherapy, with a response rate of 26% after only 4 weeks of treatment.

The current standard of care for patients with localized pMMR colon cancers consists of surgery, followed by adjuvant chemotherapy in case of stage 3 disease, which may also be considered for stage 2 disease with high-risk factors, including pT4 disease^34^. Adjuvant chemotherapy regimens, including fluorouracil with or without oxaliplatin, have been shown to improve DFS and overall survival. However, no advancements have been made since the introduction of oxaliplatin in 2004 to increase cure in this patient population^35^. While acknowledging the limitations of cross-trial comparisons and our small sample size, the pathological response rate of 26% following neoadjuvant immunotherapy in the current study is numerically similar to the 21–23% response rate to neoadjuvant chemotherapy in pMMR tumours in the OPTICAL and FOxTROT studies^36,37^. Although there is an ongoing interest in the potential of celecoxib either in combination with immunotherapy or as an addition to adjuvant chemotherapy^38^, the addition of celecoxib did not affect response rates or induce substantial immunomodulation in our study.

Neoadjuvant treatment regimens are increasingly being used in patients with colon cancer, and data from the FOxTROT study suggest a strong correlation between pathological response to neoadjuvant chemotherapy and lower risk of recurrence^36^. Over the past years, a growing number of studies has started to explore neoadjuvant immunotherapy in patients with both pMMR and dMMR colon cancer^11,12,14,16,39^. For neoadjuvant immunotherapy, the association between response and improved recurrence-free survival appears to be stronger than targeted therapies, as observed in melanoma^7,8^. In addition, support for a highly favourable survival in patients with colon cancer with a pathological response to neoadjuvant ICB is provided by the NICHE-2 study, in which 98% of patients with locally advanced dMMR colon cancer had a pathological response to neoadjuvant immunotherapy, and none of these patients had recurrent disease at a median follow up of 3 years^9,10^.

In the current study, a similar relationship between pathological response and a high DFS can be postulated, albeit with smaller numbers, with none of the eight responders experiencing disease recurrence compared with 6 of 23 non-responders having recurrence of disease at a median follow-up of 48 months. It should be noted that 48% of patients in this efficacy analysis had tumours that were classified as stage 1 or stage 2 at baseline, which have been linked to lower 5-year recurrence rates of around 5% and 12%, respectively^40^. However, responses in our study were not limited to patients with stage 1 or stage 2 disease and were also observed in patients with stage 3 disease with a recurrence risk that has previously been described at approximately 33%^40^. An important limitation of neoadjuvant treatment for colon cancer remains the limited ability to accurately predict lymph node metastases on CT scans, which may lead to overstaging and potential overtreatment when focusing on lymph node staging^41,42^; however, we do note that in this study, clinical staging was associated with the detection of ctDNA at baseline. We also show that pre-surgical clearance of ctDNA is strongly correlated with pathological response after neoadjuvant immunotherapy, as evidenced by ctDNA clearance in responders, whereas all but one non-responder remained ctDNA^+^. These data are in line with results from NICHE-2 in locally advanced dMMR tumours showing clearance of ctDNA in the majority of patients with an MPR or pCR following neoadjuvant nivolumab plus ipilimumab^10^. Together, these results may provide new avenues for adaptive neoadjuvant studies based on ctDNA.

Translational analyses of colon tumours and the surrounding TME both before and after neoadjuvant ICB are of great interest to identify biomarkers of response to immunotherapy or chemotherapy, thereby aiding future selection of patients who are most likely to benefit from either of these therapies. The neoadjuvant administration of immunotherapy in this study provides the window of opportunity for a unique in-depth analysis of the colon cancer TME of responders versus non-responders. The lack of response in metastatic pMMR colon cancers has been largely attributed to their low TMB and lack of immune infiltration. Here we have demonstrated that deep pathological responses can be induced by ICB in pMMR colon cancer despite a low TMB in all tumours. Furthermore, established biomarkers predictive of response to immunotherapy, such as IFNγ^43,44^ and CD8^+^ T cell infiltration^45^, were not different between responders and non-responders at baseline, suggesting that in colon cancer, different mechanisms of immune evasion may dominate. In an exploratory analysis, we observed hints of an enrichment for response in tumours with *TP53* mutations, with a higher probability of response in those harbouring a *TP53* mutation without a *KRAS*^*G12*^ mutation. If validated in larger studies, these data may enable selection of patients based on standard-of-care genomic assessments of colorectal cancers. Consistent with an elevated response rate in tumours with *TP53* mutations, responding tumours also displayed an enrichment of whole-genome duplications, which has recently been linked to higher ICB responsiveness in other cancer types^27^.

In addition, proliferation and cell cycle gene signatures, as well as Ki-67^+^ tumour and CD8^+^ T cells, were found to be associated with response to ICB. Although higher baseline proliferation has been described in responders to ICB in other cancer types^44,46^, there have been no previous data directly connecting the triad of *TP53* mutational status, proliferation signatures and ICB response. Of note, both proliferation and whole-genome duplications can promote states of cell stress and marked chromosomal instability^25,27,47^, altered expression of antigens and activation of mechanisms for elimination of aneuploid cells^48,49^. This may in part explain responses observed in tumours with high levels of genomic instability in our study.

Together, these data provide early evidence for a possible role for ICB to enable immune targeting of genomically unstable *TP53*-mutant tumours. Of note, although genomic instability may potentiate ICB efficacy by exposing immune vulnerabilities, it may also lead to evolution of resistance mechanisms in tumour cells^48,50^. Gaining a better understanding of the intricate interactions between genomic instability, immunogenicity and immune evasion in the context of ICB will be of importance to further improve the treatment of pMMR CRC.

In line with the association of proliferation signatures and response, using IMC and scRNA-seq, we detected a higher infiltration of Ki-67^+^ and CD103^+^CD8^+^ T cells with features of dysfunctional/exhausted tumour-reactive cells in responders. These cells represented a relatively small proportion of the total T cell pool, and showed expression of *ITGAE* (encoding CD103), *CXCL13*, *ENTDP1*, *PDCD1* and other checkpoint molecules consistent with previous antigen exposure and tumour reactivity. These results are in line with reports of chronically stimulated tissue-resident CD8^+^ populations that retain proliferative capacity and are thought to be involved in tumour control^30,31^ and that have been associated with better prognosis and tumour reactivity in pMMR CRC^51,52^.

When comparing pre-treatment and post-treatment expression data, we found that ICB was able to induce substantial immune mobilization in both responding and non-responding tumours. These results suggest that lack of overall immune activation may not form the predominant mechanism underlying ICB resistance in non-metastatic pMMR colon cancer. The importance of non-immune TME components in response to ICB is suggested by our observation that stromal inflamed-fibrotic TME subtypes were associated with lack of response, whereas the magnitude of immune infiltration by itself was not. Compared with other subtypes, inflamed-fibrotic tumours exhibited higher signalling of TGFβ, a potent negative regulator of cancer immunity that has been associated with immune exclusion and poor prognosis. On the basis of these results and previous data showing an association between TGFβ and non-response in urothelial and gynaecological malignancies^53,54^, it may be of interest to explore the role of TGFβ in response to ICB in patients with pMMR colon cancer in future trials^46,55–58^.

Although a subset of patients with non-metastatic pMMR colon cancer is cured with standard-of-care adjuvant chemotherapy, depending on stage, between 20% and 50% of patients develop disease recurrence despite this treatment^59^. Within the NICHE platform study, we provide data demonstrating deep pathological responses following a short treatment with neoadjuvant ICB in 26% of patients with pMMR colon cancer^9,24^. On the basis our data, we envision prospective studies that stratify patients based on *TP53* and *KRAS* status to validate the results from the current study. Exploratory studies of treatments targeting KRAS and tumour stroma, including TGFβ, may in addition provide avenues for the subgroup of non-responders to ICB.

In conclusion, our data indicate that a substantial proportion of patients with localized pMMR colon cancer may benefit from neoadjuvant anti-PD1 plus anti-CTLA4. These promising clinical and translational findings warrant validation in larger cohorts of selected patient populations.

## Methods

### Patients

Eligible patients were 18 years of age or older and had previously untreated, pMMR, clinical stage I, II or III colon adenocarcinoma that was considered resectable and showed no signs of distant metastases. All patients had a World Health Organization (WHO) performance status of 0 or 1 and adequate haematological and end-organ function. MMR status was determined by immunohistochemical staining for the MMR proteins MLH1, PMS2, MSH2 and MSH6, and proficiency was defined as normal expression of all four proteins. Key exclusion criteria included signs of obstruction or perforation, previous immunotherapy, active autoimmune disease requiring systemic immunosuppressive treatment and active concurrent cancer.

### Study design

The NICHE study (ClinicalTrials.gov: NCT03026140) is an investigator-initiated, open-label study that was conducted at the Netherlands Cancer Institute (NKI). All patients received a single dose of ipilimumab 1 mg kg^−1^ on day 1 and two doses of nivolumab 3 mg kg^−1^ on day 1 and day 15, and patients were randomized to additionally receive celecoxib 200 mg daily from day 1 until the day before surgical resection. Surgery was scheduled in one of the participating centres with a predefined maximum of 6 weeks after study enrolment. All treatment cycles were administered before surgery and no standard adjuvant study treatment was given. According to national guidelines, adjuvant chemotherapy was offered to patients who had tumour-positive lymph nodes or ypT4 status in the post-treatment resection specimen.

### End points and statistics

The primary objectives were safety and feasibility. The primary objective of safety was evaluated by the occurrence of (serious) adverse events and the objective of feasibility was assessed by treatment-related complications leading to surgical delay past the planned 6 weeks after inclusion in the study, as well as by adherence to the study protocol. All patients who received at least once cycle of the study treatment were monitored for SAEs and adverse events until 100 days after study drug administration and all adverse events were graded according to the National Cancer Institute Common Terminology Criteria for Adverse Events (v4.03)^60^. Secondary and translational end points included DFS, overall survival and efficacy as measured by histopathological treatment response, as well as assessment of putative biomarkers of response and exploration of treatment-induced changes in the TME based on genomics, transcriptomics, immunohistochemistry and IMC.

Initial data regarding safety, efficacy and translational end points obtained from the first 17 patients enrolled in the pMMR cohort and treated with nivolumab plus ipilimumab with or without celecoxib have previously been published^24^. The current report includes data on both the previously published patients (*n* = 17) and the patients included thereafter (*n* = 16).

All patients underwent tumour staging at baseline by CT of the chest and abdomen performed within 28 days before the start of treatment. Colonoscopy to obtain representative pre-treatment biopsies from tumour and normal colon tissue was performed within 7 days before the first treatment cycle, and post-treatment tissue was obtained by surgical resection. All obtained tissue samples were either directly frozen or fixed in formalin and paraffin embedded.

Clinical data were collected using the TENALEA Clinical Trial Data Management system. Baseline characteristics are presented for the intention-to-treat population, defined as all patients enrolled in the study. Categorical variables are summarized as absolute numbers and percentages and continuous variables with medians and (interquartile) ranges. For binary outcomes, exact two-sided 95% CIs were calculated using the Clopper–Pearson method. Time-to-event end points include DFS and overall survival. DFS was defined as the time from surgery to recurrence or disease-related death; patients alive at the last follow-up date who did not experience progression or recurrence were censored. Overall survival was defined as the time between the date of enrolment and death due to any cause; data for patients who are not reported as having died have been censored at the date when last known to be alive. The Kaplan–Meier method was used to analyse time-to-event end points. A log-rank test was used to compare DFS and overall survival curves between responders and non-responders; for comparison of the overall survival curves, landmark analysis was performed with a landmark at the date of surgery. Median follow-up time from enrolment was calculated using the reverse Kaplan–Meier method. All reported *P* values are two-sided unless otherwise specified, and a *P* < 0.05 was considered statistically significant. Analyses were performed using R (v4.3.0)^61^ using R-studio build 561 with packages: arsenal (v3.6.3), survival (v3.6-4) and survminer (v.0.4.9), except for statistical analyses related to RNA-seq, whole-exome sequencing, immunohistochemistry and IMC data, which were conducted using R (v4.2.3) using R-studio build 513 with the packages: tidyverse (v2.0), ggplot2 (v3.4.2), ggpubr (v0.6.0) and pheatmap (v1.0.12). Exploratory comparisons of signature expression scores and genomic features were performed between responders and non-responders using two-sided Wilcoxon rank-sum tests. Comparisons between pre-treatment and post-treatment samples were tested using Wilcoxon signed-rank tests. Multiple hypothesis testing correction was performed with the method from Benjamini–Hochberg. Nominal *P* values are provided and values corresponding to 0.33 FDR are indicated. Each patient represented a unit of analysis.

### Study oversight

The study protocol was approved by the institutional review board of the NKI (sponsor) and by the individual ethics boards of participating centres: OLVG and Spaarne Gasthuis. The study was conducted in accordance with the International Conference on Harmonization Guideline for Good Clinical Practice and the principles of the Declaration of Helsinki. Written informed consent for participation in the study was obtained from all patients.

### Pathology assessments and immunohistochemistry

Formalin-fixed, paraffin-embedded (FFPE) blocks were obtained from pre-treatment biopsies and from post-treatment surgical specimens. Baseline tumour biopsies were used to assess MMR status by immunohistochemistry for MLH1, PMS2, MSH2 and MSH6 performed on a BenchMark Ultra autostainer (Ventana Medical Systems) following the manufacturer’s instructions. In brief, 3-µm sections were cut from FFPE blocks, which were heated for 28 min at 75 °C and deparaffinized using EZ Prep solution (Ventana Medical Systems). Heat-induced antigen retrieval was performed for 32 min at 95 °C using Cell Conditioning Solution 1 (Ventana Medical Systems). The antibodies used for staining were MLH1, ready-to-use, M1 (6472966001, Roche); PMS2, 1:40 dilution, clone EP51 (M3647, Agilent Technologies); MSH2, ready-to-use, G219-1129 (5269270001, Roche); and MSH6, 1:50 dilution, EP49 (AC-0047, Abcam). The OptiView DAB Detection Kit was used to visualize bound antibody and slides were counterstained with haematoxylin and bluing reagent (Ventana Medical Systems). Individual stains for each protein were regarded as positive in case of clear nuclear staining in tumour cells with a positive internal control. Tumours with positive staining for all four proteins were considered MMR proficient.

Post-treatment surgical specimens were subjected to histopathological examination using the entire tumour bed and all resected lymph nodes. Slides were counterstained with haematoxylin and eosin (H&E) to assess the percentage of RVT^62^. Pathological response was defined as 50% or less RVT (consistent with AJCC/College of American Pathologists (CAP) TRG 0-2)^63^, including partial response with 11–50% RVT (AJCC/CAP TRG 2), MPR with 10% or less RVT (AJCC/CAP TRG 0-1) and pCR with absence of RVT in both the tumour bed and the lymph nodes (AJCC/CAP TRG 0)^62^. Post-treatment specimens were also staged according to the AJCC 8th TNM classification^64^. All specimens were centrally reviewed by an experienced gastrointestinal pathologist.

FFPE sections obtained from pre-treatment tumour biopsies were evaluated by immunohistochemistry for PD1 and CD8 using a Discovery Ultra autostainer (Ventana Medical Systems). In brief, FFPE blocks were used to cut 3-µm sections, which were heated for 28 min at 75 °C and deparaffinized using EZ Prep solution (Ventana Medical Systems). Heat-induced antigen retrieval was performed for 32 min at 95 °C using Cell Conditioning Solution 1 (Ventana Medical Systems). A double stain was performed for PD1 (yellow) followed by CD8 (purple). In the first sequence, PD1 was detected using clone NAT105 (ready-to-use, Roche Diagnostics, Ventana Medical Systems) for 32 min at 37 °C, and bound antibody was visualized using anti-mouse NP (Ventana Medical Systems) for 12 min at 37 °C, followed by the Discovery Yellow Detection Kit (Ventana Medical Systems). In the second sequence, CD8 was detected using clone CD8/144B (1:200 dilution; Agilent/DAKO) at 32 min for 37 °C, and bound antibody was visualized using anti-mouse HQ (Ventana Medical Systems) for 12 min at 37 °C, followed by the Discovery Purple Detection Kit (Ventana Medical Systems). Slides were counterstained with haematoxylin and bluing reagent (Ventana Medical Systems). All slides were scanned with the Panoramic 1000 (3D HISTECH) at 0.25 µm per pixel. HALO image analysis software (v4.0.5107.357; Indica Labs)^65^ was used for quantification of positively stained cells within the tumour region that was manually annotated by a gastrointestinal pathologist. The Indica Labs Multiplex IHC (v3.0.3) analysis algorithm was used for the analysis.

### ctDNA analysis

Signatera (Natera Inc.) ctDNA analysis was performed retrospectively on banked specimens. Whole-exome sequencing was conducted on tumour tissue and on matched germline DNA obtained from peripheral blood mononuclear cells. Following initial quality controls and sample concordance checks, somatic variant calling was performed using Natera’s propriety bioinformatics pipeline^66^, which allows filtering of putative germline and clonal haematopoiesis mutations of unknown significance. Up to 16 somatic single-nucleotide variants were selected, based on which PCR amplicons were designed and applied on cell-free DNA samples of all patients.

A median of 29.25 ng cell-free DNA was isolated from a median of 4.0 ml (range 1.7–7.4 ml) plasma obtained from all patients at baseline, before cycle 2, pre-operative as well as 3 weeks and 3–6 months post-operative. Plasma samples were considered to be ctDNA^+^ when 2 or more out of 16 variants were detected. The concentration of ctDNA in each sample was measured and reported as mean tumour molecules per millilitre of plasma.

Categorical variables were compared using Fisher’s exact test, carried out in R (v4.3.1) using packages stats (v4.3.1) and mosaic (v1.9.1).

### IMC staining and analysis

IMC was performed on pre-treatment biopsies using 4-µm-thick FFPE tissue sections to detect 40 cellular targets as previously described^67^. After deparaffinizing and heat-mediated antigen retrieval in high pH antigen retrieval solution (eBioscience, Thermo Fisher Scientific), sections were subjected to blocking with Superblock solution (Thermo Fisher Scientific) to limit unspecific binding of antibodies. Next, antibodies were incubated overnight at 4 °C with anti-CD4 and anti-TCRγδ (antibody details are available in Supplementary Table 3) followed by a 5-h room temperature incubation with the first set of antibodies. After rigorous washing, the sections were incubated with the remaining antibodies and incubated for 5 min with DNA intercalator iridium (1.25 µM; Fluidigm) to stain the nuclei. Finally, sections were washed with water, air dried and stored at room temperature until measurement.

For each section, two or three 1 mm^2^ regions of interest were ablated on the Hyperion mass cytometry imaging system (Fluidigm). Data quality was visually inspected using the Fluidigm MCD viewer (v1.0.560.6) and exported as multi-tiff files. Images were normalized by rescaling all images and markers between 0 and 1 followed by a two-step denoising, where first a minimal signal threshold of 0.1 was set followed by per marker percentile normalization. Cell segmentation masks were generated from the normalized images using CellProfiler (v4.2.1). First nuclei were defined using the DNA images to which membranes were added using keratin, vimentin and CD45 images. Single-cell marker expression FCS files were generated by combining the normalized images with cell segmentation masks in ImaCytE^68^ and after dimensionality reduction, cells were clustered by mean-shift clustering in Cytosplore (v2.3.1)^69^. Clusters were mapped back on the images and visually confirmed by comparison with raw images in MCD viewer. Finally, cluster abundances per image were combined per sample and visualized as cells per mm^2^. Counts were averaged across regions of interest for each sample. Owing to low abundance of CD103 and granzyme B, no distinct clusters were formed and thus their presence was determined by counting the number of cells with a marker expression above 0.2 in each T cell cluster.

### Genomic and transcriptomic analysis

Whole-exome sequencing was conducted on tumour DNA isolated from pre-treatment biopsies and matched germline DNA from peripheral blood samples to assess the mutational landscape. RNA was isolated from pre-treatment and post-treatment tumour samples and was sequenced to evaluate expression of various immune-related and tumour-related gene signatures.

Fresh-frozen tumour samples obtained pre-treatment and post-treatment were used for DNA and RNA isolation. A cryostat was used to cut 10-µm sections intended for isolation and a consecutive 5-µm section used to initially assess tumour percentage. This 5-µm section was H&E stained and a pathologist scored tumour cell percentage and indicated the relevant tumour region. Samples were selected for isolation using a tumour percentage of at least 30%, except for post-treatment samples from tumours with an MPR or pCR. DNA and RNA were isolated simultaneously from 10-µm sections with the AllPrep DNA/RNA/miRNA Universal isolation kit (80224, Qiagen) by using the QIAcube, according to the manufacturer’s protocol. If fresh-frozen material was unavailable or if tumour content was insufficient, FFPE sections were used instead. A pathologist scored tumour percentage and indicated the most tumour-dense region for isolation on an H&E-stained slide. Depending on tumour size, 5–10 10-µm sections were cut and DNA and RNA were isolated simultaneously with the AllPrep DNA/RNA FFPE isolation kit (80234, Qiagen) using the QIAcube, according to the manufacturer’s protocol. Normal germline DNA was obtained from 1 ml peripheral blood and was extracted by using the QIAsymphony DNA Blood 1000 (DNA Midi kit, 96), according to the manufacturer’s protocol.

### DNA-seq and analysis

The concentration of double-stranded DNA was quantified in each sample using the Qubit dsDNA HS Assay Kit (Q32851, Invitrogen). Covaris AFA technology was used to fragment a maximum amount of 2 μg double-stranded genomic DNA into fragment sizes of 200–300 bp. Sample purification was carried out using Agencourt AMPure XP Reagent (A63881, Beckman Coulter) in 2× reaction volume settings according to the manufacturer’s instructions. Quantity and quality of fragmented DNA was measured on a BioAnalyzer system using the DNA7500 assay kit (5067-1506, Agilent Technologies). Next-generation sequencing library preparation for Illumina sequencing was conducted with the KAPA HTP Prep Kit (KK8234, KAPA Biosystems) using xGen UDI-UMI Adapters of IDT 10 bp (Integrated DNA Technologies). A four-cycle PCR was performed to amplify libraries to obtain sufficient yield for exome enrichment. Quantification of DNA libraries was done on a BioAnalyzer system using the DNA7500 assay kit. Exome enrichment was conducted on library pools of eight unique dual-indexed libraries (500 ng each) using the xGen Exome Hyb Panel v2 (10005152, Integrated DNA Technologies) and xGen Hybridization Capture Core Reagents following the manufacturer’s instructions in which hybridization time was adjusted to 20 h and 10 cycles of PCR were performed during post-capture PCR. All exome-enriched library pools were quantified on a BioAnalyzer system with the DNA7500 assay kit, pooled equimolar to a 10 nM final concentration, followed by paired-end 100-bp sequencing on either an Illumina HiSeq 2500 using V2 chemistry or an Illumina Novaseq 6000 instrument using a NovaSeq 6000 S4 Reagent Kit v1.5 (20028313, Illumina) and a S2 Reagent kit v1.5 (20028315, Illumina), according to the manufacturer’s protocol.

Whole-exome sequencing data were processed using Sarek (v3.1.2)^70^, a pipeline that follows GATK best practices and is distributed by NF-core. Samples were aligned to GRCh38 using bwa (v0.7.17), duplicates were marked with MarkDuplicates (GATK pipeline v4.3) and base-quality scores were recalibrated with BaseRecalibrator (GATK pipeline v4.3). Germline and somatic indels and single-nucleotide variants were called with Strelka2 (v2.9.10)^71^, annotated with snpeff (v5.1) and ensemblvep (v106.1). Only variants with at least 20× depth and 5% variant allele frequency were considered. TMB was calculated as the number of non-synonymous and frameshift variants divided by the target BED coverage size (39 Mb).

For assessing the functional impact of mutations, variants were filtered based on the following criteria: mutations were considered if they had moderate or high impact by snpeff or consensual (likely) pathogenic annotations in ClinVar^72^, whereas variants with only benign and likely benign annotations were removed. Variants of either uncertain significance, conflicting evidence (for example, pathogenic and benign) or no ClinVar data available were considered if structural predictions were deleterious or damaging, with either SIFT scores below 0.05 or PolyPhen-2 scores above 0.446. Mutated genes were only included on heatmaps and considered functionally affected if they passed these filters. Furthermore, non-synonymous mutations were converted with vcf2maf (v1.6.22) and annotated with OncoKB annotator (v3.4.1) to obtain gain-of-function and loss-of-function OncoKB annotations^73^. Copy number variation calling, genome integrity scores and whole-genome duplication states were obtained with ASCAT (v3.0)^74^. ASCAT genome integrity scores measure the fraction of the copy-number profile deviating from a ploidy of 2*n* or deviating from 4*n* for whole-genome-duplicated tumours. In addition, the weighted genome integrity index^25,26^ was used as a surrogate for chromosomal instability with a cut-off of 0.2. This was calculated as the percentage of ASCAT heterozygous sites deviating from the median baseline ploidy by at least 0.6, averaged across the 22 autosomal chromosomes.

### Bulk RNA-seq and analysis

The quality and quantity of the total RNA were assessed on the 2100 Bioanalyzer instrument using an Agilent RNA 6000 Nano chip (G2938-90034, Agilent Technologies) following the manufacturer’s instructions. The region method analysis was used according to the manufacturer’s manual (technical-note-470-2014-001, Illumina) to determine the percentage of RNA fragments with more than 200-nt fragment distribution values (DV200).

Strand-specific libraries were generated using the TruSeq RNA Exome Library Prep Kit (Illumina) according to the manufacturer’s instructions (1000000039582v01, Illumina), with the following adaptation: samples that contained intact total RNA were subjected to the optional heat fragmentation step (94 °C for 8 min, 4 °C hold). For the initial 15 pre-treatment and 14 post-treatment samples, the generated complementary DNA (cDNA) fragments were ligated to TruSeq RNA Single Indexes adapters (20020492/20020493, Illumina), and for the remaining samples, IDT xGen UDI(10 bp)-UMI(9 bp) paired-end sequencing adapters (Integrated DNA Technologies) were used. All cDNA fragments were amplified by 15 cycles of PCR. The libraries were validated on a 2100 Bioanalyzer instrument following the manufacturer’s protocol of the Agilent DNA 7500 kit (G2938-90025, Agilent Technologies), followed by a 1–4 plex library pooling containing up to 200 ng of each sample. The pooled cDNA libraries were enriched for target regions using the probe Coding Exome Oligos set (CEX, 45 Mb, Illumina) and 10 cycles of PCR. The libraries were analysed on a 2100 Bioanalyzer instrument following the manufacturer’s protocol of the Agilent DNA 7500 kit (G2938-90025, Agilent Technologies), then diluted to 10 nM and pooled equimolar into multiplex sequencing pools. Libraries of the initial 15 pre-treatment and 14 post-treatment samples were sequenced with 65 base single reads on a HiSeq2500 using V4 chemistry (Illumina) and demultiplexed using bcl2fastq2. For the remaining samples, the libraries were sequenced with 54 cycles for read 1, 19 cycles for read i7, 10 cycles for read i5 and 54 cycles for read 2 on a NovaSeq6000 using a Reagent Kit v1.5 (100 cycles; Illumina). Reads were demultiplexed using bclconvert and duplicates were marked with rumidup (https://github.com/NKI-GCF/rumidup).

FASTQ files were aligned to GRCh38 using Hisat2 (v2.2.1), duplicate and strand-aware counted per gene using gensum (https://github.com/NKI-GCF/gensum), and annotated with Ensembl GRCh38.107. Differential expression was performed with DESeq2 (v1.38.3) and enrichment analysis was performed with enrichR (v3.2) using genes with adjusted *P* < 0.05. ssGSEA scores for curated signatures (Supplementary Table 2) were calculated using the GSVA (v1.46) package and gene sets consisting of individual genes were compared using log_2_(reads per million + 1) values instead. Standardized mean differences (Cohen’s *d*) between response groups were calculated using the pooled standard deviation. Paired treatment effect sizes were calculated as the mean difference between pairs divided by the standard deviation. Pearson’s *r* correlations were calculated using ggpubr (v0.6.0), and fits and confidence intervals from linear models were visualized with the geom_smooth (v3.4.2). TME subtypes were classified manually based on *Z*-score heatmaps for Bagaev et al. signatures^32^. This was aided by using hierarchical clustering and median signature scores, blinded to response groups. CMS assignment was performed with the CMSclassifier random forest using the nearest CMS classification (https://github.com/Sage-Bionetworks/CMSclassifier).

### scRNA and TCR sequencing

Fresh tumour biopsies were transferred onto ice in RPMI 1640 medium (Thermo Fisher Scientific) supplemented with 2.5% fetal calf serum (FCS; Sigma-Aldrich) and 1% penicillin–streptomycin (pen–strep; Roche). On ice, the samples were placed in petri dishes and dissected by hand into small fragments of approximately 1–2 mm^3^ in size. These fragments were then suspended in 1 ml of freezing solution consisting of FCS containing 10% dimethyl sulfoxide (Sigma-Aldrich) and stored in liquid nitrogen for long-term preservation.

At the day of sequencing, cryopreserved tumour fragments were rapidly thawed in a 37 °C water bath until only a small ice crystal remained. To eliminate residual dimethyl sulfoxide, the fragments were subsequently washed three times using 7 ml wash medium (Dulbecco’s modified eagle medium supplemented with 10% FCS and 1% pen–strep) using six-well plates and 45-μm cell strainers. After this washing step, fragments were left on ice until all samples were thawed. Subsequently, samples were digested in 2 ml digestion medium consisting of RPMI 1640 supplemented with 1% pen–strep, pulmozyme (12.6 µg ml^−1^; Roche) and collagenase type IV (1 mg ml^−1^; Sigma-Aldrich) at 37 °C with continuous rotation for 45 min. Next, samples were washed twice with cold phosphate-buffered saline (PBS) and transferred to 1.5-ml Eppendorf tubes. Next, cells were resuspended in 25 µl of cold Cell Staining Buffer (BioLegend) containing Human TruStain FcX (1:10 dilution; BioLegend) to block Fc receptors. For sample multiplexing, TotalSeq-C anti-human hashtag antibodies (numbers 1–13; final concentration 1 μg ml^−1^; BioLegend) were added and incubated on ice for 30 min. Subsequently, 25 µl of Cell Staining Buffer containing anti-CD45-PerCP-Cy5.5 (1:50; Invitrogen) and TotalSeq-C-conjugated antibodies targeting PD1 (clone EH12-EH7, 1:1,000), CD39 (clone A1, 1:1,000), CD137 (clone 4B4-1, 1:5,000), CD8 (clone SK1, 1:5,000) and CD4 (clone RPA-T4, 1:2,500) were added. After another 30-min incubation on ice, cells were washed three times using Cell Staining Buffer. The cells were then resuspended in 500 µl MACS buffer (PBS with 0.5% bovine serum albumin (BSA; Sigma) and 2 mM EDTA (Life Technologies)). To normalize cell input across samples, 5 µl aliquots were taken and quantified using AccuCount Blank Particles (13.0–17.9 µm; Spherotech) on a BD LSR Fortessa X-20 Cell Analyzer (BD Biosciences). Dead cells were excluded using propidium iodide (0.5 µg ml^−1^; Sigma-Aldrich). Samples labelled with hashtag antibodies were then pooled, and live CD45^+^ immune cells were sorted using a FACSAria Fusion Flow Cytometer (BD Biosciences). Sorted cells were collected in RPMI 1640 medium containing 10% human serum and 1% pen–strep, followed by sequential washes with cold 1% BSA in PBS and 0.04% BSA in PBS. Finally, cells were resuspended in 0.04% BSA in PBS at a target concentration of 800–1,200 cells per microlitre, suitable for downstream scRNA-seq and TCR-seq using the 10X Genomics platform.

Between 10,000 and 20,000 sorted immune cells were loaded per lane on the 10X Chromium platform for scRNA-seq. For each run, cells from 3 to 13 different biopsies were combined. Library preparation for gene expression, TCR and antibody-derived tags was performed following the manufacturer’s protocol using the Chromium Next GEM Single Cell V(D)J Reagent Kits (10X Genomics). 10X 5′ single-cell sequencing was carried out on Illumina NovaSeq 6000 instrument, with target read lengths of 26–28/58–130 bp in RNA and antibody libraries, and 26–28/92–130 bp for TCR libraries. The sequencing depth was optimized to approximately 30,000 paired-end reads per cell for transcriptomic data and 5,000 reads per cell for both antibody and TCR libraries.

### scRNA and TCR sequencing data processing

Cell Ranger multi (v7.1.0) was used to generate the gene and feature counts mapping to GRCh38-2020-A as well as the VDJ assembly and clonotype assignment. The reference for the clonotype calling was generated from the IMGT database (LIGM-DB 14.1), using the script provided by 10X Genomics. Single-cell data processing was done using R (v4.2.3), R-studio (Build 513) and the Seurat package (v5.2.1)^75^. TCR data were analysed and integrated using scRepertoire (v2.3.2)^76^.

Detected cells were filtered for quality control based on overall median values for genes, counts, mitochondrial (‘^MT-’) and ribosomal protein RNA (‘^RP[SL]’) percentages. Cells passing quality control had the number of genes and counts within the range of 1/5 up to 5 median values, below 5 medians of mitochondrial percentage and above 1/3 of the median ribosomal protein RNA percentage. In addition, cells were filtered for a minimum of 200 genes, 1,000 counts and a maximum of 30% mitochondrial RNA percentage. The hashtag information in the feature counts was used to demultiplex filtered cells using HTODemux, keeping singlet calls and excluding doublets. scRNA-seq data were log normalized using the median library size as a scale factor, the top 2,000 variable features were obtained, and the full dataset was scaled. Principal component analysis was performed using 25 principal components, used for uniform manifold approximation and projection representations. Cells were initially clustered using the FindNeighbours and FindClusters functions at a resolution of 0.5. Broad cell-type annotations were assigned per cluster using knowledge base markers from Spectra^77^ and Cellmarker^78^, and also informed by automated scType labels. T and T/natural killer populations, corresponding to the set of non B-lineage lymphocytes, were separated and reclustered at different resolutions. All TCR^+^ cells were assigned with CD4^+^ and CD8^+^ annotations based on normalized antibody counts and *CD8A*/*CD8B* expression. TCR^−^ cells were annotated as CD8^+^ T cells if they contained *CD8A*/*CD8B* counts or CD8 antibody expression and CD4^+^ T cells if they contained *CD4* expression or antibody counts. CD8^+^ T cells, CD4^+^ T cells and other lymphocytes were separated, reclustered and annotated using knowledge base markers and signature scores, measured using AddModuleScore_UCell (Supplementary Table 2).

### External datasets

Analysis of TCGA and AC-ICAM cohorts^29^ was included for comparisons with external data of pMMR colon cancer. Processed RNA-seq and DNA-seq data were downloaded using cBioportal^79^. Mutational status for the TCGA cohort was based on analysis from Grasso et al.^80^. Samples with AJCC pathological stage IV colon cancer or *POLE* mutations were excluded. For TCGA cohort, genomic and transcriptomic data were available for 287 and 276 patients, respectively. For the AC-ICAM cohort, genomic and transcriptomic data were available for 173 patients with stage I–III pMMR colon cancer.

### Reporting summary

Further information on research design is available in the Nature Portfolio Reporting Summary linked to this article.

## Online content

Any methods, additional references, Nature Portfolio reporting summaries, source data, extended data, supplementary information, acknowledgements, peer review information; details of author contributions and competing interests; and statements of data and code availability are available at 10.1038/s41586-025-09679-4.

## Supplementary information

Supplementary Information NICHE Study Protocol.

Reporting Summary

Supplementary Table 1 Annotated tumour somatic mutations.

Supplementary Table 2 Immune and tumour-related gene sets for RNA sequencing analyses.

Supplementary Table 3 Antibody panel used for imaging mass cytometry.

## Figures and Tables

**Extended Data Table 1:**
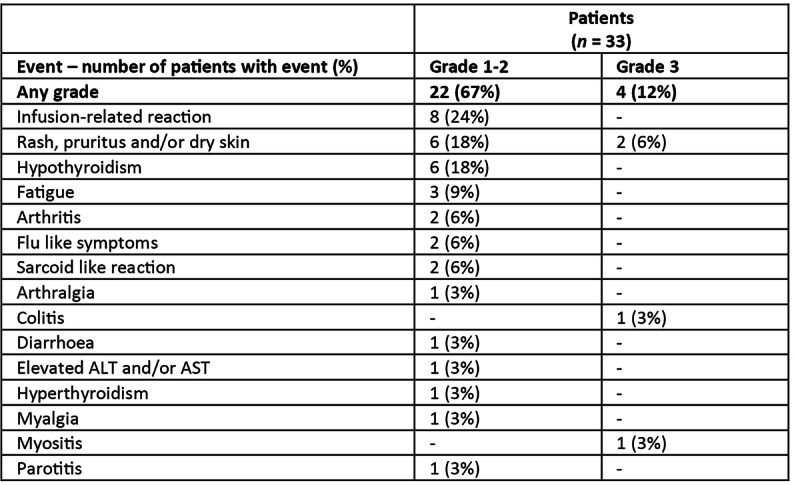
Immune related adverse events in all treated patients

All adverse events that were deemed at least possibly related to nivolumab and/or ipilimumab are presented in this table. This is regardless of the possible relationship with celecoxib.

**Extended Data Table 2:**
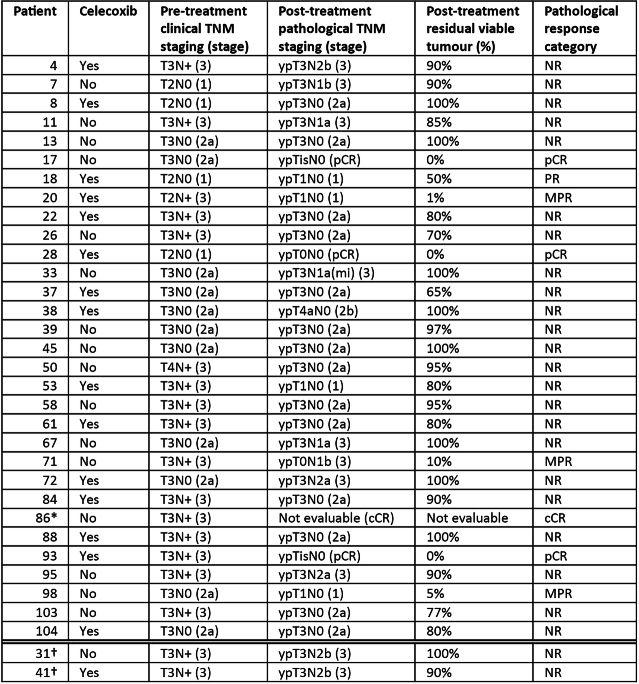
Clinical staging and histopathological findings per patient

* Patient 86 did not undergo surgery and had a clinical complete response based on radiographic evaluation and tumour-free biopsies. † Patients 31 and 41 retrospectivey did not meet inclusion criteria and were excluded from the efficacy analysis; 31 due to liver metastases at baseline and 41 due to having a mixed adenocarcinoma and neuroendocrine carcinoma (MiNEN). Both patients received adjuvant chemotherapy due to tumour positive lymph nodes in the resection specimen.

Abbreviations: NR: non-responder; PR: partial responder; MPR; major pathologic response; pCR: pathological complete response; cCR: clinical complete response.

**Extended Data Table 3:**
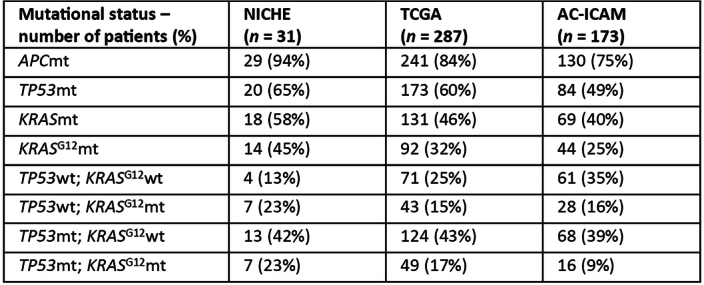
*TP53* and *KRAS* mutational status in patients with non-metastatic pMMR colon cancer in NICHE, TCGA and AC-ICAM cohorts

## Data Availability

DNA-seq and RNA-seq data for the NICHE study have been deposited in the European Genome-Phenome Archive (EGA) under the accession number EGAS50000000856. Data are under controlled access according to consent provided by the patients whose samples are used and according to GDPR. Data will be made available for academic use only upon reasonable request and within the confinements of the informed consent and the European Data Protection Regulation. Requests should include project descriptions, describing the research goal, privacy, governance and intended use of data, and can be done through the EGA (https://ega.nki.nl/), contacting repository@nki.nl. Requests will be reviewed by the institutional review board of the NKI and require signing of a data access agreement with the NKI after approval. The estimated time to response is 4–6 weeks with an expected total turnaround time of 4–6 months including drafting and approval of the data transfer agreement. Clinical data from TCGA Research Network were obtained from the clinical data resource from Liu et al.^81^. TCGA mutational status for CRC was obtained from analysis by Grasso et al.^80^, available as supplementary material (10.1158/2159-8290.CD-17-1327). RNA-seq data are openly available and were obtained from cBioportal (https://www.cbioportal.org), with the accession code coadread_tcga_pan_can_atlas_2018. Data for the AC-ICAM colon cancer cohort are openly available and were downloaded from cBioportal, with the accession code coad_silu_2022.
