# Supplementary material for: Neoadjuvant immunotherapy in mismatch-repair-proficient colon cancers
Source: Nature. 2025 Oct 20;648(8094):726–35. doi: 10.1038/s41586-025-09679-4 (PMC12711568; doi:10.1038/s41586-025-09679-4)
Supplement: Supplementary file 1 — NICHE Study Protocol. [file 41586_2025_9679_MOESM1_ESM.pdf]

nature portfolio

https://doi.org/10.1038/s41586-025-09679-4

## Supplementary information

# Neoadjuvant immunotherapy in mismatch-repair-proficient colon cancers

In the format provided by the
authors and unedited

# Nivolumab, Ipilimumab and COX2-inhibition in early stage colon cancer: an unbiased approach for signals of sensitivity.

# The NICHE TRIAL


**Principal investigators** Drs. M. Chalabi
[redacted]

**Co-investigators**

Quality control [redacted]
Nurse practitioner [redacted], [redacted]
Medical Oncology [redacted]
Gastro-enterology [redacted], [redacted], [redacted], [redacted], [redacted]
Surgery [redacted], [redacted]
Pathology [redacted], [redacted]
Immunology [redacted]
Biometrics [redacted]

**Protocol title**

**N**ivolumab, **i**pilimumab and COX2-inhibition in early stage **c**olon cancer: an unbiased approac**h** for signals of s**e**nsitivity. The NICHE trial.

| | |
|---|---|
| **Protocol ID/NKI study number**<br>**BMS study number** | N16NCI<br>CA209-832 |
| **Short title** | Pre-operative nivolumab, ipilimumab and COX2-inhibition in colon cancer (NICHE) |
| **EudraCT number** | 2016-002940-17 |
| **Version** | 1.1 |
| **Date** | october 2016 |
| **Coordinating investigator/project leader** | Drs. M. Chalabi |
| **Principal investigators** | Drs. M. Chalabi<br>[redacted] |
| **Sponsor** | Netherlands Cancer Institute |
| **Subsidising party** | Bristol-Myers Squibb |
| **Independent expert** | [redacted] |
| **Laboratory sites** | Not applicable |
| **Pharmacy** | Pharmacy Netherlands Cancer Institute |

## PROTOCOL SIGNATURE SHEET

| Name | Signature | Date |
|---|---|---|
| **Sponsor or legal representative:**<br>[redacted]<br><br>**Head of Department:**<br>[redacted] | | |
| **Coordinating Investigator**<br>***drs. M. Chalabi*** | | |

**TABLE OF CONTENTS**


1. INTRODUCTION AND RATIONALE ... 16
2. OBJECTIVES ... 20
3. STUDY DESIGN ... 22
4. STUDY POPULATION ... 25
   4.1. Population (base) ... 25
   4.2. Sample size calculation ... 27
5. TREATMENT OF SUBJECTS ... 28
   5.1. Investigational product/treatment ... 28
   5.2. Prohibited medications ... 28
   5.3. Escape medication ... 28
6. INVESTIGATIONAL PRODUCT ... 33
   6.1. Name and description of investigational product(s) ... 33
   6.2. Summary of findings from non-clinical studies ... 37
   6.3. Summary of findings from clinical studies ... 38
   6.4. Summary of known and potential risks and benefits ... 39
   6.5. Description and justification of route of administration and dosage ... 39
   6.6. Dosages, dosage modifications and method of administration ... 40
   6.7. Preparation and labelling of Investigational Medicinal Product ... 40
   6.8. Drug accountability ... 40
7. METHODS ... 40
   7.1. Study parameters/endpoints ... 40
      7.1.1. Main study parameter/endpoint ... 40
      7.1.2. Secondary study parameters/endpoints (if applicable) ... 40
   7.2. Randomisation, blinding and treatment allocation ... 41
   7.3. Study procedures ... 41
   7.4. Withdrawal of individual subjects ... 45
   7.5. Replacement of individual subjects after withdrawal ... 45
   7.6. Follow-up of subjects withdrawn from treatment ... 46
8. SAFETY REPORTING ... 49
   8.1. Section 10 WMO event ... 49
   8.2. AEs, SAEs and SUSARs ... 49
      8.2.1. Adverse events (AEs) ... 49
      8.2.2. Serious adverse events (SAEs) ... 50
      8.2.3. Suspected unexpected serious adverse reactions (SUSARs) ... 51
   8.3. Annual safety report ... 52
   8.4. Follow-up of adverse events ... 52
   8.5 Data safety monitoring board (DSMB) / Safety Committee ... 52
9. STATISTICAL ANALYSIS ... 53
   9.1. Primary study parameters ... 54
   9.2. Secondary study parameters ... 54
10. ETHICAL CONSIDERATIONS ... 58

10.1. Regulation statement ............ 58
10.2. Recruitment and consent ............ 58
10.3. Benefits and risks assessment, group relatedness ............ 58
10.4. Compensation for injury ............ 61
10.5 Incentives ............ 59
11. ADMINISTRATIVE ASPECTS, MONITORING AND PUBLICATION ............ 60
11.1. Handling and storage of data and documents ............ 60
11.2. Monitoring and Quality Assurance ............ 60
11.3 Registration of patients ............ 60
11.4 Storage of patient material ............ 60
11.5 Datamanagement ............ 61
11.6. Amendments ............ 61
11.7. Annual progress report ............ 61
11.8. End of study report ............ 62
11.9. Public disclosure and publication policy ............ 62
Appendix A: Performance Status Criteria ............ 63
Appendix B: study medication preparation, handling and administration guidance ............ 64
Appendix C: management algorithms ............ 65
Appendix D: immunoscore ............ 72
12. REFERENCES ............ 73

## LIST OF ABBREVIATIONS AND RELEVANT DEFINITIONS

| | |
|---|---|
| **ABR** | **ABR form, General Assessment and Registration form, is the application form that is required for submission to the accredited Ethics Committee (In Dutch, ABR = Algemene Beoordeling en Registratie)** |
| **AE** | **Adverse Event** |
| **AR** | **Adverse Reaction** |
| **CA** | **Competent Authority** |
| **CCMO** | **Central Committee on Research Involving Human Subjects; in Dutch: Centrale Commissie Mensgebonden Onderzoek** |
| **CV** | **Curriculum Vitae** |
| **DSMB** | **Data Safety Monitoring Board** |
| **EU** | **European Union** |
| **EudraCT** | **European drug regulatory affairs Clinical Trials** |
| **GCP** | **Good Clinical Practice** |
| **IB** | **Investigator’s Brochure** |
| **IC** | **Informed Consent** |
| **IMP** | **Investigational Medicinal Product** |
| **IMPD** | **Investigational Medicinal Product Dossier** |
| **METC** | **Medical research ethics committee (MREC); in Dutch: medisch ethische toetsing commissie (METC)** |
| **(S)AE** | **(Serious) Adverse Event** |
| **SPC** | **Summary of Product Characteristics (in Dutch: officiële productinfomatie IB1-tekst)** |
| **Sponsor** | **The sponsor is the party that commissions the organisation or performance of the research, for example a pharmaceutical company, academic hospital, scientific organisation or investigator. A party that provides funding for a study but does not commission it is not regarded as the sponsor, but referred to as a subsidising party.** |

| | |
|---|---|
| **SUSAR** | **Suspected Unexpected Serious Adverse Reaction** |
| **Wbp** | **Personal Data Protection Act (in Dutch: Wet Bescherming Persoonsgevens)** |
| **WMO** | **Medical Research Involving Human Subjects Act (in Dutch: Wet Medisch-wetenschappelijk Onderzoek met Mensen** |
| **COX2-i** | **Cyclooxygenase 2 inhibitor** |
| **WES** | **Whole exome sequencing** |
| **MSS** | **Microsatellite stable** |
| **MSI** | **Microsatellite instability** |
| **DFS** | **Disease-free survival** |
| **TME** | **Tumor micro environment** |

## SUMMARY

**Rationale:** Immunotherapy using checkpoints blockade has shown promising results in many cancer types, including melanoma, lung and renal cell cancer. Even though some of the first findings on the role of immune surveillance in cancer were described in colorectal cancers (CRCs), checkpoint blockade has thus far shown limited responses in patients with CRC, except in the small subset of patients with tumors with microsatellite instability (MSI), in which response rates reached 40% on anti-PD1 monotherapy. [1-4] Interim results from Checkmate-142, a phase II study evaluating nivolumab or nivolumab plus ipilimumab in patients with mCRC, showed encouraging results and increased response rates for the combination in MSI tumors.[5]

For immunotherapy to induce cancer cell death, sufficient activation of the immune system is needed. T cell activation requires two signals. The first signal is delivered via T cell receptors (TCR) upon antigen presentation by major histocompatibility complex (MHC) on the antigen-presenting cell (APC). The second signal can be produced by a number of distinct molecular interactions that may occur between an APC and a T cell. These are the so-called immune checkpoints and can be either co-stimulatory or co-inhibitory. PD-1 and CTLA-4 immune checkpoint molecules are often upregulated in tumor infiltrating T cells and by binding their corresponding ligands, often upregulated on cancer cells, downregulate the T cell response. Nivolumab and ipilimumab enhance T-cell antitumor activity by non-redundant, complementary mechanisms, leading to activation of antitumor immunity.[6,7] Clinical experiences with nivolumab plus ipilimumab demonstrate deep and durable responses in previously treated melanoma and lung cancer.
The response to immune checkpoint blockade seen in CRCs with microsatellite instability (MSI) is largely absent in microsatellite stable (MSS) tumors. The mechanism by which MSS tumors are able to circumvent this response is not yet clear. Since MSI tumors comprise 15% of all CRCs, but only 5% in the metastatic disease setting, there is an unmet need for the treatment of patients with MSS tumors and deciphering the way in which this group might benefit from immunotherapy is imminent.[7] Strikingly, recent data has shown that the Immunoscore is a better predictor of disease-free and overall survival than MSI status.[6] Furthermore, preclinical work suggests that COX2-inhibition could help to circumvent tumor microenvironment-driven evasion of immunotherapy.[8,9] Further subgrouping and dissection of the characteristics of immunogenicity in the large, heterogeneous group of MSS tumors is needed, starting with a better understanding of CRC responses to and evasion of immunotherapy in the primary tumor.[10,11]

One of the bases for this study is the suggestion that the Immunoscore, a scoring system,

based on the quantification of cytotoxic and memory T cells in the core of the tumor (CT) and in the tumor's invasive margin (IM), may be more important than MSI status in determining sensitivity to immune checkpoint inhibitors, since recent data show that the Immunoscore is a better predictor of disease-free and overall survival than MSI status in CRCs. Preclinical work suggests that COX2-inhibition could help circumvent tumor microenvironment (TME)-driven evasion of immunotherapy.[8,9] Using MSI tumors as the comparators for the immune responses observed in MSS tumors will grant more insight and understanding of these escape mechanisms.

**Objectives:**

Primary Objective: To determine the safety and feasibility of pre-operative immunotherapy in CRC.

Secondary Objectives:

- To explore the immune activating capacity of short-term pre-operative immunotherapy and COX-2 inhibition in primary MSI and MSS colon cancers (CRCs) and identify underlying potential mechanisms of escape from the immune system.
- To assess the immunogenic mutational load by DNA WES and correlation with putative markers of response;
- To assess changes in immune suppressive pathways, IFNy induced gene expression by RNA sequencing;
- To determine pathological tumor changes following short-term immunotherapy;
- To assess relapse free survival;
- To explore the Immunoscore as a predictor of immunotherapy induced changes in the TME and correlation to putative markers of response;
- To explore the immunogenicity and in vitro T-cell sensitivity of tumor organoid cultures;

Additional detailed information on the translational secondary objectives can be found in the full protocol.

**Study design:** In this single-center, open-label, exploratory study, we will enroll 30 patients with MSS tumors and 30 patients with MSI tumors. Patients with MSS tumors will be randomized to either group 1 or 2 (figure 1). Patients with MSI tumors will all be allocated to group 1. Since the incidence of MSS tumors is higher, accrual for this group is expected to be faster than for the MSI group. After inclusion of 30 patients with MSS tumors, this cohort will be closed, after which only patients with MSI tumors will be included. Observations made in this cohort will mainly serve as positive comparators of a productive anti-tumor immune

response. Findings within the TME of MSI tumors will be the main source of information on the read-outs to be used for the TME of MSS tumors. The Immunoscore, using the scoring system by Galon et al[1], will be performed on all tumors using biopsy material. Based on recent findings, we expect 30-40% of MSS and 60-70% of MSI primary colon tumors to have high Immunoscores. Feasibility of the Immunoscore on tumor biopsies will be tested in a separate cohort. If the Immunoscore cannot be determined based on primary tumor biopsies, inclusion of patients with MSI tumors will be limited to 20 total.

**Study population:** In order to be eligible to participate in this study, a subject must meet all of the following criteria:

- Signed written informed consent;
- Patients at least 18 years of age;
- Stage 1-3 adenocarcinoma of the colon (and upper rectum/rectosigmoid considered as non-rectal and not undergoing neoadjuvant treatment);
- No signs of distant metastases on CT-scan and physical examination;
- No signs of obstruction or macroscopic bleeding;
- No clinical symptoms or radiological suspicion of perforation;
- Colonoscopy must be performed after registration to obtain study-specific biopsies. If biopsies are not possible, patients cannot be included in the study;
- WHO performance status of 0 or 1;
- Screening laboratory tests must meet the following criteria and should be obtained within 7 days prior to randomization/registration: WBC ≥ 2.0 x 10^9/L, ANC ≥ 1.5x10^9/L, platelets ≥ 100 x 10^9/L, Hemoglobin ≥ 5.0mmol/L. Transfusion is allowed to obtain an adequate hemoglobin level. Liver function tests: total bilirubin < 1.5 upper limit of normal (ULN) (except for subjects with Gilbert syndrome, who can have total bilirubin <3.0 mg/dL); alkaline phosphatase <2.5 ULN; transaminases (ASAT/ALAT) <3 x ULN; LDH < 2 x ULN;
- Creatinine clearance (Cockcroft-Gault) of ≥40 ml/min;
- Women of childbearing potential (WOCBP)* must use appropriate method(s) of contraception. WOCBP should use an adequate method to avoid pregnancy for 23 weeks (30 days plus the time required for nivolumab to undergo five half-lives) after the last dose of investigational drug;
- Women of childbearing potential must have a negative serum or urine pregnancy test (minimum sensitivity 25 IU/L or equivalent units of HCG) within 24 hours prior to the start of nivolumab;

- Men who are sexually active with WOCBP must use any contraceptive method with a failure rate of less than 1% per year. Men receiving nivolumab and who are sexually active with WOCBP will be instructed to adhere to contraception for a period of 31 weeks after the last dose of investigational product Women who are not of childbearing potential (i.e., who are postmenopausal or surgically sterile as well as azoospermic men do not require contraception;
- CT-scan must be performed within 28 days prior to registration;
- No previous treatment with immune checkpoint inhibitors targeting CTLA-4, PD-1 or PD-L1;
- For patients with MSS tumors: no current use of NSAIDs or COX2-inhibitors at registration and no active peptic ulcer, gastrointestinal bleeding, unstable ischemic heart disease of thrombus etiology or significant established ischemic heart disease, peripheral arterial disease and/or cerebrovascular disease;
- No radiotherapy prior to or planned post-surgery radiotherapy;
- Allergies and Adverse Drug Reaction
    - No history of allergy to study drug components
    - No history of severe hypersensitivity reaction to any monoclonal antibody
    - No history of allergy or severe hypersensitivity to NSAIDs or COX2-I (MSS tumors)
- No intercurrent illnesses, including but not limited to infections, unstable angina pectoris
- No underlying medical conditions that, in the Investigator's opinion, will make the administration of the study drug hazardous or obscure the interpretation of toxicity determination of adverse events;
- No positive test for hepatitis B virus surface antigen (HBsAg) or hepatitis C virus ribonucleic acid (HCV antibody) indicating acute or chronic infection;
- No history of testing positive for human immunodeficiency virus (HIV) or known acquired immunodeficiency syndrome (AIDS);
- No active autoimmune disease or a documented history of autoimmune disease, or other medical conditions requiring systemic steroid or immunosuppressive medications, except for subjects with vitiligo, diabetes mellitus type 1, residual hypothyroidism due to autoimmune condition only requiring hormone replacement, psoriasis or resolved childhood asthma/atopy not requiring systemic treatment;
- No conditions requiring systemic treatment with either corticosteroids (> 10 mg daily prednisone equivalents) or other immunosuppressive medications within 14 days of study drug administration. Inhaled or topical steroids and adrenal replacement doses >

10 mg daily prednisone equivalents are permitted in the absence of active autoimmune disease;

- No live vaccines in the 4 weeks prior to inclusion;
- No history of uncontrolled medical or psychiatric illness;
- No psychological, familial, sociological or geographical condition potentially hampering compliance with the study protocol and follow-up schedule;
- No current pregnancy or breastfeeding;
- No malignancies other than disease under study within 5 years prior to inclusion.

**Intervention:** Patients will be treated with short-term immunotherapy ± COX2-inhibitors. This treatment will be given during the window period until surgical resection of the tumor. The duration of treatment will be approximately 4 weeks.
After completion of accrual, analysis of the endpoints will be performed. Based on these findings and available compounds at that time, the study will be amended to include new drug combinations with one or more of the study drugs used in the first group of patients.

During a run-in period, three patients will be included and treated with nivolumab monotherapy. When this pre-operative treatment is deemed safe and feasible, as defined in the primary endpoint, accrual for the combination treatment as described below will be started.

Patients in **group 1** will be treated with a single dose of ipilimumab 1mg/kg on day 1 and two cycles of nivolumab 3mg/kg on day 1 and 15, respectively.
Patients in **group 2** will be treated with a single dose of ipilimumab 1mg/kg on day 1, two cycles of nivolumab 3mg/kg one day 1 and 15 and celecoxib 200mg once daily until the day before surgery (figure 1).

A minimum of 8 tumor biopsies is required at baseline, containing sufficient tumor content (≥30%), acquired through colonoscopy. Tumor and normal tissue will be collected at resection. One CT scan will be required at baseline. Blood draws (incl. PBMC, serum and plasma collection) will be required at baseline, before every cycle of therapy, peri-operatively and 3 weeks post-operatively.
***See table 1 of the full protocol.***

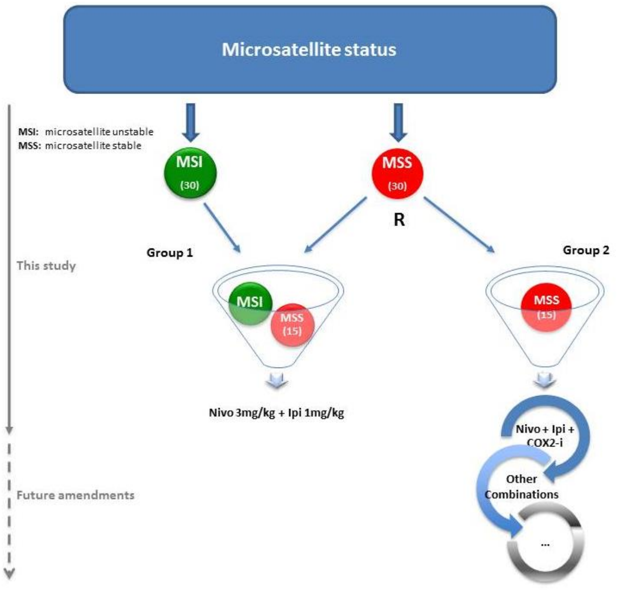


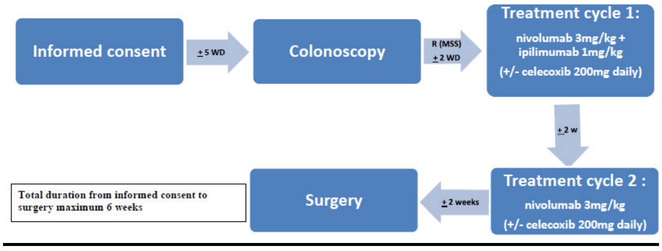


**Figure 1**: randomization

**Figure 2**: schematic overview

### Main study parameters/endpoints:

Primary endpoints: Safety will be measured by SAEs, SUSARS, and treatment related complications leading to delay in surgery (that according to the study timelines should be performed in week 5-6). Pre-operative treatment-related complications include all immune-related adverse events, attributable to the study medication, that lead to delay in surgery, with colitis being the most important, or delays due to the treatment of immunotherapy related toxicity. Logistical reasons or non-study-medication related complications (i.e. bacterial infections) leading to delay in surgery will not be considered dose-limiting toxicity. Post-operative complications including, but not limited to anastomotic dehiscence, wound dehiscence, abscess, perforation, bleeding and infection will be recorded.

Feasibility will be measured by adherence to the timelines in the study protocol.

For the run-in period, post-operative follow-up will be recorded and post-operative complications will be studied in relation to the study medication. The last time-point will be the end of study treatment, this being 3 weeks after surgery.

To meet this endpoint, all patients will be discussed in our immunotherapy team meeting (consisting of at least a medical oncologist and gastro-intestinal surgeon) the week before surgery, to evaluate whether immunotherapy related toxicity or treatment of immunotherapy-related toxicity should lead to delay in surgery.

Secondary endpoints:

- Primary readout will be the effect of therapy on intratumoral T-cell infiltration, CD4/CD8 ratio and immune checkpoints upregulation in the time interval pre- and post-treatment in biopsies.
- Immunogenic mutational load will be determined by tumor tissue DNA WES. Peripheral blood DNA WES will also be performed and used as a control for somatic mutation sorting (only genes relating to colon cancer and/or immune-related genes, deemed informational for this study, will be assessed).
- Immune suppressive pathways, IFN-y induced gene expression and COX2 induced gene expression changes will be analyzed by use of RNA sequencing on pre- and post-therapy tissue;
- Date of relapse, as determined by disease recurrence or disease-related death during follow-up after surgery. Follow-up will be performed according to local and/or national guidelines;
- The immunoscore will be determined using information obtained from analyses for the primary endpoint, using quantification of cytotoxic and memory T cells in the core of the tumor (CT) and in the tumor's invasive margin (IM) (**Appendix E)**;
- Organoids cultured from both normal and tumor tissue pre- and post-therapy using biopsies obtained by colonoscopy and at resection for a selected group of patients based on findings within the TME and availability of sufficient material;

**Nature and extent of the burden and risks associated with participation, benefit and group relatedness:**

Currently, no pre-operative treatment is given to patients with colon cancer. Post-operative adjuvant chemotherapy is commonly administered to patients with stage 3 tumors and in some cases in stage 2 tumors, and has been shown to marginally increase PFS and OS, the latter by approximately 5%.

Patients within this trial are exposed to immunotherapeutic drugs, which may lead to immune related adverse events. We expect the limited exposure to these drugs within this study to carry a low risk of adverse events and no significant delays in surgical resection of the primary tumor. Furthermore, surgical interventions in patients on immunotherapeutic regimens does not seem to increase the risk of complications.

Possible benefit to this short-term treatment is not yet known.

Previous reports of clinical studies using combinations of ipilimumab and nivolumab show grade 3-4 adverse events in 44-58% of patients, with 37% of treatments-related events leading to discontinuation of treatment. Concerning safety and toxicity of the proposed combination of ipilimumab 1mg/kg once only and nivolumab 3mg/kg administered twice, data from the Checkmate-012 trial were extrapolated. In this study, several schemes of

combination treatment were applied, with the closest one to our proposed scheme being ipilimumab 1mg/kg every 12 weeks and nivolumab 3 mg/kg every two weeks. In this cohort of the study, treatment related adverse events were observed in 74% of subjects, with the most common being skin toxicity (39%), gastro-intestinal (18%), endocrine (8%), renal (8%) and hypersensitivity/infusion reactions (5%).

Grade 3 or grade 4 treatment related events were noted in 29% of subjects, with the most common events being gastro-intestinal (5%), renal (5%), endocrine, pulmonary (pneumonitis) and skin (3% each). There were no treatment-related deaths. Patients in this specific cohort received a median of 13 nivolumab doses and 3 ipilimumab doses.[12]

These findings show a favorable toxicity profile for the alternatively dosed combination of nivolumab and ipilimumab, with a low frequency of grade 3 and 4 treatment related events. Also, considering the fact that patients in this study will only receive one single low dose of ipilimumab and two cycles of nivolumab, the incidence of treatment related events might be lower than abovementioned.

***See <u>paragraph 10</u> for a comprehensive risk-benefit analysis.***

# 1. INTRODUCTION AND RATIONALE

## Background colorectal cancer

Colorectal cancer is the fourth most common type of cancer and the second leading cause of cancer-related deaths. In the Netherlands only, approximately 31.000 cases of colorectal cancer are diagnosed annually and 10.000 patients die each year due to the consequences of this disease.

Approximately 80 percent of cancers are localized to the colon wall and/or regional nodes and surgical resection is the only curative treatment. However, outcome is related to both disease extent at presentation (i.e., TNM stage), and tumor biology and microenvironment. Approximately 30%–50% of colon cancer patients will relapse and die of their disease. Because early cancer produces no symptoms and because many of the symptoms are non-specific, aggressive efforts for early detection are taking place.

Currently, no pre-operative treatment is given to patients with non-metastatic colon cancer. Post-operative adjuvant therapy is aimed at reducing the risk of relapse and death and has shown the most benefit for node-positive, stage III disease, whereas benefit in stage II disease remains controversial. Adjuvant chemotherapy in stage 2 tumors is considered in patients with high-risk tumors, as defined by T4 tumor, <10 lymph nodes, perforation or obstruction at presentation, vascular invasion or undifferentiated tumors. Adjuvant treatment using doublet therapy of 5-FU/folinic acid and oxaliplatin has been shown to marginally increase PFS and OS. The increase in 5-year OS is approximately 5%.[10,11,13,14]

Five-year survival after surgical resection alone is: for stage I 85-95%, stage II 60-80% and stage III 30-60%. These wide ranges in survival reflect major differences in prognosis upon stage subset, tumor grading and other biological characteristics. Tumors with microsatellite instability have higher immune cell infiltration and also, in the non-metastatic setting, have a significantly better prognosis irrespective of tumor stage. Recent data has substantially increased interest in immune cell infiltration in colorectal cancers as an indicator for prognosis.[1,3,6,15]

Furthermore, preclinical work suggests that COX2-inhibition could help to circumvent tumor microenvironment-driven evasion of immunotherapy.[8,9]

## Rationale for Immunoscore

As early as 2005, Pages et al published results showing that immune cell infiltration in colorectal cancer was an independent prognostic factor for overall survival. Absence of these T-cells, along with higher TNM stage, was independently associated with worse overall and disease-free survival.[3]

Ensuing evidence strengthened the notion that immune cell infiltration, and particularly the

Immunoscore, is a better predictor of prognosis than microsatellite status. 'Immunoscore'' is a scoring system, based on the quantification of cytotoxic and memory T cells in the core of the tumor (CT) and in the tumor's invasive margin (IM). Recently, Mlecnik et al[15] showed that even though MSI tumors are more often infiltrated with mutation specific cytotoxic T cells and have high immunoscores, a small subset of MSS tumors also exhibited this phenotype and have similar survival curves. Surprisingly, low immunoscore in a subset of MSI tumors seemed to correlate with worse overall and disease-specific survival. These findings, along with results from mainly melanoma studies showing the importance of T-cell infiltration in the response to checkpoint inhibition, suggest that using the Immunoscore might help guide immunotherapy strategies.

### Rationale for COX2 inhibition

Even though most tumors arise in immunocompetent hosts, mechanisms developed by the tumor lead to evasion of the immune system by means of immunoediting.[14] This reduced tumor immunogenicity can be a consequence of T cell blockade by upregulation of immune checkpoints, recruitment of suppressive cells, production of immunosuppressive factors and/or down regulation of antigen-presenting MHC molecules.[16]

Inflammatory cytokines also play an important role in the immunogenicity of tumors and can be produced by the stroma, TILs or by the cancer cells themselves. Of these cytokines, prostaglandin E2 (PGE2) plays a key role, and is associated with cancer cell survival, growth, migration, invasion, angiogenesis and immunosuppression.[17] Cyclooxygenase-1 and 2, enzymes that are essential in the production of PGE2, are often overexpressed in many cancer types including colorectal cancer.

In recent publications, type 1 immunity was shown to induce COX2 activation and PGE2 synthesis, which in turn leads to recruitment and induction of MDSCs and inhibition of dendritic cells.[9,18,19] MDSCs are presumed to inhibit both innate and adaptive immune responses.[20,21] COX2 inhibition in preclinical models lead to reversion of CD8+ T-cell and suppression and, when combined with anti-PD1, led to significantly improved responses to therapy.[8,22] This together with recent publications of a meta-analysis, where aspirin use after diagnosis was inversely associated with CRC overall mortality particularly among patients with positive PTGS2 (COX2) expression, and an observational study showing the efficacy of aspirin as secondary prevention, is reason to investigate whether the immunogenicity of CRCs and response to immunotherapy can be improved by the addition of COX inhibitors, especially in MSS tumors with low immunoscores.[23,24] These findings suggest that COX inhibitors could be useful adjuvants for immune-based therapies, enhancing response to immunotherapy by reducing MDSCs in the TME.[25]

**Rationale for combining ipilimumab + nivolumab**

Nivolumab and ipilimumab enhance T-cell antitumor activity by non-redundant, complementary mechanisms.[25-27] Preclinical data suggest synergy with dual CTLA-4 and PD-1 blockade compared to either agent alone by increased proliferation of effector CD8+ and CD4+ T cells and decreased intratumoral regulatory T cells.[28] Anti-CTLA-4 can lead to enhanced priming and activation of antigen-specific T cells and potentially clearance of regulatory T cells from the tumor microenvironment. Clinical experiences with nivolumab plus ipilimumab demonstrate deep and durable responses in previously treated melanoma and lung cancer.[29] Combination therapy seemed to most dramatically benefit patients who were less likely to benefit from PD-L1 or PD-1 inhibition alone, because their tumors were PDL-1 negative.[30]

The combination has proven synergistic especially in patients with PD-L1 negative tumors, whereas PD-L1 positive tumors have similar results with single agent anti-PD1 (nivolumab) when compared to the combination (nivolumab and ipilimumab).[31] This suggests an immunogenic activation by anti-CTLA-4 agents, which in turn facilitates the cytotoxic effect of anti-PD1/PDL1, in those patients with a meager lymphocyte infiltration and immune regulatory factors.

Previous reports of clinical studies using combinations of ipilimumab and nivolumab show grade 3-4 adverse events in 44-58% of patients, with 37% of treatments-related events leading to discontinuation of treatment. Concerning safety and toxicity of the proposed combination of ipilimumab 1mg/kg once only and nivolumab 3mg/kg administered twice, data from the Checkmate-012 trial were extrapolated. In this study, several schemes of combination treatment were applied, with the closest ones to our proposed scheme being ipilimumab 1mg/kg every 6 or 12 weeks and nivolumab 3 mg/kg every two weeks. In this cohort of the study, treatment related adverse events were observed in 74% of subjects, with the most common being skin toxicity (39%), gastro-intestinal (18%), endocrine (8%), renal (8%) and hypersensitivity/infusion reactions (5%).

Grade 3 or grade 4 treatment related events were noted in 29% of subjects, with the most common events being gastro-intestinal (5%), renal (5%), endocrine, pulmonary (pneumonitis) and skin (3% each). There were no treatment-related deaths. Patients in this specific cohort received a median of 13 nivolumab doses and 3 ipilimumab doses.[12] Additional safety data from the Checkmate-012 trial for the combination was reported in patients with advanced MSI mCRC, demonstrating grade 3-4 treatment-related events in 27% of subjects. The most

common any grade event was diarrhea of grade 2 or less, occurring in 43% of patients. In this cohort, no grade 3-4 diarrhea was reported.[5]

These findings show a favorable toxicity profile for the alternatively dosed combination of nivolumab and ipilimumab, with a low frequency of grade 3 and 4 treatment related events. Also, considering the fact that patients in this study will only receive one single low dose of ipilimumab and two cycles of nivolumab, the incidence of treatment related events might be lower than abovementioned.

**Rationale for pre-operative treatment**

In colorectal cancer, Galon et al showed that the percentage of immunoscore high tumors is significantly lower in the metastatic disease setting as compared to localized tumors.
This study offers a unique window of opportunity for collection of pre-treatment biopsies and tumor material at resection for analysis of the TME, which will be essential in understanding colon cancer immunogenicity and evasion mechanisms prior to and after checkpoint inhibition.

**Rationale for organoids**

Recent advances from the cancer stem-cell field now allow the *in vitro* culture and expansion of “tumor organoids”, three-dimensional cultures of cancer stem cells that can be propagated indefinitely, but most importantly on an individual patient basis.[32] However, the immunogenicity of tumor organoids is still unknown and to address this question, we aim to evaluate whether autologous tumor organoids are potent inducers of a T-cell response *in vitro*. If autologous tumor organoids prove to be immunogenic, they may contribute to the optimization of current immunotherapeutic strategies such as patient specific anti-tumor vaccines or the adoptive transfer of T cells primed *in vitro* with autologous tumor organoids. In this study, the in vitro sensitivity of organoids generated from pre-treatment biopsies and from post-treatment resection material will be compared in their sensitivity to T cell pressure (in the presence or absence of anti-PD1). Moreover, depending on findings within the TME, knockdown and overexpression experiments in pre- and post-treatment tumor organoids could evaluate the relevance of these findings for sensitivity to T cell attack.

## 2. OBJECTIVES

Primary Objective: To determine the safety and feasibility of pre-operative immunotherapy in CRC.

Secondary Objectives:

- To explore the immune activating capacity of short-term pre-operative immunotherapy and COX-2 inhibition in primary MSI and MSS colon cancers (CRCs) and identify underlying potential mechanisms of escape from the immune system.
  - To assess the immunogenic mutational load by DNA WES and correlation with putative markers of response;
  - To assess changes in immune suppressive pathways, IFNy induced gene expression by RNA sequencing;
- To determine pathological tumor changes following short-term immunotherapy;
- To assess relapse free survival;
  - To explore the Immunoscore as a predictor of immunotherapy induced changes in the TME and correlation to putative markers of response;
  - To explore the immunogenicity and in vitro T-cell sensitivity of tumor organoid cultures;

Translational objectives:

- To analyse expression and changes in expression of CD3, CD4, CD8, CD45RO, tumor infiltrating lymphocytes (TILs) in stroma and tumor, FOXP3+ regulatory T cells, CD68/CD163 (MDSCs), M2 macrophages, PD-L1 in pre-treatment and resection tumor material;
- To analyse gain/change in presence of other biomarkers and immune checkpoints, which could include LAG-3, GITR, TIM-3, TBET, IDO, B-catenine, PD-1, CDX2, COX2, CEA, TBET, CD4/CD8+ T cell ratio (these analyses will depend on the amount of material available for comparison);
- To assess changes in immune suppressive pathway and inflammation signatures, IFNy induced gene expression and the added effects of COX2-i when combined with nivolumab and ipilimumab (e.g. up-regulation of IFNy, TNF, IL12 and downregulation of IL6, IL8, CXCL1, and G-CSF);
- To analyse clonality and changes in the TCR repertoire pre- and post-therapy in the subset of patients showing significant changes in the TME;
- To identify subtypes of MSS CRC with early signs of response and correlation with the Immunoscore and consensus molecular subtypes (CMS);

- To assess immunogenic mutational load and neoantigen load by DNA WES and RNA sequencing;
- To identify and explore the predictive value of checkpoint expression, cytokines and chemokines, CRP/ESR/LDH/CEA;
- To explore the effect of COX2-i on the TME and response predictors to immunotherapy in MSS tumors with low immunoscore versus high immunoscore;
- To evaluate and explore post-immunotherapy effects on draining lymph nodes using resection material;
- To explore the immunogenicity of autologous tumor organoids and their T cell reactivity in vitro, using normal tissue organoids as controls;
- To explore the phenomenon of exhausted T cells (Tex) by assessing IHC and gene expression markers, including but not limited to TBET, Eomes and PD-1;
- To assess ctDNA before and during therapy and after resection of the primary tumor;
- In a select group, to evaluate the correlation of gene profiles derived from tumor educated platelets with changes in tumor gene expression in the tumor;
- To explore changes in inflammation signatures, immune checkpoints, immune suppressive pathways and IFN$\gamma$ induced gene expression by using RNA sequencing.

## 3. STUDY DESIGN

In this single-center, open-label, exploratory study, we will enroll 60 patients within two years, including 30 patients with MSS tumors and 30 patients with MSI tumors. Patients with MSS tumors will be randomized to either group 1 or 2 (figure 1). Since the incidence of MSS tumors is higher, accrual for this group is expected to be faster than for the MSI group. After inclusion of 30 patients with MSS tumors, this cohort will be closed, after which only patients with MSI tumors will be included.

During a run-in period, three patients, regardless of microsatellite status, will be included and treated with nivolumab monotherapy. Safety and feasibility will be evaluated and reported to the medical ethical committee. When this pre-operative treatment is deemed safe and feasible, as defined in the primary endpoint, accrual for the combination treatment as described below will be started. In case of immunotherapy related toxicity leading to delay in surgery or study-medication attributable post-operative complications in one or more of the first three patients, another 3 patients will be included in the same cohort and evaluated in the same way. If no study-medication related toxicity is seen in the first 3 patients treated with nivolumab monotherapy, accrual for the combination treatment cohorts will be started. As for the first cohort, safety evaluation and reporting to the medical ethical committee will be done for the first 3 patients in the combination treatment cohorts. After safety evaluation for the combination treatment, accrual according to this protocol will be continued. Delay in surgery due to logistical problems or other non-study-medication related co-morbiditywill not be considered dose-limiting toxicity.

Group 1 will be treated with a single dose of ipilimumab 1mg/kg on day one and two cycles of nivolumab 3mg/kg on day one and 15, respectively. Group 2 will be treated with 200mg once daily celecoxib from day 1 until the day prior to surgery, a single dose of ipilimumab 1mg/kg on day one and two cycles of nivolumab 3mg/kg one day one and 15.

Patients with MSI tumors will all be allocated to group 1. Observations made in this cohort will mainly serve as positive comparators of a productive anti-tumor immune response. Findings within the TME of MSI tumors will be the main source of information on the read-outs to be used for the TME of MSS tumors. The Immunoscore, using the scoring system by Galon et al[1], will be performed on all tumors using biopsy material. Based on recent findings, we expect 30-40% of MSS and 60-70% of MSI primary colon tumors to have high Immunoscores. Feasibility of the Immunoscore on tumor biopsies will be tested in a separate cohort. If the Immunoscore cannot be determined based on primary tumor biopsies, inclusion of patients with MSI tumors will be limited to 20 total.

Patients will be treated with short-term immunotherapy ± COX2-inhibitors. This treatment will

be given during the window period until surgical resection of the tumor. Surgery will be planned 4 – 5 weeks after inclusion in the study, with a maximum of 6 weeks after registration in the study (figure 2).
After completion of accrual, analysis of the primary endpoint will be performed. Based on these findings and available compounds at that time, the study will be amended to include new drug combinations with one or more of the study drugs used in the first group of patients.

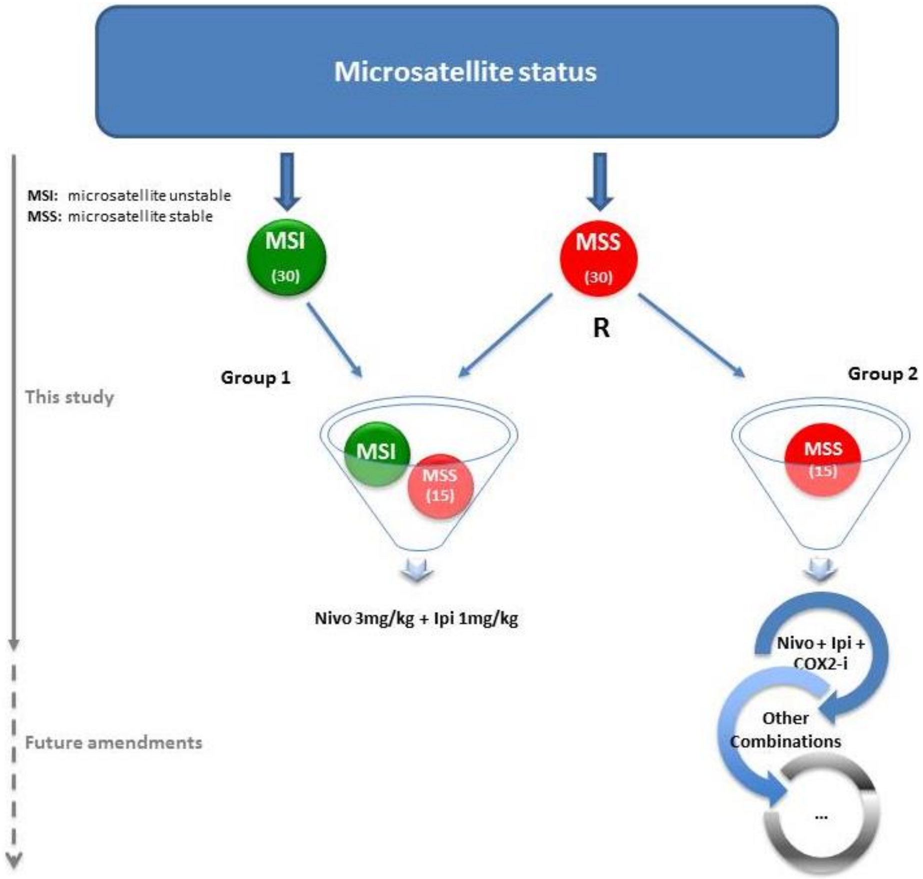


**Figure 1: schematic overview of randomization in NICHE study.**

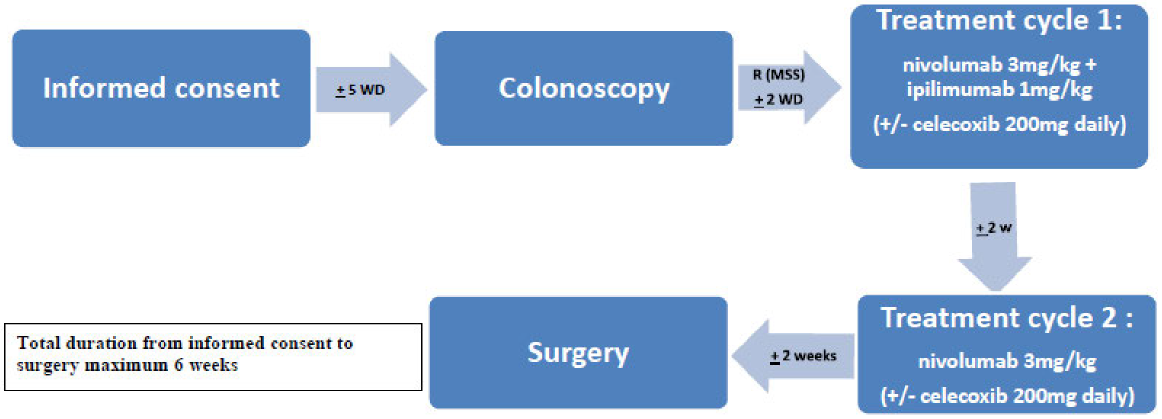


**Figure 2: schematic overview of NICHE study.**

# 4. STUDY POPULATION

## 4.1. Population (base)

**Patient selection criteria:** In order to be eligible to participate in this study, a subject must meet all of the following criteria:

- Signed written informed consent;
- Patients at least 18 years of age;
- Stage 1-3 adenocarcinoma of the colon (and upper rectum/rectosigmoid considered as non-rectal and not undergoing neoadjuvant treatment);
- No signs of distant metastases on CT-scan and physical examination;
- No signs of obstruction or macroscopic bleeding;
- No clinical symptoms or radiological suspicion of perforation;
- Colonoscopy must be performed after registration to obtain study-specific biopsies. If biopsies are not possible, patients cannot be included in the study;
- WHO performance status of 0 or 1;
- Screening laboratory tests must meet the following criteria and should be obtained within 7 days prior to randomization/registration: WBC $\geq$ 2.0 x 10^9/L, ANC $\geq$ 1.5x10^9/L, platelets $\geq$ 100 x 10^9/L, Hemoglobin $\geq$ 5.0mmol/L. Transfusion is allowed to obtain an adequate hemoglobin level. Liver function tests: total bilirubin < 1.5 upper limit of normal (ULN) (except for subjects with Gilbert syndrome, who can have total bilirubin <3.0 mg/dL); alkaline phosphatase <2.5 ULN; transaminases (ASAT/ALAT) <3 x ULN; LDH < 2 x ULN;
- Creatinine clearance (Cockcroft-Gault) of $\geq$40 ml/min;
- Women of childbearing potential (WOCBP)* must use appropriate method(s) of contraception. WOCBP should use an adequate method to avoid pregnancy for 23 weeks (30 days plus the time required for nivolumab to undergo five half-lives after the last dose of investigational drug;
- Women of childbearing potential must have a negative serum or urine pregnancy test (minimum sensitivity 25 IU/L or equivalent units of HCG) within 24 hours prior to the start of nivolumab;
- Men who are sexually active with WOCBP must use any contraceptive method with a failure rate of less than 1% per year. Men receiving nivolumab and who are sexually active with WOCBP will be instructed to adhere to contraception for a period of 31 weeks after the last dose of investigational product Women who are not of childbearing potential (i.e., who are postmenopausal or surgically sterile as well as azoospermic men do not require contraception;

- CT-scan must be performed within 28 days prior to registration;
- No previous treatment with immune checkpoint inhibitors targeting CTLA-4, PD-1 or PD-L1;
- For patients with MSS tumors: no current use of NSAIDs or COX2-inhibitors at registration and no active peptic ulcer, gastrointestinal bleeding, unstable ischemic heart disease of thrombus etiology or significant established ischemic heart disease, peripheral arterial disease and/or cerebrovascular disease;
- No radiotherapy prior to or planned post-surgery radiotherapy;
- Allergies and Adverse Drug Reaction
  - No history of allergy to study drug components
  - No history of severe hypersensitivity reaction to any monoclonal antibody
  - No history of allergy or severe hypersensitivity to NSAIDs or COX2-I (MSS tumors)
- No intercurrent illnesses, including but not limited to infections, unstable angina pectoris
- No underlying medical conditions that, in the Investigator's opinion, will make the administration of the study drug hazardous or obscure the interpretation of toxicity determination of adverse events;
- No positive test for hepatitis B virus surface antigen (HBsAg) or hepatitis C virus ribonucleic acid (HCV antibody) indicating acute or chronic infection;
- No history of testing positive for human immunodeficiency virus (HIV) or known acquired immunodeficiency syndrome (AIDS);
- No active autoimmune disease or a documented history of autoimmune disease, or other medical conditions requiring systemic steroid or immunosuppressive medications, except for subjects with vitiligo, diabetes mellitus type 1, residual hypothyroidism due to autoimmune condition only requiring hormone replacement, psoriasis or resolved childhood asthma/atopy not requiring systemic treatment;
- No conditions requiring systemic treatment with either corticosteroids (> 10 mg daily prednisone equivalents) or other immunosuppressive medications within 14 days of study drug administration. Inhaled or topical steroids and adrenal replacement doses > 10 mg daily prednisone equivalents are permitted in the absence of active autoimmune disease;
- No live vaccines in the 4 weeks prior to inclusion;
- No history of uncontrolled medical or psychiatric illness;
- No psychological, familial, sociological or geographical condition potentially hampering compliance with the study protocol and follow-up schedule;

- No current pregnancy or breastfeeding;
- No malignancies other than disease under study within 5 years prior to inclusion.

| |
|---|
| * Women of childbearing potential" is defined as any female who has experienced menarche and who has not undergone surgical sterilization (hysterectomy or bilateral oophorectomy) or who is not postmenopausal. Menopause is defined clinically as 12 months of amenorrhea in a woman over 45 in the absence of other biological or physiological causes. In addition, women under the age of 55 must have a documented serum follicle stimulating hormone (FSH) level less than 40 mIU/mL. |

### 4.2. Sample size calculation

For this exploratory study no formal sample size calculation was performed. A total of 60 patients will be recruited, 30 per study arm.

This guarantees enough and timely accrual and limits the number of patients treated, since this short-term treatment has no known benefit or efficacy in patients with colon cancer. Furthermore, if no effects (defined as findings within the TME, see endpoints) are seen in a certain subgroup (e.g. MSS) and/or the primary endpoint is not met (safety), the allocated treatment is not likely to be explored further.

# 5. TREATMENT OF SUBJECTS

## 5.1. Investigational product/treatment

Patients in **group 1** will be treated with a single dose of ipilimumab 1mg/kg on day one and two cycles of nivolumab 3mg/kg on day one and 15, respectively.
Patients in **group 2** will be treated with celecoxib 200mg once daily from day 1 until the day prior to surgery, a single dose of ipilimumab 1mg/kg on day one and two cycles of nivolumab 3mg/kg one day one and 15.

## 5.2. Prohibited medications

1) Concurrent chemotherapy, hormonal therapy, immunotherapy regimes or radiation therapy, standard or investigational.
2) Use of growth factors including, but not limited to, granulocyte colony stimulating factor (G-CSF), granulocyte macrophage colony stimulating factor (GM-CSF), or erythropoietin stimulating agents are not permitted unless deemed absolutely necessary by investigator, after consultation with medical monitor.
3) Use of systemic corticosteroids at >10mg daily prednisone equivalent, unless required for the treatment of infusion reactions, other adverse events, or for palliation as determined by the investigator.
4) Steroids must not be given as prophylactic anti-emetic therapy.
5) Use of herbal remedies is not permitted.
6) For group 1: use of NSAID’s and/or selective COX1/2 inhibitors is not permitted.

| **Permitted Therapy** |
|---|
| Subjects are permitted to use topical, ocular, intra-articular, intranasal, and inhalational corticosteroids (with minimal systemic absorption). Physiologic replacement doses of systemic corticosteroids are permitted, even if > 10 mg/day prednisone equivalents. A brief course of corticosteroids for prophylaxis (eg, contrast dye allergy) or for treatment of non-autoimmune conditions (eg, delayed-type hypersensitivity reaction caused by contact allergen) is permitted. |

## 5.3. Escape medication

Immuno-oncology (I-O) agents are associated with adverse events that can differ in severity and duration than adverse events caused by other therapeutic classes. Nivolumab and ipilimumab are considered immuno-oncology agents in this protocol. Management algorithms have been developed to assist in assessing and managing the following groups of adverse events: gastrointestinal, renal, pulmonary, hepatic, endocrinopathies, skin, and neurological.

**See appendix C** for treatment algorithms. Suspicion of immune related **colitis** requiring corticosteroid therapy shall be discussed with the study coordinator for assessment of the need for steroid therapy. **Infliximab** may be prioritized over corticosteroids if surgery is imminent to reduce chances of post-operative complications.
Early recognition and intervention are recommended according to the management algorithms; and in addition include ophthalmologic evaluations for any visual symptoms in order to evaluate for nivolumab or ipilimumab related uveitis.
In patients with a history of ulcer, concurrent use of anticoagulants, age >65 years and/or corticosteroids for the treatment of study drug related complications, proton pump inhibitors (PPI) should be started. Patients in group 1 receiving corticosteroids for immunotherapy related adverse events, PPI should also receive concomitant PPIs. Patients with dyspepsia and other celecoxib related symptoms should be given PPI to relieve symptoms.

**Dose Delay Criteria**

Because of the potential for clinically meaningful nivolumab and ipilimumab-related AEs requiring early recognition and prompt intervention, management algorithms have been developed for suspected AEs of selected categories (appendix C).
Dose delay criteria apply for all drug-related adverse events (regardless of whether or not the event is attributed to nivolumab, ipilimumab, or both). All study drugs must be delayed until treatment can resume.
Nivolumab and ipilimumab administration should be delayed for the following:

- Any Grade ≥ 2 non-skin, drug-related adverse event, with the following exceptions:
  - Grade 2 drug-related fatigue or laboratory abnormalities do not require a treatment delay
- Any Grade 3 skin, drug-related adverse event
- Any Grade 3 drug-related laboratory abnormality, with the following exceptions for asymptomatic amylase or lipase, AST, ALT, or total bilirubin:
  - Grade 3 amylase or lipase abnormalities that are not associated with symptoms or clinical manifestations of pancreatitis do not require a dose delay. It is recommended to consult with the principle investigator for Grade 3 amylase or lipase abnormalities.
  - If a subject has a baseline AST, ALT, or total bilirubin that is within normal limits, delay dosing for drug-related Grade ≥ 2 toxicity
  - If a subject has baseline AST, ALT, or total bilirubin within the Grade 1 toxicity range, delay dosing for drug-related Grade ≥ 3 toxicity

- Any adverse event, laboratory abnormality, or intercurrent illness which, in the judgment of the investigator, warrants delaying the dose of study medication.

Celecoxib administration should be delayed for the following:

- Unstable ischemic heart disease, congestive heart failure and/or cerebrovascular disease;
- Gastrointestinal (GI) bleeding;
- Decrease in renal function, with estimated creatinine clearance <30 mL/min;
- Grade 3 drug-related liver function test (LFT) abnormality.

**Criteria to Resume Treatment**

Subjects may resume treatment with study drug when the drug-related AE(s) resolve to Grade ≤1 or baseline value within one week, with the following exceptions:

- Subjects may resume treatment in the presence of Grade 2 fatigue
- Subjects who have not experienced a Grade 3 drug-related skin AE may resume treatment in the presence of Grade 2 skin toxicity
- Subjects with baseline Grade 1 AST/ALT or total bilirubin who require dose delays for reasons other than a 2-grade shift in AST/ALT or total bilirubin may resume treatment in the presence of Grade 2 AST/ALT OR total bilirubin
- Subjects with combined Grade 2 AST/ALT AND total bilirubin values meeting discontinuation parameters should have treatment permanently discontinued
- Drug-related pulmonary toxicity, diarrhea, or colitis, must have resolved to baseline before treatment is resumed
- Drug-related endocrinopathies adequately controlled with only physiologic hormone replacement may resume treatment.

If the criteria to resume treatment are met, the subject should restart treatment at the next scheduled time point per protocol. However, if the treatment is delayed past the next scheduled time point per protocol, the next scheduled time point will be delayed until dosing resumes.

If treatment is delayed > 1 week, the subject must be permanently discontinued from study therapy, to avoid further delay of surgery.

**Discontinuation Criteria**

Treatment should be permanently discontinued for the following:

- Any Grade 2 drug-related uveitis or eye pain or blurred vision that does not respond to topical therapy and does not improve to Grade 1 severity within the re-treatment period OR requires systemic treatment
- Any Grade 3 non-skin, drug-related adverse event lasting > 7 days, with the following exceptions for drug-related laboratory abnormalities, uveitis, pneumonitis, bronchospasm, diarrhea, colitis, neurologic adverse event, hypersensitivity reactions, and infusion reactions
  - Grade 3 drug-related uveitis, pneumonitis, bronchospasm, diarrhea, colitis, neurologic adverse event, hypersensitivity reaction, or infusion reaction of any duration requires discontinuation
  - Grade 3 drug-related laboratory abnormalities do not require treatment discontinuation except those noted below
    - Grade 3 drug-related thrombocytopenia > 7 days or associated with bleeding requires discontinuation
    - Any drug-related liver function test (LFT) abnormality that meets the following criteria require discontinuation:
      - AST or ALT > 8 x ULN
      - Total bilirubin > 5 x ULN
      - Concurrent AST or ALT > 3 x ULN and total bilirubin > 2 x ULN
- Any Grade 4 drug-related adverse event or laboratory abnormality, except for the following events which do not require discontinuation:
  - Isolated Grade 4 amylase or lipase abnormalities that are not associated with symptoms or clinical manifestations of pancreatitis and decrease to < Grade 4 within 1 week of onset.
  - Isolated Grade 4 electrolyte imbalances/abnormalities that are not associated with clinical sequelae and are corrected with supplementation/appropriate management within 72 hours of their onset
- Any dosing interruption lasting > 1 weeks
- Any adverse event, laboratory abnormality, or intercurrent illness which, in the judgment of the Investigator, presents a substantial clinical risk to the subject with continued nivolumab or ipilimumab dosing.

**Treatment of Nivolumab or Ipilimumab Related Infusion Reactions**

Since nivolumab and ipilimumab contain only human immunoglobulin protein sequences, it is unlikely to be immunogenic and induce infusion or hypersensitivity reactions. However, if

such a reaction were to occur, it might manifest with fever, chills, rigors, headache, rash, pruritis, arthralgias, hypo- or hypertension, bronchospasm, or other symptoms.

All Grade 3 or 4 infusion reactions should be reported as an SAE if criteria are met. Infusion reactions should be graded according to NCI CTCAE version 4.0.

Treatment recommendations are provided below and may be modified based on local treatment standards and guidelines as appropriate:

**For Grade 1 symptoms:** (Mild reaction; infusion interruption not indicated; intervention not indicated)

Remain at bedside and monitor subject until recovery from symptoms. The following prophylactic premedications are recommended for future infusions: diphenhydramine 50 mg (or equivalent) and/or paracetamol 325 to 1000 mg (acetaminophen) at least 30 minutes before additional nivolumab administrations.

**For Grade 2 symptoms**: (Moderate reaction requires therapy or infusion interruption but responds promptly to symptomatic treatment [eg, antihistamines, non-steroidal anti-inflammatory drugs, narcotics, corticosteroids, bronchodilators, IV fluids]; prophylactic medications indicated for 24 hours).

Stop the nivolumab or ipilimumab infusion, begin an IV infusion of normal saline, and treat the subject with diphenhydramine 50 mg IV (or equivalent) and/or paracetamol 325 to 1000 mg (acetaminophen); remain at bedside and monitor subject until resolution of symptoms. Corticosteroid or bronchodilator therapy may also be administered as appropriate. If the infusion is interrupted, then restart the infusion at 50% of the original infusion rate when symptoms resolve; if no further complications ensue after 30 minutes, the rate may be increased to 100% of the original infusion rate. Monitor subject closely. If symptoms recur then no further nivolumab or ipilimumab will be administered at that visit. Administer diphenhydramine 50 mg IV, and remain at bedside and monitor the subject until resolution of symptoms. The amount of study drug infused must be recorded on the electronic case report form (eCRF). The following prophylactic premedications are recommended for future infusions: diphenhydramine 50 mg (or equivalent) and/or paracetamol 325 to 1000 mg (acetaminophen) should be administered at least 30 minutes before additional nivolumab or ipilimumab administrations. If necessary, corticosteroids (recommended dose: up to 25 mg of IV hydrocortisone or equivalent) may be used.

**For Grade 3 or Grade 4 symptoms:** (Severe reaction, Grade 3: prolonged [ie, not rapidly responsive to symptomatic medication and/or brief interruption of infusion]; recurrence of symptoms following initial improvement; hospitalization indicated for other clinical sequelae [eg, renal impairment, pulmonary infiltrates]). Grade 4: (life threatening; pressor or ventilatory support indicated).

Immediately discontinue infusion of nivolumab or ipilimumab. Begin an IV infusion of normal saline, and treat the subject as follows. Recommend bronchodilators, epinephrine 0.2 to 1 mg of a 1:1,000 solution for subcutaneous administration or 0.1 to 0.25 mg of a 1:10,000 solution injected slowly for IV administration, and/or diphenhydramine 50 mg IV with methylprednisolone 100 mg IV (or equivalent), as needed. Subject should be monitored until the investigator is comfortable that the symptoms will not recur. Nivolumab or ipilimumab will be permanently discontinued. Investigators should follow their institutional guidelines for the treatment of anaphylaxis. Remain at bedside and monitor subject until recovery from symptoms. In the case of late-occurring hypersensitivity symptoms (eg, appearance of a localized or generalized pruritis within 1 week after treatment), symptomatic treatment may be given (eg, oral antihistamine, or corticosteroids).

**Treatment of Nivolumab or Ipilimumab Related Colitis (see appendix C)**

Suspicion of immune related colitis requiring corticosteroid therapy must be discussed with the study coordinator/PI for assessment of the need for steroid therapy. Infliximab may be prioritized over corticosteroids if surgery is imminent to reduce chances of post-operative complications.

# 6. INVESTIGATIONAL PRODUCT

## 6.1. Name and description of investigational product(s)

**Nivolumab:** 3 mg/kg, day 1 and 15, two cycles in total.

Nivolumab (also referred to as BMS-936558 or MDX1106) is a human monoclonal antibody (HuMAb; immunoglobulin G4 [IgG4]-S228P) that targets the programmed death-1 (PD-1) cluster of differentiation 279 (CD279) cell surface membrane receptor. PD-1 is a negative regulatory molecule expressed by activated T and B lymphocytes. Binding of PD-1 to its ligands, programmed death–ligands 1 (PD-L1) and 2 (PD-L2), results in the down-regulation of lymphocyte activation. Inhibition of the interaction between PD-1 and its ligands promotes immune responses and antigen-specific T-cell responses to both foreign antigens as well as self-antigens. Nivolumab is expressed in Chinese hamster ovary (CHO) cells and is produced using standard mammalian cell cultivation and chromatographic purification technologies. The clinical study product is a sterile solution for parenteral administration.

***For more information, see the Nivolumab IB.***

**Ipilimumab:** 1mg/kg, on day 1, one cycle in total.

Ipilimumab (BMS-734016, MDX010, MDX-CTLA4) is a fully human monoclonal IgG1κ that binds to the CTLA-4 antigen expressed on a subset of T cells from human and nonhuman primates. CTLA-4 is a negative regulator of T-cell activity. Ipilimumab is a mAb that binds to CTLA-4 and blocks the interaction of CTLA-4 with its ligands, CD80/CD86. Blockade of CTLA-4 has been shown to augment T-cell activation and proliferation, including the activation and proliferation of tumor infiltrating T-effector cells. Inhibition of CTLA-4 signaling can also reduce T-reg function, which may contribute to a general increase in T-cell responsiveness, including the anti-tumor response. Ipilimumab is currently under development for the treatment of subjects with cancer. Studies are being sponsored by BMS, with the US National Cancer Institute (NCI) and Mayo Clinic as additional sponsors.

**PRODUCT INFORMATION TABLE**:

| Table | | Product Description | | | |
|---|---|---|---|---|---|
| **Product Description and Dosage Form** | **Potency** | **Primary Packaging (Volume)/ Label Type** | **Secondary Packaging (Qty) /Label Type** | **Appearance** | **Storage Conditions (per label)** |
| Nivolumab BMS-936558-01 Solution for Injection[a] | 100 mg (10 mg/mL) | 10 mL viall | 5 or10 vials per carton/ Open-label | Clear to opalescent colorless to pale yellow liquid. May contain particles | 2 to 8°C. Protect form light and freezing |
| Ipilimumab Solution for Injection | 200 mg (5 mg/mL) | 40 mL vial | 4 vials per carton/Open-label | Clear, colorless to pale yellow liquid. May contain particles | 2 to 8°C. Protect from light and freezing. |

*Nivolumab may be labeled as BMS-936558-01 Solution for Injection

**Celecoxib (Pfizer):** 200 mg daily taken once daily or in two divided doses starting on day 1, without regard to timing of meals, until one day before surgery.

Celecoxib is a cyclooxygenase-2 (COX-2) specific inhibitor, a member of a larger class of non-steroidal anti-inflammatory drugs (NSAIDs) that exhibits anti-inflammatory, analgesic, and antipyretic activities in animal models. The mechanism of action of celecoxib is believed to be due to inhibition of prostaglandin synthesis, primarily by inhibition of COX-2. At therapeutic concentrations in humans, celecoxib does not inhibit cyclooxygenase-1 (COX-1). COX-2 is induced in response to inflammatory stimuli. This leads to the synthesis and accumulation of inflammatory prostanoids, in particular prostaglandin E2, causing inflammation, edema and pain. In animal models, celecoxib acts as an anti-inflammatory, analgesic and antipyretic agent by blocking the production of inflammatory prostanoids via COX-2 inhibition. In animal colon tumor models, celecoxib reduced the incidence and multiplicity of tumors.

Contraindications

Known hypersensitivity to celecoxib or any of the excipients contained in the Celecoxib capsules. Demonstrated allergic-type reactions to sulfonamides. Celecoxib should not be given to patients who have experienced asthma, urticaria, or allergic-type reactions after taking aspirin or other NSAIDs, including other COX-2 specific inhibitors. Severe, rarely fatal, anaphylactic reactions to NSAIDs have been reported in such patients. Celecoxib should not

be used with other NSAIDs because of the absence of any evidence demonstrating synergistic benefits and the potential for additive adverse reactions; the peri-operative treatment of pain in patients undergoing coronary artery bypass graft (CABG) surgery (see precautions).

Celecoxib is contraindicated in:

- Patients with unstable ischemic heart disease of thrombus etiology or significant established ischemic heart disease, peripheral arterial disease and/or cerebrovascular disease (see precautions, cardiovascular and thrombotic events).
- Patients with active peptic ulceration or gastrointestinal (GI) bleeding.
- Patients with estimated creatinine clearance <30 mL/min.
- Patients with congestive heart failure (NYHA II-IV).
- Patients with severe hepatic impairment.

Gastrointestinal Effects

Infrequently, serious gastrointestinal toxicity such as bleeding, ulceration, and perforation of the stomach or intestine has been observed in patients treated with celecoxib. Physicians and patients should remain alert for ulceration and bleeding, even in the absence of previous GI tract symptoms.

Celecoxib exhibited a low incidence of gastroduodenal ulceration and serious clinically significant GI events within clinical trials. The following information for NSAIDs should be borne in mind. Serious GI toxicity, such as bleeding, ulceration and perforation of the stomach, small intestine or large intestine can occur at any time, with or without warning symptoms, in patients treated with NSAIDs. Minor upper GI problems, such as dyspepsia, are common, and may also occur at any time during NSAID therapy. Therefore, physicians should remain alert for ulceration and bleeding in patients treated with NSAIDs, even in the absence of previous GI tract symptoms. Patients should be informed about the signs and/or symptoms of serious GI toxicity and the steps to take if they occur. The utility of periodic laboratory monitoring has not been demonstrated, nor has it been adequately assessed. Only one in five patients who develop a serious upper GI adverse event on NSAID therapy is symptomatic. It has been demonstrated that upper GI ulcers, gross bleeding or perforation, caused by NSAIDs, appear to occur in approximately 1% of patients treated for 3-6 months, and in about 2-4% of patients treated for one year. These trends continue thus, increasing the likelihood of developing a serious GI event at some time during the course of therapy. However, even short-term therapy is not without risk. Most spontaneous reports of fatal GI events are in elderly or debilitated patients and therefore special care should be taken in treating this population.

Among 5,285 patients who received celecoxib in the original arthritis trials of 1 to 6 months duration (most were 3 month studies) at a daily dose of 200 mg or more, 2 (0.04%) experienced significant upper GI bleeding, at 14 and 22 days after initiation of dosing. Approximately 40% of these 5,285 patients were in studies that required them to be free of ulcers by endoscopy at study entry. Thus it is unclear if this study population is representative of the general population.
The incidences of complicated and symptomatic ulcers for patients treated with celecoxib 400 mg BD (4-fold and 2-fold greater than the recommended OA and RA doses, respectively) from the prospective randomized controlled long-term outcomes trial in 8000 OA and RA patients in which low dose aspirin use was allowed was 0.68% on celecoxib alone and 1.08% on celecoxib with or without aspirin.
Studies have shown that patients with a prior history of peptic ulcer disease and/or gastrointestinal bleeding and who use NSAIDs, have a greater than 10-fold higher risk for developing a GI bleed than patients with neither of these risk factors.

Anaphylactic reactions
As with NSAIDs in general, anaphylactic reactions have occurred in patients without known prior exposure to celecoxib. In post-marketing experience, rare cases of anaphylactic reactions and angioedema have been reported in patients receiving celecoxib. Celecoxib should not be given to patients with the aspirin triad. This symptom complex typically occurs in asthmatic patients who experience rhinitis with or without nasal polyps, or who exhibit severe, potentially fatal bronchospasm after taking aspirin or other NSAIDs (see CONTRAINDICATIONS and PRECAUTIONS, Pre-existing Asthma). Emergency help should be sought in cases where an anaphylactic reaction occurs.

Use in Patients with Inflammatory Bowel Disease (IBD)
Short-term exposure of celecoxib to patients with ulcerative colitis (UC) in remission has not shown an exacerbation of IBD in spondyloarthropathies, but the implications of longer term exposure remain unknown. NSAIDs have been associated with an exacerbation of IBD associated with spondyloarthropathies.

### 6.2. Summary of findings from non-clinical studies

**Ipilimumab** has specificity and a high affinity for human CTLA-4. The calculated dissociation constant value from an average of several studies was 5.25 nM. Binding of ipilimumab to purified, recombinant human CTLA-4 antigen was also demonstrated by enzyme-linked immunosorbent assay with half-maximal binding at 15 ng/mL, whereas saturation was observed at approximately 0.1g/mL. No cross-reactivity was observed against human CD28.

Ipilimumab completely blocked binding of B7.1 and B7.2 to human CTLA-4 at concentrations higher than 6 and 1 g/mL, respectively.

**Nivolumab** has been shown to bind specifically to the human PD-1 receptor and not to related members of the CD28 family. Nivolumab inhibits the interaction of PD-1 with its ligands, PD-L1 and PD-L2, resulting in enhanced T-cell proliferation and interferon-gamma (IFN-γ) release in vitro. Nivolumab binds with high affinity to activated human T-cells expressing cell surface PD-1 and to cynomolgus monkey PD-1. In a mixed lymphocyte reaction (MLR), nivolumab promoted a reproducible concentration-dependent enhancement of IFNy release.

**Celecoxib:** In-vivo and ex-vivo studies show that celecoxib has a very low affinity for the constitutively expressed COX-1 enzyme. Consequently, at therapeutic doses celecoxib has no effect on prostanoids synthesized by activation of COX-1 thereby not interfering with normal COX-1 related physiological processes in tissues, particularly the stomach, intestine and platelets.

### 6.3. Summary of findings from clinical studies

**Ipilimumab:** Bristol-Myers Squibb (BMS) and Medarex, Inc. (MDX, acquired by BMS in Sep-2009) have co-sponsored an extensive clinical development program for ipilimumab, encompassing more than 19,500 subjects (total number of subjects enrolled in ipilimumab studies) in several cancer types in completed and ongoing studies, as well as a compassionate use program. The focus of the clinical program is in melanoma, prostate cancer, and lung cancer, with advanced melanoma being the most comprehensively studied indication. Ipilimumab is being investigated both as monotherapy and in combination with other modalities such as chemotherapy, radiation therapy, and other immunotherapies. Phase 3 programs are ongoing in melanoma, prostate cancer, and lung cancer. In melanoma, 2 completed Phase 3 studies (MDX010-20 and CA184024) have demonstrated a clinically meaningful and statistically significant survival benefit in pretreated advanced melanoma and previously untreated advanced melanoma, respectively. See Ipilimumab (Yervoy) Summary of Product Characteristics [SmPC]) for further details.

**Nivolumab** has demonstrated durable responses exceeding 6 months as monotherapy and in combination with ipilimumab in several tumor types, including NSCLC, melanoma, RCC, and some lymphomas In confirmatory trials, nivolumab as monotherapy demonstrated a statistically significant improvement in OS as compared with the current standard of care in subjects with advanced or metastatic NSCLC and in subjects with unresectable or metastatic melanoma. Nivolumab in combination with ipilimumab improved PFS and ORR over ipilimumab alone in subjects with unresectable or metastatic melanoma.

***For more information see the Investigator's Brochure, section 5.3.1.***

### 6.4. Summary of known and potential risks and benefits

Participation in this trial sets the patients at risk of developing immune related adverse events. Algorithms have been developed to treat patients developing irAEs. Recovery is commonly observed (except for endocrine irAE) and depends on the fast onset of the advised immunosuppression.

The overall safety experience with nivolumab, as a monotherapy or in combination with other therapeutics, is based on experience in approximately 8,600 subjects treated to date.

For monotherapy, the safety profile is similar across tumor types. There is no pattern in the incidence, severity, or causality of AEs to nivolumab dose level. In Phase 3 controlled studies, the safety profile of nivolumab monotherapy is acceptable in the context of the observed clinical efficacy, and manageable using established safety guidelines. Clinically relevant AEs typical of stimulation of the immune system were infrequent and manageable by delaying or stopping nivolumab treatment and timely immunosuppressive therapy or other supportive care.

In several ongoing clinical trials, the safety of nivolumab in combination with other therapeutics such as ipilimumab, cytotoxic chemotherapy, anti-angiogenics, and targeted therapies is being explored. Most studies are ongoing and, as such, the safety profile of nivolumab combinations continues to evolve. The most advanced combination under development is nivolumab + ipilimumab in subjects with unresectable or metastatic melanoma. Results to date suggest that the safety profile of nivolumab + ipilimumab combination therapy is consistent with the mechanisms of action of nivolumab and ipilimumab. The nature of the AEs is similar to that observed with either agent used as monotherapy; however, both frequency and severity of most AEs are increased with the combination.

### 6.5. Description and justification of route of administration and dosage

Nivolumab and ipilimumab are both monoclonal antibodies and will be given together on day 1, followed by a second cycle of nivolumab on day 15.

Nivolumab dose = 3 mg/kg

Ipilimumab dose = 1 mg/kg

On day 1 when study drugs (ipilimumab or nivolumab) are to be administered on the same day, separate infusion bags and filters must be used for each infusion. Nivolumab must be administered first. The second infusion with ipilimumab will start approximately 30 minutes after completion of the nivolumab infusion.

BMS-936558 (nivolumab) is to be administered as a 60-minute IV infusion. Ipilimumab should be administered as a 90-minute infusion following.

Ipilimumab and nivolumab may be diluted in 0.9% Sodium Chloride Solution or 5% Dextrose solution.

The dosing calculations should be based on the body weight. If the subject's weight on the day of dosing differs by > 10% from the weight used to calculate the dose, the dose must be recalculated. All doses should be rounded up or to the nearest milligram per institutional standard.

Following the first dose of the combination of nivolumab and ipilimumab, nivolumab will be given after two weeks at a dose of 3 mg/kg. Patients may be dosed no less than 12 days from the previous dose of drug; and dosed up to 3 days after the scheduled date if necessary.

### 6.6. Dosages, dosage modifications and method of administration

Dose reductions or dose escalations are not permitted.

### 6.7. Preparation and labelling of Investigational Medicinal Product

Preparation and labeling of the investigational medicinal products will be performed by the pharmacy of the NKI according to in-house SOP. For additional information see the Pharmacy Reference Material and investigator brochure.

### 6.8. Drug accountability

Drug accountability will be performed by the pharmacy of the NKI according to internal Standards.

# 7. METHODS

## 7.1. Study parameters/endpoints

### 7.1.1. Main study parameter/endpoint

Safety will be measured by SAEs and SUSARS and treatment related complications leading to delay in surgery(that according to the study timelines should be performed in week 5-6).. Pre-operative treatment-related complications include all immune-related adverse events, attributable to the study medication, that lead to delay in surgery, with colitis being the most important, or delays due to the treatment of immunotherapy related toxicity.. Logistical reasons or non-study-medication related complications (i.e. bacterial infections) leading to delay in surgery will not be considered dose-limiting toxicity. Post-operative complications including, but not limited to anastomotic dehiscence, wound dehiscence, abscess, perforation, bleeding and infection will be recorded.

Feasibility will be measured by adherence to the timelines in the study protocol.

For the run-in period, post-operative follow-up will be recorded and post-operative complications will be studied in relation to the study medication. The last time-point will be the end of study treatment, this being 3 weeks after surgery.

To meet this endpoint, all patients will be discussed in our immunotherapy team meeting (consisting of at least a medical oncologist and gastro-intestinal surgeon) the week before surgery, to evaluate whether immunotherapy related toxicity or treatment of immunotherapy-related toxicity should lead to delay in surgery.

In case of immunotherapy related toxicity leading to delay in surgery or study-medication attributable post-operative complications, another 3 patients will be included (see paragraph 3).

### 7.1.2. Secondary study parameters/endpoints

- Primary readout will be the effect of therapy on intratumoral T-cell infiltration, CD4/CD8 ratio and immune checkpoints upregulation in the time interval pre- and post-treatment in biopsies. This will be compared in paired biopsies and between the different groups according to microsatellite status and treatment allocation.

  For T-cell infiltration, multiplex biomarker imaging and quantitative analyses will be performed using immunofluorescent staining. For the primary endpoint, a set panel will be used for CD3, CD4, CD8, CD45RO, FOXP3, CD68/CD163 (MDSCs), M2 macrophages and PD-L1. Depending on the amount of tissue available from pre-therapy biopsies, a post-hoc analysis of other biomarkers and checkpoints will be performed.

Mismatch repair proteins will be analyzed using IHC for microsatellite status determination.
At randomization, the microsatellite status of the tumor and confirmation of at least 30% tumor cells in the biopsies must be completed. Upon acquisition of the biopsies, this information should be available within approximately 2 days.

- Immunogenic mutational load will be determined by tumor tissue DNA WES. Peripheral blood DNA WES will also be performed and used as a control for somatic mutation sorting (only genes relating to colon cancer and/or immune-related genes, deemed informational for this study, will be assessed).
- Immune suppressive pathways, IFN-y induced gene expression and COX2 induced gene expression changes will be analyzed by use of RNA sequencing on pre- and post-therapy tissue;
- Date of relapse, as determined by disease recurrence or disease-related death during follow-up after surgery. Follow-up will be performed according to local and/or national guidelines;
- The Immunoscore will be determined using information obtained from analyses for the main secondary endpoint, using quantification of cytotoxic and memory T cells in the core of the tumor (CT) and in the tumor's invasive margin (IM) (**Appendix E)**;
- Organoids cultured from both normal and tumor tissue pre- and post-therapy using biopsies obtained by colonoscopy and at resection for a selected group of patients based on findings within the TME and availability of sufficient material;

### 7.2. Randomisation, blinding and treatment allocation

Single-center, open-label, exploratory study. Minimization for MSS tumors will be done by computer, using ALEA® software.

### 7.3. Study procedures

After informed consent is signed, patients will be registered.
***See table 2 for all study assessments***

**Before randomization** and start of treatment, the following study-related procedures/ assessments will be performed:

- **colonoscopy** will be planned within 5 work days of registration. Colonoscopy will be performed according to local guidelines. At least 12 tumor biopsies and 3 normal tissue biopsies will be collected during colonoscopy for FFPE, frozen tissue and organoid culture. If for any reason 12 biopsies is not possible, a minimum of 8 biopsies will be

required for treatment in the study, containing sufficient tumor content (>30% tumor cells);

- **pathological confirmation** of adenocarcinoma;
- **microsatellite status** by IHC;
- **medical history;**
- physical examination (including height, weight, blood pressure, pulse, lungs/heart/ abdomen) and ECOG performance status;
- **blood draw** before randomization in the study will include complete blood count w/ differential, liver function tests, serum urea levels, creatinine, Ca, Mg, Na, K, Cl, LDH, CEA, Glucose, amylase, lipase, TSH, Free T4, B-HCG pregnancy test for WOBCP, hepatitis B antigen, hepatitis C antibody, PT/INR, APTT. *
- **blood samples** for PBMCs and whole plasma biobanking and storage (see page 38);
- **radiological** tumor staging by CT-scan;
- **ECG;**
- **randomization** will be performed as soon as the microsatellite status of the tumor is known (within + 2 work days of colonoscopy or based on previous biopsies).

**Start of therapy** with the study drugs will be 7( +/- 2) days after registration. Treatment will be administered via intravenous infusion at the Antoni van Leeuwenhoek outpatient clinic according to procedures described in paragraph 6.5;

- **second cycle of therapy** consisting of nivolumab only, will be administered no less than 12 days from the previous dose and up to 3 days after the scheduled date.
- patients randomized for group 2 will receive daily celecoxib 200mg capsules from day 1 until the day before surgery.

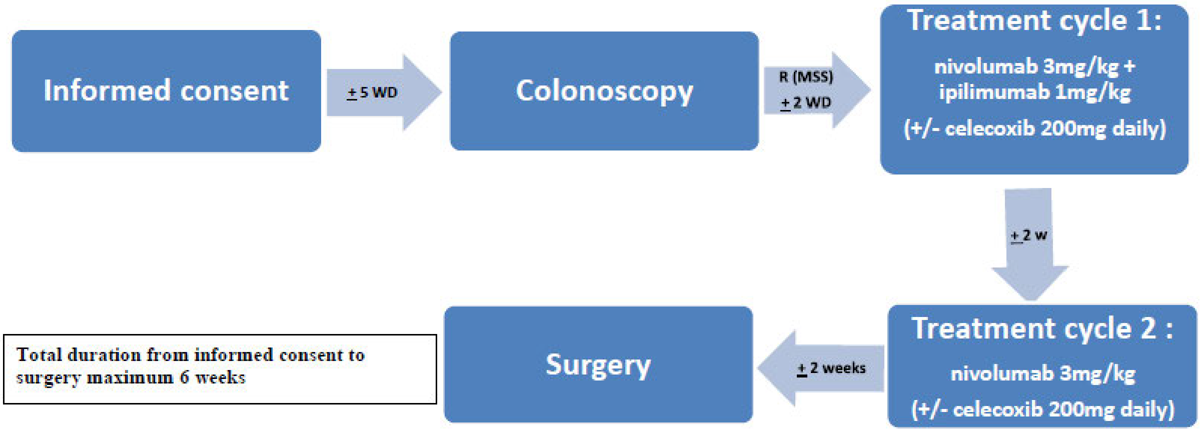


**Figure 2: schematic overview of NICHE study.**

**During treatment:**

- **laboratory testing** (complete blood count w/ differential, liver function tests, serum urea levels, creatinine, Ca, Mg, Na, K, Cl, LDH, Glucose, amylase, lipase, TSH, Free T4, B-HCG pregnancy test for WOBCP; *
- **blood samples** for PBMCs and whole plasma biobanking and storage (translational research purposes);
- **vital signs (**blood pressure, pulse), weight, ECOG performance status and symptom directed physical exam;
- **adverse events;**

**Surgery:**

- **surgery** will be planned at 5 (+/- 1) weeks after date of registration;
- **tissue collection:** all tissue (tumor, normal and lymphoid) that is not used for diagnostic purposes will be stored for study analyses and translational purposes;
- **peri-operative** collection of tumor material, normal tissue and lymph nodes will be done at surgical resection and processed by the pathology department. All leftover material, which is not needed for standard diagnostics, will be biobanked for future analyses.

**Post-operative / end of study treatment (3 weeks, maximum of 28 days);**

- **laboratory resting:** complete blood count w/ differential, liver function tests, serum urea levels, creatinine, Ca, Mg, Na, K, Cl, LDH, Glucose, amylase, lipase, TSH, Free T4, B-HCG pregnancy test for WOBCP, hepatitis B antigen, hepatitis C antibody;
- **blood samples** for biobanking (translational research purposes);
- **adverse events;**

**Follow-up**

- follow-up will continue according to local and national guidelines. for **SAEs**, follow-up will be 100 days after last study drug administration.
- **disease status** and signs of recurrence will be evaluated according to national and local guidelines.

*laboratory results required before dosing of study medication will be repeated if >72 hours old at the time of infusion.

All samples (i.e. organoids, blood, normal and tumor tissue, lymphoid tissue) will be anonymized using a unique patient identification code (see Chapter on Confidentiality of patients). Biopsies will be used for: 1. Immunohistochemistry for proposed molecules and assessment of microsatellite status, 2. Organoid culture and 3. Evaluation of other objectives

(including but not limited to DNA sequencing, RNA sequencing). Blood samples will be used for: 1. Germline DNA, 2. Biomarker research. All patient material will be stored at the primary research site (Antoni van Leeuwenhoek, Netherlands Cancer Institute).
If the study-related biopsy procedure was combined with a histological biopsy for diagnostic reasons (i.e. confirmation of adenocarcinoma), the biopsy samples will be divided in the following order of priority: MMR protein status, translational objectives/endpoints as mentioned in paragraph 2, organoid culture, other translational/exploratory endpoints. Biopsies not used for organoid culture will remain stored until diagnostic pathological assessment has been completed. When the diagnostic biopsy samples are not sufficient for their purpose, the study biopsy samples will be pulled out of the study and will be used for diagnostics (which we prioritize for the patient's wellbeing). In the unlikely event that even diagnostic assessment cannot be completed on the study biopsy samples, patients can be asked for renewed diagnostic biopsy combined with study related histological biopsy. The biopsy specimen intended to be used for organoid cultures is mandated to be kept in a special medium directly following the biopsy procedure. This prevents its use for diagnostic purposes should initial diagnostic pathological assessment fail.
**Tumor organoids** requirements**:** when available, one or more biopsy specimens will be mandated for tumor organoid culture. Biopsy specimens will be deposited in a special medium immediately following the biopsy procedure. Tumor organoids will be cultured according to standardized protocols at the Antoni van Leeuwenhoek.
**Immunoscore:** scoring will be performed by quantification of CD4/CD45RO T-cells in the core of the tumor and invasive margin, as described in **appendix E.**

### 7.4. Withdrawal of individual subjects

Subjects can leave the study at any time for any reason if they wish to do so without any consequences. The investigator can decide to withdraw a subject from the study for urgent medical reasons. Patients will receive standard treatment should they decide to withdraw from the study.

### 7.5. Replacement of individual subjects after withdrawal

Patients who withdraw consent will be replaced.
If for a specific patient pre-treatment material is insufficient for all mandatory analyses for the main secondary endpoint, this patient will not be included in the study and will be replaced. If for any reason a patient is not treated with the intended regimen or received at least 1 cycle of study medication, this patient will also be replaced.

### 7.6. Follow-up of subjects withdrawn from treatment

Patients who receive at least one cycle of the intended treatment will be followed-up according to the assessment schedule.

Subjects who request to discontinue study drug will remain in the study and must continue to be followed for protocol specified follow-up procedures. The only exception to this is when a subject specifically withdraws consent for any further contact with him/her or persons previously authorized by subject to provide this information.

| **Table 2 Schedule of assessments** | **Registration/baseline** | | **Week 0 Cycle 1** [1] | **Week 2 Cycle 2** [2] | **Surgery** [3] | **Week + 3 after surgery**[4] **End of study treatment** | **Follow-up**[5] |
|---|---|---|---|---|---|---|---|
| | **Week -4 to 0** | **≤7 days** | **Day 1** | | | | |
| Informed consent [a] | x | | | | | | |
| Medical History | x | | | | | | |
| Physical examination | x[b] | | x[c] | x[c] | | | |
| ECOG performance status [d] | x | | x | x | | x | |
| Colonoscopy [e] | | x | | | | | |
| Ipilimumab administration [f] | | | x | | | | |
| Nivolumab administration [g] | | | x | x | | | |
| Celecoxib administration daily [h] | | | x | x | | | |
| Pathological confirmation of adenocarcinoma | | x | | | | | |
| Microsatellite status by IHC [i] | | x | | | | | |
| Hematology [j] | | x | x | x | x | x | |
| Chemistry [k] | | x | x | x | x | x | |
| PBMCs, plasma [l] | | x | x | x | x | x | |
| Coagulation: PT/INR and aPTT | | x | | | x | | |
| Beta-HCG pregnancy test [m] | | x | x | x | | | |
| Serology [n] | | x | | | | | |
| CT-scan | x [o] | | | | | | |

| | | | | | | | |
|---|---|---|---|---|---|---|---|
| ECG | | x | | | | | |
| Adverse events | | x | x | x | x | x | x [p] |
| Tissue collection [q] | | | | | x | | |
| Disease status | | | | | | | x |
| Co-medication | x | x | x | x | x | x | |

a) written informed consent must be obtained before performing any study-specific screening test or evaluations. However, results from routine assessments conducted prior to informed consent signature may be used as screening assessments
b) height, weight, vital signs (blood pressure, pulse), examination of lungs/heart/abdomen
c) weight, vital signs (blood pressure, pulse) and symptom directed physical exam
d) appendix A
e) 12 tumor tissue biopsies and 3 normal tissue biopsies
f) ipilimumab 1m/kg
g) nivolumab 3mg/kg
h) only if randomized for group 2
i) within ± 2 work days of colonoscopy or based on previous biopsies
j) within 72 hrs prior to (re-)dosing to include CBC w/ differential
k) within 72 hrs prior to (re-)dosing to include LFTs, BUN or serum urea level, creatinine, Ca, Mg, Na, K, Cl, LDH, CEA, Glucose, amylase, lipase, TSH (with Free T4)
l) 120ml (100ml for PBMCs and ctDNA)
m) women of childbearing potential
n) HbsAG, HCV antibody
o) CT-chest/abdomen
p) during 100 days after last study drug administration SAE reports only.
q) all leftover patient tissue, including tumor, normal and lymphoid tissue will be biobanked

1) within 7 days (+/- 2 days) of registration
2) no less than 12 days from the previous dose and up to 3 days after the scheduled date
3) no earlier than 4 weeks and no later than 6 weeks after registration
4) 3 weeks +/-1 week after surgery
5) follow-up according to national and local guidelines for disease recurrence

# 8. SAFETY REPORTING

## 8.1. Section 10 WMO event

In accordance to section 10, subsection 1, of the WMO, the investigator will inform the subjects and the reviewing accredited METC if anything occurs, on the basis of which it appears that the disadvantages of participation may be significantly greater than was foreseen in the research proposal. The study will be suspended pending further review by the accredited METC, except insofar as suspension would jeopardize the subjects' health. The investigator will take care that all subjects are kept informed.

## 8.2. AEs, SAEs and SUSARs

### 8.2.1. Adverse events (AEs)

An Adverse Event (AE) is defined as any new untoward medical occurrence or worsening of a preexisting medical condition in a clinical investigation subject administered an investigational (medicinal) product and that does not necessarily have a causal relationship with this treatment. An AE can therefore be any unfavorable and unintended sign (such as an abnormal laboratory finding), symptom, or disease temporally associated with the use of investigational product, whether or not considered related to the investigational product.
The causal relationship to study drug is determined by a physician and should be used to assess all adverse events (AE). The casual relationship can be one of the following:
Related: There is a reasonable causal relationship between study drug administration and the AE.
Not related: There is not a reasonable causal relationship between study drug administration and the AE.
The term "reasonable causal relationship" means there is evidence to suggest a causal relationship.
Adverse events can be spontaneously reported or elicited during open-ended questioning, examination, or evaluation of a subject. (In order to prevent reporting bias, subjects should not be questioned regarding the specific occurrence of one or more AEs.)

#### NONSERIOUS ADVERSE EVENT

A ***nonserious adverse event*** is an AE not classified as serious

- Nonserious Adverse Events are to be provided to BMS in aggregate via interim or final study reports as specified in the agreement or, if a regulatory requirement [e.g. IND US trial] as part of an annual reporting requirement.

- Nonserious AE information should also be collected from the start of a placebo lead-in period or other observational period intended to establish a baseline status for the subjects.

**Nonserious Adverse Event Collection and Reporting**

The collection of nonserious AE information should begin at initiation of study drug. All nonserious adverse events (not only those deemed to be treatment-related) should be collected continuously during the treatment period and for a minimum of 21 days following surgery.

Nonserious AEs should be followed to resolution or stabilization, or reported as SAEs if they become serious. Follow-up is also required for nonserious AEs that cause interruption or discontinuation of study drug and for those present at the end of study treatment as appropriate.

### 8.2.2. Serious adverse events (SAEs)

A serious adverse event is any untoward medical occurrence that at any dose:

- results in death
- is life-threatening (defined as an event in which the subject was at risk of death at the time of the event; it does not refer to an event which hypothetically might have caused death if it were more severe)
- requires inpatient hospitalization or causes prolongation of existing hospitalization (see **NOTE** below)
- results in persistent or significant disability/incapacity
- is a congenital anomaly/birth defect
- is an important medical event (defined as a medical event(s) that may not be immediately life-threatening or result in death or hospitalization but, based upon appropriate medical and scientific judgment, may jeopardize the subject or may require intervention [eg, medical, surgical] to prevent one of the other serious outcomes listed in the definition above.) Examples of such events include, but are not limited to, intensive treatment in an emergency room or at home for allergic bronchospasm; blood dyscrasias or convulsions that do not result in hospitalization.)
- Potential drug induced liver injury (DILI) is also considered an important medical event.
- Suspected transmission of an infectious agent (eg, pathogenic or nonpathogenic) via the study drug is an SAE.

**NOTE**:

The following hospitalizations are not considered SAEs in BMS clinical studies:

- a visit to the emergency room or other hospital department < 24 hours, that does not result in admission (unless considered an important medical or life-threatening event)
- elective surgery, planned prior to signing consent
- admissions as per protocol for a planned medical/surgical procedure
- routine health assessment requiring admission for baseline/trending of health status (eg, routine colonoscopy)
- Medical/surgical admission other than to remedy ill health and planned prior to entry into the study. Appropriate documentation is required in these cases
- Admission encountered for another life circumstance that carries no bearing on health status and requires no medical/surgical intervention (eg, lack of housing, economic inadequacy, caregiver respite, family circumstances, administrative reason).

For specific procedures regarding the handling of serious adverse events, see appendix.

**Reporting of SAEs**

Following the subject's written consent to participate in the study, all SAEs, whether related or not related to study drug, must be collected, including those thought to be associated with protocol-specified procedures. All SAEs must be collected that occur within 100 days of discontinuation of dosing and documented and reported (the study specific SAE form should be used) within 24 hours to the Trial Office by e-mail (trial@nki.nl) or phone. The Trial Office will forward the SAE's to the NKI-AVL safety desk. .

In addition, serious adverse events will be reported by the NKI-AVL Safety Desk to the METC and the CCMO. All SAE's will be reported once yearly, as described in the section.

All SAEs must be collected that occur during the screening period. If applicable, SAEs must be collected that relate to any protocol-specified procedure (eg, a follow-up skin biopsy). The investigator should report any SAE that occurs after these time periods that is believed to be related to study drug or protocol-specified procedure.

SAEs, whether related or not related to study drug, and pregnancies must be reported to BMS within 24 hours. SAEs must be recorded on BMS or an approved form; pregnancies on a Pregnancy Surveillance Form.

**SAE Email Address:** Worldwide.Safety@BMS.com

**SAE Facsimile Number:** 609-818-3804

If only limited information is initially available, follow-up reports are required. (Note: Follow-up SAE reports should include the same investigator term(s) initially reported.)

If an ongoing SAE changes in its intensity or relationship to study drug or if new information becomes available, a follow-up SAE report should be sent within 24 hours to the BMS (or designee) using the same procedure used for transmitting the initial SAE report.

All SAEs should be followed to resolution or stabilization.

The following laboratory abnormalities should be documented and reported appropriately:

- any laboratory test result that is clinically significant or meets the definition of an SAE
- any laboratory abnormality that required the subject to have study drug discontinued or interrupted
- any laboratory abnormality that required the subject to receive specific corrective therapy.

**Pregnancy**

If, following initiation of the investigational product, it is subsequently discovered that a study subject is pregnant or may have been pregnant at the time of investigational product exposure, including during at least 6 half-lives after product administration, the investigational product will be permanently discontinued in an appropriate manner (eg, dose tapering if necessary for subject safety).

The investigator must immediately notify Worldwide Safety @BMS of this event via the Pregnancy Surveillance Form in accordance with SAE reporting procedures.

Follow-up information regarding the course of the pregnancy, including perinatal and neonatal outcome and, where applicable, offspring information must be reported on the Pregnancy Surveillance Form [provided upon request from BMS]

Any pregnancy that occurs in a female partner of a male study participant should be reported to BMS. Information on this pregnancy will be collected on the Pregnancy Surveillance Form.

**Overdose**

An overdose is defined as the accidental or intentional administration of any dose of a product that is considered both excessive and medically important. All occurrences of overdose must be reported as an SAE.

**Other Safety Considerations**

Any significant worsening noted during interim or final physical examinations, electrocardiograms, x-rays, and any other potential safety assessments, whether or not

these procedures are required by the protocol, should also be recorded as a nonserious or serious AE, as appropriate, and reported accordingly.

The Sponsor/Investigator will ensure that all SAEs in the clinical database are reported to BMS and any applicable health authority during the conduct of the study. This reconciliation will occur at least quarterly and be initiated by the sponsor/investigator. Sponsor/investigator will request a reconciliation report from: aepbusinessprocess@bms.com. During reconciliation, any events found to not be reported previously to BMS must be sent to Worldwide.Safety@BMS.com.

#### 8.2.3. Suspected unexpected serious adverse reactions (SUSARs)

Adverse reactions are all untoward and unintended responses to an investigational product related to any dose administered.

Unexpected adverse reactions are SUSARs if the following three conditions are met:

1. the event must be serious (see chapter 9.2.2);
2. there must be a certain degree of probability that the event is a harmful and an undesirable reaction to the medicinal product under investigation, regardless of the administered dose;
3. the adverse reaction must be unexpected, that is to say, the nature and severity of the adverse reaction are not in agreement with the product information as recorded in:
   - Summary of Product Characteristics (SPC) for an authorised medicinal product;
   - Investigator’s Brochure for an unauthorised medicinal product.

The sponsor (AVL safety desk) will report expedited the following SUSARs through the web portal *ToetsingOnline* to the METC:

SUSARs that have arisen in the clinical trial that was assessed by the METC;

SUSARs that have arisen in other clinical trials of the same sponsor and with the same medicinal product, and that could have consequences for the safety of the subjects involved in the clinical trial that was assessed by the METC.

The remaining SUSARs are recorded in an overview list (line-listing) that will be submitted once every half year to the METC. This line-listing provides an overview of all SUSARs from the study medicine, accompanied by a brief report highlighting the main points of concern. The expedited reporting of SUSARs through the web portal ToetsingOnline is sufficient as notification to the competent authority.

The sponsor will report expedited all SUSARs to the competent authorities in other Member States, according to the requirements of the Member States.

The expedited reporting will occur not later than 15 days after the sponsor has first knowledge of the adverse reactions. For fatal or life threatening cases the term will be maximal 7 days for a preliminary report with another 8 days for completion of the report.

### 8.3. Annual safety report

In addition to the expedited reporting of SUSARs, the sponsor will submit, once a year throughout the clinical trial, a safety report to the accredited METC, competent authority, and competent authorities of the concerned Member States.

This safety report consists of:

a list of all suspected (unexpected or expected) serious adverse reactions, along with an aggregated summary table of all reported serious adverse reactions, ordered by organ system, per study;

a report concerning the safety of the subjects, consisting of a complete safety analysis and an evaluation of the balance between the efficacy and the harmfulness of the medicine under investigation.

### 8.4. Follow-up of (serious) adverse events

All AEs will be followed until they have abated, or until a stable situation has been reached. Depending on the event, follow up may require additional tests or medical procedures as indicated, and/or referral to the general physician or a medical specialist.

SAEs need to be reported till 100 days after the last study drug administration within the Netherlands, as defined in the protocol. The end of study treatment is defined as the patient’s last visit at 3 weeks after surgery, with a maximum of 28 days.

### 8.5. Data Safety Monitoring Board (DSMB) / Safety Committee

No DSMB will be set-up.

## 9. STATISTICAL ANALYSIS

A total of 60 patients will be enrolled within two years, including 30 patients with MSS tumors and 30 patients with MSI tumors. If during the study the Immunoscore cannot be determined based on primary tumor biopsies, inclusion of patients with MSI tumors will be limited to 20 total. Findings within the TME of MSI tumors will be the main source of information on the read-outs to be used for the TME of MSS tumors. The immunoscore, using the scoring system by Galon et al (appendix E), will be performed on all tumors using biopsy material. Based on recent findings, we expect 30-40% of MSS and 60-70% of MSI primary colon tumors to have high immunoscores.
As the first part of this study is completed and analysis of findings in this cohort lead to the addition of new compounds, there will be a new sample size justification.

The following analysis populations will be used for analysis of the trial:

- **Intention-to-treat population (ITT):** all patients will be analyzed in the arm they were allocated;
- **Per protocol population (PP):** all patients who received at least one dose of the study drugs;

A patient will be considered to be eligible if he/she did not have any deviations from the patient entry criteria listed of the protocol. Potential eligibility problems will be assessed by the investigator at time of medical review.

Descriptive statistics for continuous variables will include total counts, mean, median, standard deviation (SD), range and interquartile range (IQR). Comparison of continuous variables between groups or strata will be performed using parametric (t-test/ANOVA) or non-parametric (Mann Whitney U-test/Kruskall-Wallis test) tests depending on the distribution. For paired data, the dependent samples t-test or Wilcoxon signed-rank test will be used.

Categorical variables will be presented as frequencies and percentages. Comparison of the categorical variables will be done using Fisher's exact test.

Binary endpoints will be presented as a proportion with exact 2-sided 95% confidence intervals.

Time-to-event endpoints will be analyzed using the Kaplan Meier method. Differences between groups or strata will be tested using the log-rank test.

### 9.1. Primary study parameters

Safety analyses will be performed on the **PP**. Safety data will be displayed by treatment arm and summarized with descriptive statistics. The worst toxicity grade over all cycles according to the CTCAE criteria version 4.03 will be displayed by treatment arm and by body system.

### 9.2. Secondary study parameters

- Summary statistics (median, interquartile range and standard deviation) will be presented for differences between groups and changes within patients in tumor-infiltrating lymphocytes, CD8+ T-cells and CD4/CD8 ratio will be quantified and presented in a mainly descriptive manner using the median and range (minimum, maximum) or mean (variance) depending whether the distribution appears symmetrical. When possible, these differences will be tested using the student's t-test for independent and paired samples or non-parametric tests. Comparisons between the tumor and treatment groups will be conducted: in paired (same-patient) biopsies, MSS vs MSI and MSS group 1 vs MSS group 2 and plotted in either scatterplots or time-series graphs. If the immunoscore is deemed feasible during the study, differences in abovementioned markers between immunoscore high and low tumors will be tabulated and plotted in bar graphs.
- Frequency tables will be tabulated (by treatment group or otherwise) for all categorical variables by the levels of the variables of the ITT. Continuous variables (for example age.) are presented in a mainly descriptive manner using the median and range (minimum, maximum) or mean (variance) depending whether the distribution appears symmetrical;
- Summary tables (descriptive statistics and/or frequency tables) will be provided for all baseline demographic characteristics and clinical data of the **ITT** (following CONSORT-guidelines 2010, where applicable). Continuous variables will be summarized with descriptive statistics (n, mean, standard deviation, range and median).
- For exploratory analyses, means will be compared using either student t-test if validity conditions are fulfilled or using non parametric Wilcoxon-Mann-Whitney tests. Proportions will be compared using either Chi2 statistics or Fisher's exact test depending on validity conditions;

- Description of number of cycles of therapy for both study drugs (**PP**);
- Relapse-free survival (time from surgery to relapse or disease-related death) will be described using the Kaplan-Meier method and differences in survival curves for MSS vs MSI and MSS group 1 vs MSS group 2 will be tested using the log rank test. The **PP** set will be used for this analysis. Patients without a relapse at the end of follow-up or with death unrelated to disease will be censored at that time;

# 10. ETHICAL CONSIDERATIONS

## 10.1. Regulation statement

The study will be conducted according to the principles of the Declaration of Helsinki, adopted by the by the 18th WMA General Assembly, Helsinki, Finland, June 1964, and amended by the 64th WMA General Assembly, Fortaleza, Brazil, October 2013 and in accordance with the Medical Research Involving Human Subjects Act (WMO) and other guidelines, regulations and Acts.

The protocol has been written, and the study will be conducted according to the ICH Harmonized Tripartite Guideline for Good Clinical Practice (ref: http://www.ich.org/fileadmin/Public_Web_Site/ICH_Products/Guidelines/Efficacy/E6/E6_R1_Guideline.pdf). The protocol will be approved by the local ethics committees.

## 10.2. Recruitment and consent

All patients will be informed on the aims of the study, the possible adverse events, the procedures and possible hazards to which he/she will be exposed, and the mechanism of treatment allocation. They will be informed as to the strict confidentiality of their patient data, but that their medical records may be reviewed for trial purposes by authorized individuals other than their treating physician.

It will be emphasized that participation is voluntary and that the patient is allowed to refuse further participation in the protocol whenever he/she wants. This will not prejudice the patient's subsequent care. Documented informed consent (IC) will be obtained for all patients included in the study before they are registered in the study. This will be done in accordance with the national and local regulatory requirements. Patients will be given sufficient time for consideration. An independent physician will be available in accordance with the requirements of the national law.

The informed consent procedure will be according to the ICH guidelines on Good Clinical Practice. This implies that "the written IC form will be signed and personally dated by patient or the patient's legally accepted representative".

## 10.3. Benefits and risks assessment, group relatedness

Currently, no pre-operative treatment is given to patients with colon cancer. Post-surgery adjuvant chemotherapy is commonly applied to patients with stage 3 tumors and in some cases in stage 2 tumors, which has been shown to marginally increase PFS and OS, the latter by approximately 5%.

Patients included in this study will be exposed to two immunotherapeutic agents (nivolumab and ipilimumab) and one group of patients will also receive a COX2 inhibitor. Nivolumab and

ipilimumab are known to induce immune related adverse events, especially when combined. However, the exposure to immunotherapeutic drugs will be limited to two courses total. The chosen dosing and schedule has been/is being used in trials, where preliminary data suggest similar efficacy and less serious adverse events than other schedules.[12] Furthermore, surgical complications do not seem to increase after treatment with immune checkpoint inhibitors. According to a systematic review by Baker et al (Am Surgeon 2014), no surgical complications were documented in patients receiving ipilimumab and no postoperative complications could be attributed to ipilimumab. Preliminary reports thus suggest there is **no reason to withhold or delay surgery** for patients receiving ipilimumab. Preliminary, unpublished, results of patients receiving neoadjuvant anti-PD1 also suggest no increase in surgical complications.

Previous reports of clinical studies using combinations of ipilimumab and nivolumab show grade 3-4 adverse events in 44-58% of patients, with 37% of treatments-related events leading to discontinuation of treatment. Concerning safety and toxicity of the proposed combination of ipilimumab 1mg/kg once only and nivolumab 3mg/kg administered twice, data from the Checkmate-012 trial were extrapolated. In this study, several schemes of combination treatment were applied, with the closest one to our proposed scheme being ipilimumab 1mg/kg every 12 weeks and nivolumab 3 mg/kg every two weeks. In this cohort of the study, treatment related adverse events were observed in 74% of subjects, with the most common being skin toxicity (39%), gastro-intestinal (18%), endocrine (8%), renal (8%) and hypersensitivity/infusion reactions (5%).

Grade 3 or grade 4 treatment related events were noted in 29% of subjects, with the most common events being gastro-intestinal (5%), renal (5%), endocrine, pulmonary (pneumonitis) and skin (3% each). There were no treatment-related deaths. Patients in this specific cohort received a median of 13 nivolumab doses and 3 ipilimumab doses.[31]

Additional safety data from the Checkmate-012 trial for the combination was reported in patients with advanced MSI mCRC, demonstrating grade 3-4 treatment-related events in 27% of subjects. The most common any grade event was diarrhea of grade 2 or less, occurring in 43% of patients. In this cohort, no grade 3-4 diarrhea was reported. [5]

These findings show a favorable toxicity profile for the alternatively dosed combination of nivolumab and ipilimumab, with a low frequency of grade 3 and 4 treatment related events. Also, considering the fact that patients in this study will only receive one single low dose of ipilimumab and two cycles of nivolumab, the incidence of treatment related events might be lower than abovementioned.

The added benefit of this treatment for patients is not yet known, since checkpoint blockade in colorectal cancer has not yet been thoroughly investigated in the non-metastatic setting. For patients with metastatic MSS tumors, there is limited benefit of immune checkpoint blockade. However, the biology of metastatic disease and primary, non-metastatic tumors may differ substantially, judging by amongst others the significantly decreased fraction of immunoscore Hi tumors in the metastatic setting in both MSS and MSI tumors.[6]
Three extra site visits will be required with participation in this study, where physical examination, toxicity assessment and blood draws will be performed. Patients will be asked to draw blood at inclusion, before every treatment cycle (twice total), at the time of surgery and 3 weeks post-operatively.

For immunotherapy to induce cancer cell death, sufficient activation of the immune system is needed. T cell activation requires two signals. The first signal is delivered via TCR upon antigen presentation by MHC on the APC. The second signal can be produced by a number so-called immune checkpoints and can be either co-stimulatory or co-inhibitory. PD-1 and CTLA-4 immune checkpoint molecules are often upregulated in tumor infiltrating T cells and by binding their corresponding ligands, often upregulated on cancer cells, downregulate the T cell response. Nivolumab and ipilimumab enhance T-cell antitumor activity by non-redundant, complementary mechanisms, leading to activation of antitumor immunity.[6,7]
Clinical experiences with nivolumab plus ipilimumab demonstrate deep and durable responses in previously treated melanoma and lung cancer.

*For more information, see paragraph 1.*

Previous exposure of human beings with the test product(s) and/or products with a similar biological mechanism

**Nivolumab** has demonstrated durable responses exceeding 6 months as monotherapy and in combination with ipilimumab in several tumor types, including NSCLC, melanoma, RCC, and some lymphomas In confirmatory trials, nivolumab as monotherapy demonstrated a statistically significant improvement in OS as compared with the current standard of care in subjects with advanced or metastatic NSCLC and in subjects with unresectable or metastatic melanoma. Nivolumab in combination with ipilimumab improved PFS and ORR over ipilimumab alone in subjects with unresectable or metastatic melanoma.

**For more information,** see paragraph 1 and 6.

### 10.4. Compensation for injury

The sponsor/investigator has a liability insurance which is in accordance with article 7 of the WMO.

The sponsor (also) has an insurance which is in accordance with the legal requirements in the Netherlands (Article 7 WMO). This insurance provides cover for damage to research subjects through injury or death caused by the study.
The insurance applies to the damage that becomes apparent during the study or within 4 years after the end of the study.

### 10.5. Incentives

Patients will not receive any incentive for study participation, including no compensation for travelling.

# 11. ADMINISTRATIVE ASPECTS, MONITORING AND PUBLICATION

## 11.1. Handling and storage of data and documents

Each patient will be assigned a Patient Allocation Number on registration. The Patient Allocation Number and the Patient Verification Code (Verification Code consists of the months (2 digits mm) and year (4 digits yyyy) of the date of birth of the patient) are to be entered on the electronic Case Report Form. The investigator will retain all pertinent information for a period of at least 15 years from study completion. The investigator will be responsible for retaining sufficient information about each patient (e.g. name, address, phone number, and identity in the study) so that regulatory agencies or participating investigators may access this information should the need to do so arise. These records should be retained in a confidential manner for as long as legally mandated according to local requirements.

The handling of personal data will comply with the Dutch Personal Data Protection Act (in Dutch: Wet Bescherming Persoonsgegevens, Wbp).

The list of staff members authorized to complete case report forms will be documented on the Delegation Log of the study. Delegated tasks will be documented in the delegation log.

## 11.2. Monitoring and Quality Assurance

Source data verification of the eCRFs and check of the Investigator Study File documents will be performed by the clinical research monitor of the NKI-AVL, according to the procedures described in the Monitor Plan.

## 11.3. Registration of the patients

Patient registration will only be accepted from authorized investigators or through their authorized data manager or authorized staff member. A patient can be randomized only after verification of eligibility. During the registration procedure the patients' informed consent are checked. Randomization will be done by the Trial Office of the AVL after receiving the original signed IC and after verification of the eligibility criteria.

## 11.4. Storage of patient material

Patient material, including normal colon tissue, lymph nodes and peripheral blood (derivatives), will be stored at the NKI-AVL. The tissue will be stored at the core facility molecular pathology and biobanking (CFMPB). The peripheral blood monocytes will be generated from peripheral blood by ficoll and stored in liquid nitrogen at the immunology

division of the NKI-AVL. Material that is not used for current translational research will be stored for up to 15 years.

### 11.5. Data management

All data that are relevant for the study will be collected on eCRFs developed by the data center of the AVL. All data that are relevant for the study will be collected in an eCRF. All SAE's, whether or not deemed drug related or expected, must be recorded in an eCRF AE- form. It is critical that the information provided on the SAE-form matches the information recorded in the eCRF for the same event.
The completed eCRFs must be reviewed, signed and dated by the Principal Investigator or sub-investigator.
Data cleaning procedures will be performed as described in de Data Validation Plan.
An overall description of all data management procedures will be documented in a study-specific Data Management Plan.

### 11.6. Amendments

All amendments will be notified to the METC that gave a favorable opinion.
A 'substantial amendment' is defined as an amendment to the terms of the METC application, or to the protocol or any other supporting documentation, that is likely to affect to a significant degree:

- the safety or physical or mental integrity of the subjects of the trial;
- the scientific value of the trial;
- the conduct or management of the trial; or
- the quality or safety of any intervention used in the trial.

All substantial amendments will be notified to the METC and to the competent authority.
Non-substantial amendments will not be notified to the accredited METC and the competent authority, but will be recorded and filed by the sponsor.

### 11.7. Annual progress report

The sponsor/investigator will submit a summary of the progress of the trial to the accredited METC once a year. Information will be provided on the date of inclusion of the first subject, numbers of subjects included and numbers of subjects that have completed the trial, serious adverse events/ serious adverse reactions, other problems, and amendments.

### 11.8. End of study report

The sponsor will notify the accredited METC and the competent authority of the end of the study within a period of 90 days. The end of the study treatment is defined as the patient's last visit. The end of follow-up will be the end of standard follow-up according to local and national guidelines. In case the study is ended prematurely, the sponsor will notify the accredited METC and the competent authority within 15 days, including the reasons for the premature termination. Within one year after the end of the study, the investigator/sponsor will submit a final study report with the results of the study, including any publications/abstracts of the study, to the accredited METC and the Competent Authority.

### 11.9. Public disclosure and publication policy

We foresee that the data acquired from this protocol will result in multiple publications. We will aim for publication in a high impact journal. The order in which authors will be mentioned in publications will be determined by the principal investigator.

# Appendix A: Performance Status Criteria

| WHO Performance Status Scale | | Karnofsky Performance Scale | |
|---|---|---|---|
| **Grade** | Descriptions | **Percent** | Description |
| 0 | Normal activity. Fully active, able to carry on all pre-disease performance without restriction. | 100 | Normal, no complaints, no evidence of disease. |
| | | 90 | Able to carry on normal activity; minor signs or symptoms of disease. |
| 1 | Symptoms, but ambulatory. Restricted in physically strenuous activity, but ambulatory and able to carry out work of a light or sedentary nature (e.g., light housework, office work) | 80 | Normal activity with effort; some signs or symptoms of disease. |
| | | 70 | Cares for self, unable to carry on normal activity or to do active work. |
| 2 | In bed <50% of the time. Ambulatory and capable of all self-care, but unable to carry out any work activities. Up and about more than 50% of waking hours. | 60 | Requires occasional assistance, but is able to care for most of his/her needs. |
| | | 50 | Requires considerable assistance and frequent medical care. |
| 3 | In bed >50% of the time. Capable of only limited self-care, confined in bed or chair more than 50% of waking hours. | 40 | Disabled, requires special care and assistance. |
| | | 30 | Severely disabled, hospitalization indicated. Death not imminent. |
| 4 | 100% bedridden. Completely disabled. Cannot carry on any self-care. Totally confined to bed or chair | 20 | Very sick, hospitalization indicated. Death not imminent. |
| | | 10 | Moribund, fatal processes progressing rapidly. |
| 5 | Deceased. | 0 | Deceased. |

# Appendix B: study medication preparation, handling and administration guidance

***Initial Orders***

- *Following submission and approval of the required regulatory documents, a supply of nivolumab and ipilimumab may be ordered from by completing a Drug Request Form provided by BMS for this specific trial.*
- *The initial order should be limited to the amount needed for two doses. Allow 5 business days for shipment of drug from BMS receipt of the Drug Request Form. Drug is protocol specific, but not patient specific. All drug products will be shipped by courier in a temperature-controlled container. It is possible that sites may have more than one nivolumab clinical study ongoing at the same time. It is imperative that only drug product designated for this protocol number be used for this study.*
- *Pharmacy supplies not provided by BMS: Empty IV bags/containers, approved diluents, In-line filters and infusion tubing*

***Re-Supply***

- *Drug re-supply request form should be submitted electronically at least 7 business days before the expected delivery date. Deliveries will be made Tuesday through Friday.*
- *When assessing need for resupply, institutions should keep in mind the number of vials used per treatment dose, and that shipments may take 14 business days from receipt of request. Drug is not patient-specific. Be sure to check with your pharmacy regarding existing investigational stock to assure optimal use of drug on hand.*

***Drug Excursions***

- *Drug excursions should be reported immediately to BMS on the form provided with the study-specific drug order form*

**For additional information regarding drug preparation and administration, see the Pharmacy Manual.**

# Appendix C: management algorithms

These general guidelines constitute guidance to the Investigator and may be supplemented by discussions with the Medical Monitor representing the Sponsor. The guidance applies to all immuno-oncology (I-O) agents and regimens. A general principle is that differential diagnoses should be diligently evaluated according to standard medical practice. Non-inflammatory etiologies should be considered and appropriately treated. Corticosteroids are a primary therapy for immuno-oncology drug-related adverse events. The oral equivalent of the recommended IV doses may be considered for ambulatory patients with low-grade toxicity. The lower bioavailability of oral corticosteroids should be taken into account when switching to the equivalent dose of oral corticosteroids.

## GI Adverse Event Management Algorithm

Rule out non-inflammatory causes. If non-inflammatory cause is identified, treat accordingly and continue I-O therapy. Opiates/narcotics may mask symptoms of perforation. Infliximab should not be used in cases of perforation or sepsis.

| Grade of Diarrhea/ Colitis (NCI CTCAE v4) | Management | Follow-up |
|---|---|---|
| **Grade 1**<br>Diarrhea:< 4 stools/day over baseline;<br>Colitis: asymptomatic | • Continue I-O therapy per protocol<br>• Symptomatic treatment | • Close monitoring for worsening symptoms.<br>• Educate patient to report worsening immediately<br>If worsens:<br>• Treat as Grade 2 or 3/4 |
| **Grade 2**<br>Diarrhea: 4-6 stools per day over baseline; IV fluids indicated <24 hrs; not interfering with ADL<br>Colitis: abdominal pain; blood in stool | • Delay I-O therapy per protocol<br>• Symptomatic treatment | If improves to grade 1:<br>• Resume I-O therapy per protocol<br>If persists > 5-7 days or recur:<br>• 0.5-1.0 mg/kg/day methylprednisolone or oral equivalent<br>• When symptoms improve to grade 1, taper steroids over at least 1 month, consider prophylactic antibiotics for opportunistic infections, and resume I-O therapy per protocol.<br>If worsens or persists > 3-5 days with oral steroids:<br>• Treat as grade 3/4 |
| **Grade 3-4**<br>Diarrhea (G3): ≥7 stools per day over baseline; incontinence; IV fluids ≥24 hrs; interfering with ADL<br>Colitis (G3): severe abdominal pain, medical intervention indicated, peritoneal signs<br>G4: life-threatening, perforation | • Discontinue I-O therapy per protocol<br>• 1.0 to 2.0 mg/kg/day methylprednisolone IV or IV equivalent<br>• Add prophylactic antibiotics for opportunistic infections<br>• Consider lower endoscopy | If improves:<br>• Continue steroids until grade 1, then taper over at least 1 month<br>If persists > 3-5 days, or recurs after improvement:<br>• Add infliximab 5 mg/kg (if no contraindication). Note: Infliximab should not be used in cases of perforation or sepsis |

Patients on IV steroids may be switched to an equivalent dose of oral corticosteroids (e.g. prednisone) at start of tapering or earlier, once sustained clinical improvement is observed. Lower bioavailability of oral corticosteroids should be taken into account when switching to the equivalent dose of oral corticosteroids.

**Suspicion of immune related colitis requiring corticosteroid therapy must be discussed with the study coordinator/PI for assessment of the need for steroid therapy. Infliximab may be prioritized over corticosteroids if surgery is imminent to reduce chances of post-operative complications.**

## Renal Adverse Event Management Algorithm

Rule out non-inflammatory causes. If non-inflammatory cause, treat accordingly and continue I-O therapy

| Grade of Creatinine Elevation (NCI CTCAE v4) | Management | Follow-up |
|---|---|---|
| **Grade 1**<br>Creatinine > ULN and > than baseline but ≤ 1.5x baseline | • Continue I-O therapy per protocol<br>• Monitor creatinine weekly | <u>If returns to baseline</u>:<br>•Resume routine creatinine monitoring per protocol<br><u>If worsens:</u><br>•Treat as Grade 2 or 3/4 |
| **Grade 2-3**<br>Creatinine > 1.5x baseline to ≤ 6x ULN | • Delay I-O therapy per protocol<br>• Monitor creatinine every 2-3 days<br>• 0.5 to 1.0 mg/kg/day methylprednisolone IV or oral equivalent<br>• Consider renal biopsy with nephrology consult | <u>If returns to Grade 1:</u><br>•Taper steroids over at least 1 month, consider prophylactic antibiotics for opportunistic infections, and resume I-O therapy and routine creatinine monitoring per protocol<br><br><u>If elevations persist > 7 days or worsen:</u><br>•Treat as Grade 4 |
| **Grade 4**<br>Creatinine > 6x ULN | • Discontinue I-O therapy per protocol<br>• Monitor creatinine daily<br>• 1.0-2.0 mg/kg/day methylprednisolone IV or IV equivalent<br>• Consult nephrologist<br>• Consider renal biopsy | <u>If returns to Grade 1</u>: Taper steroids over at least 1 month and add prophylactic antibiotics for opportunistic infections |

Patients on IV steroids may be switched to an equivalent dose of oral corticosteroids (e.g. prednisone) at start of tapering or earlier, once sustained clinical improvement is observed. Lower bioavailability of oral corticosteroids should be taken into account when switching to the equivalent dose of oral corticosteroids.

Updated 05-Jul-2016

## Pulmonary Adverse Event Management Algorithm

Rule out non-inflammatory causes. If non-inflammatory cause, treat accordingly and continue I-O therapy. Evaluate with imaging and pulmonary consultation.

| Grade of Pneumonitis (NCI CTCAE v4) | Management | Follow-up |
|---|---|---|
| **Grade 1** Radiographic changes only | • Consider delay of I-O therapy<br>• Monitor for symptoms every 2-3 days<br>• Consider Pulmonary and ID consults | • Re-image at least every 3 weeks<br>If worsens:<br>• Treat as Grade 2 or 3-4 |
| **Grade 2** Mild to moderate new symptoms | • Delay I-O therapy per protocol<br>• Pulmonary and ID consults<br>• Monitor symptoms daily, consider hospitalization<br>• 1.0 mg/kg/day methyl-prednisolone IV or oral equivalent<br>• Consider bronchoscopy, lung biopsy | • Re-image every 1-3 days<br>If improves:<br>• When symptoms return to near baseline, taper steroids over at least 1 month and then resume I-O therapy per protocol and consider prophylactic antibiotics<br>If not improving after 2 weeks or worsening:<br>• Treat as Grade 3-4 |
| **Grade 3-4** Severe new symptoms; New/worsening hypoxia; Life-threatening | • Discontinue I-O therapy per protocol<br>• Hospitalize<br>• Pulmonary and ID consults<br>• 2-4 mg/kg/day methylprednisolone IV or IV equivalent<br>• Add prophylactic antibiotics for opportunistic infections<br>• Consider bronchoscopy, lung biopsy | If improves to baseline:<br>• Taper steroids over at least 6 weeks<br>If not improving after 48 hours or worsening:<br>• Add additional immunosuppression |

Patients on IV steroids may be switched to an equivalent dose of oral corticosteroids (e.g. prednisone) at start of tapering or earlier, once sustained clinical improvement is observed. Lower bioavailability of oral corticosteroids should be taken into account when switching to the equivalent dose of oral corticosteroids.

Updated 05-Jul-2016

# Hepatic Adverse Event Management Algorithm

Rule out non-inflammatory causes. If non-inflammatory cause, treat accordingly and continue I-O therapy. Consider imaging for obstruction.

| Grade of Liver Test Elevation (NCI CTCAE v4) | Management | Follow-up |
|---|---|---|
| **Grade 1**<br>AST or ALT > ULN to 3.0 x ULN and/or T. bili > ULN to 1.5 x ULN | • Continue I-O therapy per protocol | • Continue LFT monitoring per protocol<br>If worsens:<br>• Treat as Grade 2 or 3-4 |
| **Grade 2**<br>AST or ALT > 3.0 to ≤ 5 x ULN and/or T. bili > 1.5 to ≤ 3 x ULN | • Delay I-O therapy per protocol<br>• Increase frequency of monitoring to every 3 days | If returns to baseline:<br>•Resume routine monitoring, resume I-O therapy per protocol<br>If elevations persist > 5-7 days or worsen :<br>• 0.5-1 mg/kg/day methylprednisolone or oral equivalent and when LFT returns to grade 1 or baseline, taper steroids over at least 1 month, consider prophylactic antibiotics for opportunistic infections, and resume I-O therapy per protocol |
| **Grade 3-4**<br>AST or ALT > 5 x ULN or T.bili >3 x ULN | • Discontinue I-O therapy*<br>• Increase frequency of monitoring to every 1-2 days<br>• 1.0 to 2.0 mg/kg/day methylprednisolone IV or IV equivalent**<br>• Add prophylactic antibiotics for opportunistic infections<br>• Consult gastroenterologist | If returns to grade 2:<br>• Taper steroids over at least 1 month<br>If does not improve in >3-5 days, worsens or rebounds:<br>• Add mycophenolate mofetil 1 g BID<br>• If no response within an additional 3-5 days, consider other immunosuppressants per local guidelines |

Patients on IV steroids may be switched to an equivalent dose of oral corticosteroids (e.g. prednisone) at start of tapering or earlier, once sustained clinical improvement is observed. Lower bioavailability of oral corticosteroids should be taken into account when switching to the equivalent dose of oral corticosteroids.

*I-O therapy may be delayed rather than discontinued if AST/ALT ≤ 8 x ULN or T.bili ≤ 5 x ULN.

**The recommended starting dose for grade 4 hepatitis is 2 mg/kg/day methylprednisolone IV.

Updated 05-Jul-2016

## Endocrinopathy Management Algorithm

Rule out non-inflammatory causes. If non-inflammatory cause, treat accordingly and continue I-O therapy. Consider visual field testing, endocrinology consultation, and imaging.

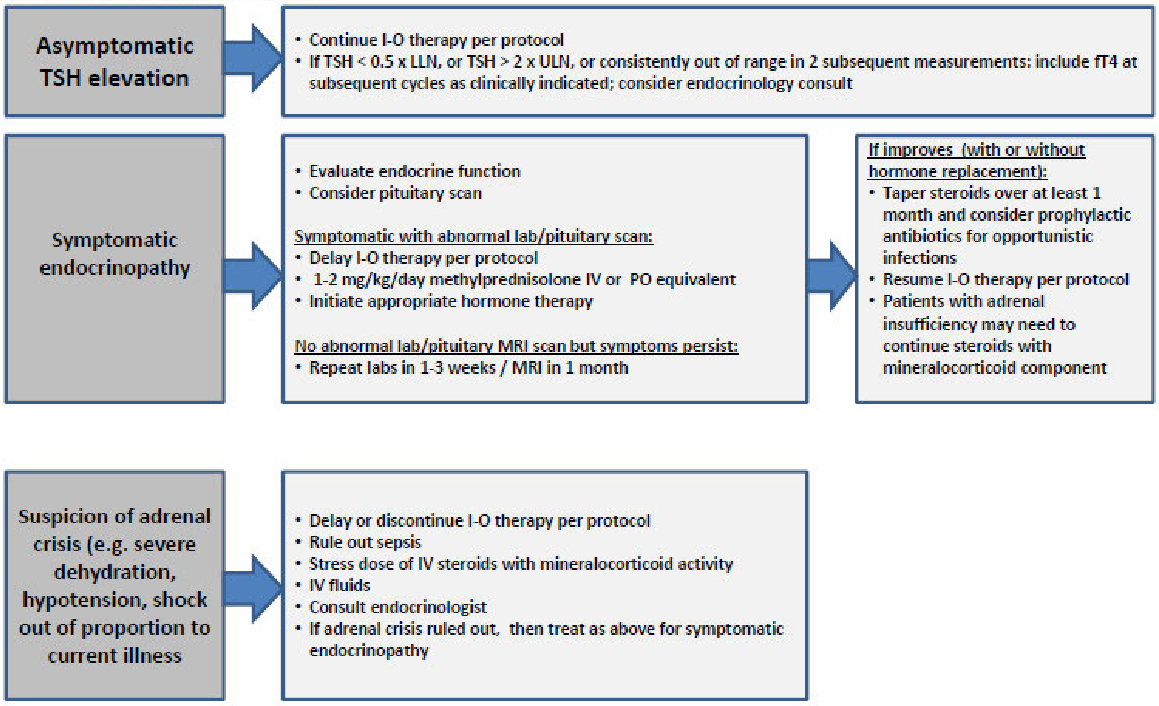


Patients on IV steroids may be switched to an equivalent dose of oral corticosteroids (e.g. prednisone) at start of tapering or earlier, once sustained clinical improvement is observed. Lower bioavailability of oral corticosteroids should be taken into account when switching to the equivalent dose of oral corticosteroids.

# Skin Adverse Event Management Algorithm

Rule out non-inflammatory causes. If non-inflammatory cause, treat accordingly and continue I-O therapy.

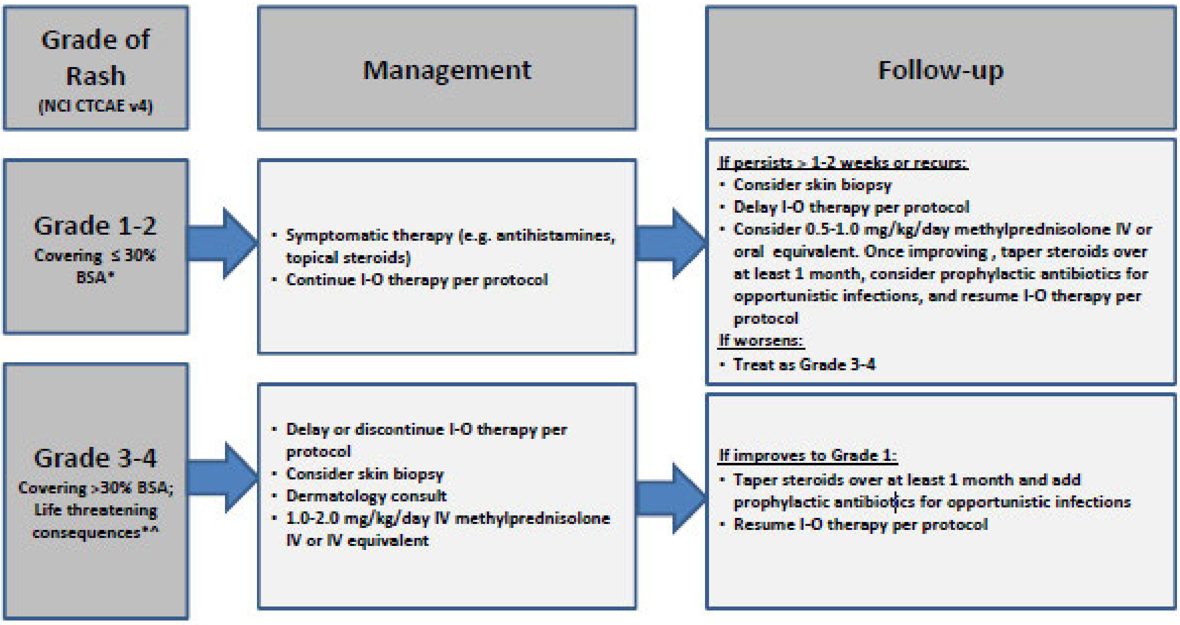


Patients on IV steroids may be switched to an equivalent dose of oral corticosteroids (e.g. prednisone) at start of tapering or earlier, once sustained clinical improvement is observed. Lower bioavailability of oral corticosteroids should be taken into account when switching to the equivalent dose of oral corticosteroids.

*Refer to NCI CTCAE v4 for term-specific grading criteria.

^If SJS/TEN is suspected, withhold I-O therapy and refer patient for specialized care for assessment and treatment. If SJS or TEN is diagnosed, permanently discontinue I-O therapy.

# Neurological Adverse Event Management Algorithm

Rule out non-inflammatory causes. If non-inflammatory cause, treat accordingly and continue I-O therapy.

| Grade of Neurological Toxicity (NCI CTCAE v4) | Management | Follow-up |
|---|---|---|
| **Grade 1**<br>Asymptomatic or mild symptoms; Intervention not indicated | • Continue I-O therapy per protocol | Continue to monitor the patient.<br><u>If worsens:</u><br>• Treat as Grade 2 or 3-4 |
| **Grade 2**<br>Moderate symptoms; Limiting instrumental ADL | • Delay I-O therapy per protocol<br>• Treat symptoms per local guidelines<br>• Consider 0.5 to 1.0 mg/kg/day methylprednisolone IV or PO equivalent | <u>If improves to baseline</u>:<br>•Resume I-O therapy per protocol when improved to baseline<br><u>If worsens</u>:<br>• Treat as Grade 3-4 |
| **Grade 3-4**<br>Severe symptoms; Limiting self-care ADL; Life-threatening | • Discontinue I-O therapy per protocol<br>• Obtain neurology consult<br>• Treat symptoms per local guidelines<br>• 1.0-2.0 mg/kg/day IV methylprednisolone IV or IV equivalent<br>• Add prophylactic antibiotics for opportunistic infections | <u>If improves to Grade 2</u>:<br>• Taper steroids over at least 1 month<br><br><u>If worsens or atypical presentation:</u><br>• Consider IVIG or other immunosuppressive therapies per local guidelines |

Patients on IV steroids may be switched to an equivalent dose of oral corticosteroids (e.g. prednisone) at start of tapering or earlier, once sustained clinical improvement is observed. Lower bioavailability of oral corticosteroids should be taken into account when switching to the equivalent dose of oral corticosteroids.

# Appendix D: immunoscore

**Table 1 Current Immunoscore procedure and reagents**

| Procedure | Current recommended steps |
|---|---|
| Tumor selection | Block which is the most infiltrated by the immune cells and containing the core of the tumor (CT) and the invasive margin (IM) |
| Sample preparation | 2 paraffin sections of 4-microns of the tumor block deposited in deionized water on Superfrost-plus slides |
| Immuno-histochemistry (IHC) | 2 single stainings using IVD certified antibodies |
| Antigen retrieval | CC1 tris-based buffer pH8 |
| Primary antibody | CD3 (2GV6, Ventana) and CD8 (C8/144, Dako) |
| Primary antibody diluant | K 004 (Clinisciences) for CD8 |
| Secondary reagents | Ultraview TM DAB (Ventana) |
| Counterstaining | Hematoxillin II (Ventana) |
| Autostrainer | Benchmark XT (Ventana) |
| Scanner | NanoZoomer 2.0-HT (Hammamatsu) |
| Digital pathology | Architect XD software (Definiens) |
| Immunoscore quantification | Immunoscore Plug-in (INSERM / AP-HP) |

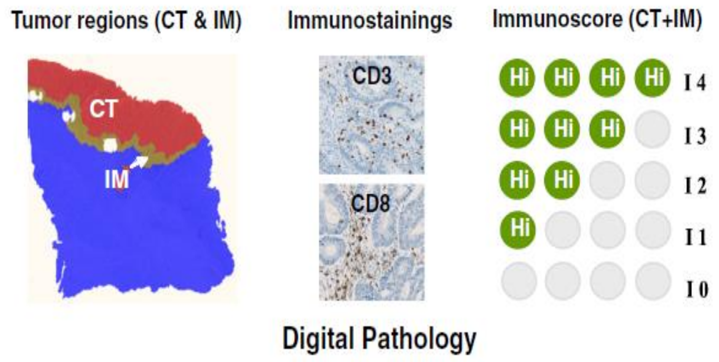


**Figure 1** Immunoscore definition and method.

**CT = core of tumor; IM = invasive margin;**
Proposed cutoff values in cells/mm2: CD3CT 250; CD3IM 640; CD45ROCT 60, and CD45ROIM 190.

## 12. REFERENCES

1. Galon J, Costes A, Sanchez-Cabo F, et al: Type, density, and location of immune cells within human colorectal tumors predict clinical outcome. Science 313:1960-4, 2006
2. Le DT, Uram JN, Wang H, et al: PD-1 Blockade in Tumors with Mismatch-Repair Deficiency. N Engl J Med 372:2509-20, 2015
3. Pages F, Berger A, Camus M, et al: Effector memory T cells, early metastasis, and survival in colorectal cancer. N Engl J Med 353:2654-66, 2005
4. Topalian SL, Hodi FS, Brahmer JR, et al: Safety, activity, and immune correlates of anti-PD-1 antibody in cancer. N Engl J Med 366:2443-54, 2012
5. Overman MJ KS, McDermott RS, et al. : Nivolumab +/- ipilimumab in treatment of patients with metastatic colorectal cancer with and without high microsatellite instability: CheckMate-142 interim results. ASCO Meeting Abstracts 34:3501, 2016. ,
6. Mlecnik B, Bindea G, Kirilovsky A, et al: The tumor microenvironment and Immunoscore are critical determinants of dissemination to distant metastasis. Sci Transl Med 8:327ra26, 2016
7. Laghi L, Malesci A: Microsatellite instability and therapeutic consequences in colorectal cancer. Dig Dis 30:304-9, 2012
8. Zelenay S, van der Veen AG, Bottcher JP, et al: Cyclooxygenase-Dependent Tumor Growth through Evasion of Immunity. Cell 162:1257-70, 2015
9. Wong JL, Obermajer N, Odunsi K, et al: Synergistic COX2 Induction by IFNγ and TNFα Self-Limits Type-1 Immunity in the Human Tumor Microenvironment. Cancer Immunol Res 4:303-11, 2016
10. Andre T, Boni C, Navarro M, et al: Improved overall survival with oxaliplatin, fluorouracil, and leucovorin as adjuvant treatment in stage II or III colon cancer in the MOSAIC trial. J Clin Oncol 27:3109-16, 2009
11. Andre T, de Gramont A, Vernerey D, et al: Adjuvant Fluorouracil, Leucovorin, and Oxaliplatin in Stage II to III Colon Cancer: Updated 10-Year Survival and Outcomes According to BRAF Mutation and Mismatch Repair Status of the MOSAIC Study. J Clin Oncol 33:4176-87, 2015
12. Rizvi N GS, Goldman J, Hellmann MD, Chow LQ, Juergens R, Borghaei H, Brahmer J, Shen Y, Harbison C, Nathan F, Ready NE, Antonia SJ. : Safety and efficacy of first-line nivolumab and ipilimumab in non-small cell lung cancer. 16th world conference on lung cancer., 2015
13. Yothers G, O'Connell MJ, Allegra CJ, et al: Oxaliplatin as adjuvant therapy for colon cancer: updated results of NSABP C-07 trial, including survival and subset analyses. J Clin Oncol 29:3768-74, 2011
14. Schmoll HJ, Twelves C, Sun W, et al: Effect of adjuvant capecitabine or fluorouracil, with or without oxaliplatin, on survival outcomes in stage III colon cancer and the effect of oxaliplatin on post-relapse survival: a pooled analysis of individual patient data from four randomised controlled trials. Lancet Oncol 15:1481-92, 2014
15. Mlecnik B, Bindea G, Angell HK, et al: Integrative Analyses of Colorectal Cancer Show Immunoscore Is a Stronger Predictor of Patient Survival Than Microsatellite Instability. Immunity 44:698-711, 2016
16. Joyce JA, Fearon DT: T cell exclusion, immune privilege, and the tumor microenvironment. Science 348:74-80, 2015
17. Wang D, Xia D, Dubois RN: The Crosstalk of PTGS2 and EGF Signaling Pathways in Colorectal Cancer. Cancers (Basel) 3:3894-908, 2011
18. Obermajer N, Muthuswamy R, Lesnock J, et al: Positive feedback between PGE2 and COX2 redirects the differentiation of human dendritic cells toward stable myeloid-derived suppressor cells. Blood 118:5498-505, 2011

19. Obermajer N, Muthuswamy R, Odunsi K, et al: PGE(2)-induced CXCL12 production and CXCR4 expression controls the accumulation of human MDSCs in ovarian cancer environment. Cancer Res 71:7463-70, 2011
20. Bronte V, Serafini P, Mazzoni A, et al: L-arginine metabolism in myeloid cells controls T-lymphocyte functions. Trends Immunol 24:302-6, 2003
21. Mazzoni A, Bronte V, Visintin A, et al: Myeloid suppressor lines inhibit T cell responses by an NO-dependent mechanism. J Immunol 168:689-95, 2002
22. Sinha P, Clements VK, Bunt SK, et al: Cross-talk between myeloid-derived suppressor cells and macrophages subverts tumor immunity toward a type 2 response. J Immunol 179:977-83, 2007
23. Li P, Wu H, Zhang H, et al: Aspirin use after diagnosis but not prediagnosis improves established colorectal cancer survival: a meta-analysis. Gut 64:1419-25, 2015
24. Bains SJ, Mahic M, Myklebust TA, et al: Aspirin As Secondary Prevention in Patients With Colorectal Cancer: An Unselected Population-Based Study. J Clin Oncol, 2016
25. Pardoll DM: The blockade of immune checkpoints in cancer immunotherapy. Nat Rev Cancer 12:252-64, 2012
26. Hamid O, Carvajal RD: Anti-programmed death-1 and anti-programmed death-ligand 1 antibodies in cancer therapy. Expert Opin Biol Ther 13:847-61, 2013
27. Brahmer JR, Drake CG, Wollner I, et al: Phase I study of single-agent anti-programmed death-1 (MDX-1106) in refractory solid tumors: safety, clinical activity, pharmacodynamics, and immunologic correlates. J Clin Oncol 28:3167-75, 2010
28. Curran MA, Montalvo W, Yagita H, et al: PD-1 and CTLA-4 combination blockade expands infiltrating T cells and reduces regulatory T and myeloid cells within B16 melanoma tumors. Proc Natl Acad Sci U S A 107:4275-80, 2010
29. Antonia S, Goldberg SB, Balmanoukian A, et al: Safety and antitumour activity of durvalumab plus tremelimumab in non-small cell lung cancer: a multicentre, phase 1b study. Lancet Oncol 17:299-308, 2016
30. Wolchok JD, Kluger H, Callahan MK, et al: Nivolumab plus ipilimumab in advanced melanoma. N Engl J Med 369:122-33, 2013
31. Voron T, Colussi O, Marcheteau E, et al: VEGF-A modulates expression of inhibitory checkpoints on CD8+ T cells in tumors. J Exp Med 212:139-48, 2015
32. Sato T, Stange DE, Ferrante M, et al: Long-term expansion of epithelial organoids from human colon, adenoma, adenocarcinoma, and Barrett's epithelium. Gastroenterology 141:1762-72, 2011
