# Supplementary material for: Neoadjuvant immunotherapy in mismatch-repair-proficient colon cancers
Source: Nature. 2025 Oct 20;648(8094):726–35. doi: 10.1038/s41586-025-09679-4 (PMC12711568; doi:10.1038/s41586-025-09679-4)
Supplement: Supplementary file 2 — Reporting Summary [file 41586_2025_9679_MOESM2_ESM.pdf]

nature portfolio

Corresponding author(s): M. Chalabi

Last updated by author(s): Sep 16, 2025

Reporting Summary

Nature Portfolio wishes to improve the reproducibility of the work that we publish. This form provides structure for consistency and transparency in reporting. For further information on Nature Portfolio policies, see our Editorial Policies and the Editorial Policy Checklist.

Statistics

For all statistical analyses, confirm that the following items are present in the figure legend, table legend, main text, or Methods section.

n/a	Confirmed	
☐	☒	The exact sample size (*n*) for each experimental group/condition, given as a discrete number and unit of measurement
☐	☒	A statement on whether measurements were taken from distinct samples or whether the same sample was measured repeatedly
☐	☒	The statistical test(s) used AND whether they are one- or two-sided<br>*Only common tests should be described solely by name; describe more complex techniques in the Methods section.*
☐	☒	A description of all covariates tested
☐	☒	A description of any assumptions or corrections, such as tests of normality and adjustment for multiple comparisons
☐	☒	A full description of the statistical parameters including central tendency (e.g. means) or other basic estimates (e.g. regression coefficient) AND variation (e.g. standard deviation) or associated estimates of uncertainty (e.g. confidence intervals)
☐	☒	For null hypothesis testing, the test statistic (e.g. *F*, *t*, *r*) with confidence intervals, effect sizes, degrees of freedom and *P* value noted<br>*Give P values as exact values whenever suitable.*
☒	☐	For Bayesian analysis, information on the choice of priors and Markov chain Monte Carlo settings
☒	☐	For hierarchical and complex designs, identification of the appropriate level for tests and full reporting of outcomes
☐	☒	Estimates of effect sizes (e.g. Cohen's *d*, Pearson's *r*), indicating how they were calculated

Our web collection on statistics for biologists contains articles on many of the points above.

Software and code

Policy information about availability of computer code

Data collection	Clinical data from NICHE: TENALEA clinical trial data management system
Data analysis	- Analysis of clinical data: R v.4.3.0 , R-studio (build 561), packages: arsenal (v3.6.3), survival (v3.6-4), survminer (v0.4.9)<br>- Analysis of DNA, RNA, IMC data: R v4.2.3, R-studio (build 513), tidyverse (v2.0). ggplot2 (v3.4.2), ggpubr (v0.6.0) pheatmap (v1.0.12).<br>- Analysis of circulating tumor DNA (ctDNA) data: R v4.3.1, packages: stats (v4.3.1), mosaic (v1.9.1)<br>- Imaging Mass Cytometry (IMC): Fluidigm mcd viewer (v1.0.560.6), CellProfiler (v4.2.1), Cytosplore (v2.3.1).<br>- Immunohistochemistry (IHC): HALO v4.0.5107.357 (Indica Labs) with the Indica Labs Multiplex IHC v3.0.3 analysis algorithm<br>- DNA (whole-exome) sequencing: Sarek pipeline (v3.1.2), bwa (v.0.7.17), MarkDuplicates (v4.3), GATK BaseRecalibrator (v4.3), Strelka2 (v2.9.10), snpeff (v5.1), ensemblvep (v106.1), vcf2maf (v1.6.22), OncoKB annotator (v3.4.1), ASCAT (v3.0).<br>- RNA sequencing: Hisat2 (v2.2.1), gensum (https://github.com/NKI-GCF/gensum), DESeq2 (v.1.38.3), enrichR (v3.2), GSVA (v1.46), geom_smooth (v3.4.2)<br>- scRNA sequencing and TCRsequencing: R v4.2.3, Cell Ranger (v7.1.0); Seurat (v5.2.1); scRepertoire (v2.3.2)

For manuscripts utilizing custom algorithms or software that are central to the research but not yet described in published literature, software must be made available to editors and reviewers. We strongly encourage code deposition in a community repository (e.g. GitHub). See the Nature Portfolio guidelines for submitting code & software for further information.

Data

Policy information about availability of data

All manuscripts must include a data availability statement. This statement should provide the following information, where applicable:

- Accession codes, unique identifiers, or web links for publicly available datasets
- A description of any restrictions on data availability
- For clinical datasets or third party data, please ensure that the statement adheres to our policy

DNA and RNA sequencing data for the NICHE study is deposited in the European Genome-phenome Archive (EGA) under accession number EGAS50000000856. Data is under controlled access according to consent provided by the patients whose samples are used and according to GDPR. Data will be made available for academic use only upon reasonable request and within the confinements of the informed consent and the European Data Protection Regulation. Requests should include project descriptions describing the research goal, privacy, governance and intended use of data, and can be done through https://ega.nki.nl/, contacting repository@nki.nl. Requests will be reviewed by the institutional review board of the Netherlands Cancer Institute (NKI) and require signing of a data access agreement with the NKI after approval.

Clinical data from The Cancer Genome Atlas (TCGA) Research Network were obtained from the Clinical data resource Liu et al. 2018 (DOI: 10.1016/j.cell.2018.02.052). TCGA mutational status for CRC was obtained from analysis by Grasso et al. 2018, available as supplementary material (DOI: 10.1158/2159-8290.CD-17-1327). RNAseq data is openly available and was obtained from cBioportal (https://www.cbioportal.org), with accession code coadread_tcga_pan_can_atlas_2018. Data for the AC-ICAM CC cohort is openly available and was downloaded from cBioportal (https://www.cbioportal.org), with accession code coad_silu_2022.

Research involving human participants, their data, or biological material

Policy information about studies with human participants or human data. See also policy information about sex, gender (identity/presentation), and sexual orientation and race, ethnicity and racism.

Reporting on sex and gender

Patients were included regardless of sex and/or gender. The study protocol did not include any pre-specified sex- or gender-based analyses and no exploratory sex- or gender-based analyses were performed. The biological sex of each patient was collected from the national Personal Records Database (BRP) and used in Table 1 with baseline characteristics of the cohort; the enrolled cohort included 15 females and 18 males.

Reporting on race, ethnicity, or other socially relevant groupings

Race, ethnicity and other socially relevant groupings were not considered in the study design or the analysis and were not reported in the manuscript.

Population characteristics

Patients diagnosed with MMR proficient, previously untreated non-metastatic colon adenocarcinoma who were at least 18 years or older were eligible. Patients were included irrespective of sex and/or gender (included: 55% male, 45% female). There was no age limit and the median age of included patients was 62, ranging from 44 to 77.

All patients had a World Health Organization performance status of 0 or 1 (included: 94% WHO 0 and 6% WHO 1) and adequate hematologic and end-organ function. Key exclusion criteria included signs of obstruction or perforation, prior immunotherapy, active autoimmune disease requiring systemic immunosuppressive treatment, and active concurrent cancer. Baseline characteristics of included patients are presented in Table 1 of the manuscript.

Recruitment

Patients that presented with an initial diagnosis of non-metastatic, resectable colon adenocarcinoma, either at our center or referred from another center, who were potentially eligible for this study were informed about the standard of care and the possibility of participation in the current study and, if relevant, any other studies they were eligible for. Patients deemed eligible for the NICHE study were informed about the aims of this study, the study procedures and study treatment as well as possible adverse events and other hazards to which they may be exposed in case of participation. Interested patients were provided the patient information folder and consent form containing extensive details on the study and study procedures, after which they were given sufficient time to read the materials and decide on participation. Possible inclusion bias may have arisen from the following situations: a) patients with extensive disease (e.g. T4 status) for whom induction or neoadjuvant treatment was deemed necessary were not included in this study since insufficient data were available to justify omission of chemotherapy as an induction treatment). b) Only fit patients with WHO performance status 0 or 1 and no significant comorbidities were included, and while part of the inclusion criteria this is a source of bias in many clinical trials.

Ethics oversight

The study protocol was approved by the institutional review board of the NKI (sponsor) and by the local ethics boards of the participating centers: OLVG and Spaarne Gasthuis. The study was conducted in accordance with the International Conference on Harmonization Guideline for Good Clinical Practice and the principles of the Declaration of Helsinki. All patients provided written informed consent.

Note that full information on the approval of the study protocol must also be provided in the manuscript.

Field-specific reporting

Please select the one below that is the best fit for your research. If you are not sure, read the appropriate sections before making your selection.

☒ Life sciences ☐ Behavioural & social sciences ☐ Ecological, evolutionary & environmental sciences

For a reference copy of the document with all sections, see nature.com/documents/nr-reporting-summary-flat.pdf

Life sciences study design

All studies must disclose on these points even when the disclosure is negative.

Sample size	- The NICHE study is an exploratory, hypothesis-generating study and no formal sample size calculation was performed. The study aimed to treat a total of 30 patients with pMMR colon cancer.<br>- Genomic data from the colorectal cancer cohort from The Cancer Genome Atlas Research Network was obtained (n=592) and 287 patients with colon cancer, pathological stage I-III disease and pMMR annotations without POLE mutations were included. RNAseq data was available for 276 of these patients.<br>- Genomic data from the colon cancer cohort from the atlas and compass of immune-colon cancer-microbiome interactions (AC-ICAM) was obtained (n=281) and 173 patients with pathological stage I-III disease, pMMR annotations and without POLE mutations were included. RNAseq data was available for all 173 patients.<br>The number of external datasets was determined based on availability of openly accessible studies in cBioportal, with matched RNA and DNA data for early stage pMMR colon cancer samples. Addition of two large external studies was considered sufficient to compare the proportion of genetic alterations and investigate gene expression changes associated with TP53 alterations.
Data exclusions	All enrolled patients in the NICHE study who received at least 1 cycle of study medication were included in the safety analyses (n=33). Among all treated patients, the patients who met all inclusion criteria at baseline were included in efficacy and translational analyses (n=31).<br><br>For external TCGA and AC-ICAM cohorts, patients with colon cancer, Stage I-III disease, MSS annotations and without POLE mutations, with DNA or RNA data available were included.
Replication	Replication is not applicable for clinical data, because this study included patients who received neoadjuvant treatment and then underwent surgery, which can only be performed once for an individual. Translational experiments on human samples were not replicated due to limited material.
Randomization	All patients in NICHE received nivolumab 3 mg/kg on days 1 and 15 plus ipilimumab on day 1. In addition, patients were randomized 1:1 to receive nivolumab/ipilimumab with or without oral celecoxib 200 mg once daily until the day prior to surgery.
Blinding	Tumour microenvironment subtype classification based on Bagaev et al. signatures was performed blinded to response labels, aided by and distributions of ssGSEA scores for immune and stromal signatures and hierarchical clustering of signature Z-scores. Other experiments were not performed blinded to group allocation, but this was not relevant due to the automation of data collection and analysis across samples.

Reporting for specific materials, systems and methods

We require information from authors about some types of materials, experimental systems and methods used in many studies. Here, indicate whether each material, system or method listed is relevant to your study. If you are not sure if a list item applies to your research, read the appropriate section before selecting a response.

Materials & experimental systems

n/a	Involved in the study	
☐	☒	Antibodies
☒	☐	Eukaryotic cell lines
☒	☐	Palaeontology and archaeology
☒	☐	Animals and other organisms
☐	☒	Clinical data
☒	☐	Dual use research of concern
☒	☐	Plants

Methods

n/a	Involved in the study	
☒	☐	ChIP-seq
☒	☐	Flow cytometry
☒	☐	MRI-based neuroimaging

Antibodies

Antibodies used	Immunohistochemistry (IHC):<br>• MLH1, Ready-to-Use (=undiluted), M1, 6472966001, LotNo: G07286, G09887, Roche Diagnostics, Tucson, AZ, United States of America<br>• MSH2, Ready-to-Use (=undiluted), G219-1129, 5269270001, LotNo 2017: 161008B, 1616008C, V0000776, LotNo 2018: V0001229, LotNo 2019: V0001244, V0001272, V0001273, f06034, F1125, f17180. LotNo 2020: f15301, f19415, f29067. 2021 LotNo 2021: g07854, g17138, g32360, h05779, h03163, h05779, h12642, Roche Diagnostics, Tucson, AZ, United States of America<br>• MSH6, 1/50 dilution, EP49, AC-0047, LotNo: EN020910,EN072101, EN100602, 19060401, 20021305, Abcam, Cambridge, United Kingdom<br>• PMS2 (used in 2017), 1/20 dilution, clone EP51, M3647, LotNo: 10112572, 10122891, Agilent/DAKO, Santa Clara, California, United States of America<br>• PMS2 (used in 2018 and thereafter), Ready-to-Use (=undiluted), clone EPR3947, 7604531 LotNo 2018: V0000986, V0001061, V0001217, V0001251, V0001198, V0001253, LotNo 2019: V0001253, F01588M, F060406M, F08243M, F09828M, F05846M, F07456M, LotNo 2020: F16520, F19021, F25056, G03551, G10164 2021: G21216, G10165, G05452, G32555, G33341, G19245, H000178, Roche Diagnostics, Tucson, AZ, United States of America<br><br>Imaging Mass Cytometry (IMC):

IMC antibodies use custom carrier-free formulations with metal conjugations ordered with the respective companies.
• CD4 clone EPR6855, 1/100 dilution, metal: 145 Nd, LotNo: 1014578-6, Abcam, Cambridge, United Kingdom, catalog number ab181724
• TCRgd clone H41, 1/25 dilution, metal: 148 Nd, LotNo: D3021, Santa Cruz biotechnology, Dallas, United states, catalog number sc-100289
• Anti-rabbit IgG, polyclonal, 1/100 dilution, metal: 145 Nd, LotNo: GR3215731-15, Abcam, Cambridge, United Kingdom, catalog number ab6701
• Anti-mouse IgG, polyclonal, 1/100 dilution, metal: 148 Nd, LotNo: GR3300461-1, Abcam, Cambridge, United Kingdom, catalog number ab6708
• CD8a clone D8A8Y, 1/50 dilution, metal: 146 Nd, LotNo: 2, Cell signaling technology, Danvers, United states, catalog number 81575SF
• PD-1 clone D4W2J, 1/50 dilution, metal: 160 Gd, LotNo: 1, Cell signaling technology, Danvers, United states, catalog number 63815SF
• ICOS clone D1K2T(tm), 1/50 dilution, metal: 161 Dy, LotNo: 4, Cell signaling technology, Danvers, United states, catalog number 39740SF
• CD204 clone J5HTR3, 1/50 dilution, metal: 164 Dy, LotNo: 2518439, Thermo Fisher Scientific, Waltham, United States, catalog number 14-9054-82
• CD103 clone EPR4166(2), 1/50 dilution, metal: 168 Er, LotNo: GR3399209-2, Abcam, Cambridge, United Kingdom, catalog number ab221210
• Tbet clone 4B10, 1/50 dilution, metal: 170 Er, LotNo: B298378, Biolegend, San Diego, United States, catalog number 644825
• Caspase clone D4V4B, 1/50 dilution, metal: 172 Yb, LotNo: 25, Cell signaling technology, Danvers, United states, catalog number 74860SF
• CD163 clone D6U1J, 1/50 dilution, metal: 173 Yb, LotNo: 1, Cell signaling technology, Danvers, United states, catalog number 25121SF
• HLA-DR clone TAL 1B5, 1/100 dilution, metal: 141 Pr, LotNo: GR3424852-2, Abcam, Cambridge, United Kingdom, catalog number ab176408
• CD11b clone D6X1N, 1/100 dilution, metal: 144 Nd, LotNo: 1, Cell signaling technology, Danvers, United states, catalog number 23743SF
• Granzyme B clone D6E9W, 1/100 dilution, metal: 150 Nd, LotNo: 7, Cell signaling technology, Danvers, United states, catalog number 79903SF
• CD138 clone 5A1E, 1/100 dilution, metal: 155 Gd, LotNo: 1, Cell signaling technology, Danvers, United states, catalog number 94530SF
• CD39 clone EPR20627, 1/100 dilution, metal: 157 Gd, LotNo: GR3274485-6, Abcam, Cambridge, United Kingdom, catalog number ab236038
• VISTA clone D1L2G(TM), 1/100 dilution, metal: 158 Gd, LotNo: 7, Cell signaling technology, Danvers, United states, catalog number 56548SF
• CD14 clone D7A2T, 1/100 dilution, metal: 163 Dy, LotNo: 2, Cell signaling technology, Danvers, United states, catalog number 43878SF
• CD56 clone E7X9M, 1/100 dilution, metal: 167 Er, LotNo: 2, Cell signaling technology, Danvers, United states, catalog number 88856SF
• CD7 clone EPR4242, 1/100 dilution, metal: 174 Yb, LotNo: GR3424737-2, Abcam, Cambridge, United Kingdom, catalog number ab230834
• CD11c clone EP1347Y, 1/100 dilution, metal: 176 Yb, LotNo: GR3357092-17, Abcam, Cambridge, United Kingdom, catalog number ab216655
• CD45 clone D9M8I, 1/50 dilution, metal: 149 Sm, LotNo: 12, Cell signaling technology, Danvers, United states, catalog number 47937SF
• CD3 clone EP449E, 1/50 dilution, metal: 153 Eu, LotNo: GR3418069-6, Abcam, Cambridge, United Kingdom, catalog number ab271850
• PD-L1 clone E1L3N(R), 1/50 dilution, metal: 156 Gd, LotNo: 2, Cell signaling technology, Danvers, United states, catalog number 85164SF
• FOXP3 clone D608R, 1/50 dilution, metal: 159 Tb, LotNo: 2, Cell signaling technology, Danvers, United states, catalog number 72338SF
• CD27 clone EPR8569, 1/50 dilution, metal: 175 Lu, LotNo: GR3446729-2, Abcam, Cambridge, United Kingdom, catalog number ab192336
• Vimentin clone D21H3, 1/50 dilution, metal: 194 Pt, LotNo: 1, Cell signaling technology, Danvers, United states, catalog number 46173SF
• Keratin clone C11, 1/50 dilution, metal: 198 Pt, LotNo: 2, Cell signaling technology, Danvers, United states, catalog number 17171SF
• Keratin clone AE1/AE3, 1/50 dilution, metal: 198 Pt, LotNo: B302316, Biolegend, San Diego, United states, catalog number 914204
• TGFb clone TB21, 1/100 dilution, metal: 89Y, LotNo: 157850, Cell signaling technology, Danvers, United states, catalog number MA5-16949
• CD20 clone H1, 1/100 dilution, metal: 142 Nd, LotNo: 1209781, BD Biosciences, Franklin Lakes, United states, catalog number 555677
• CD68 clone D4B9C, 1/100 dilution, metal: 143 Nd, LotNo: 2, Cell signaling technology, Danvers, United states, catalog number 26042SF
• CD31 clone 89C2, 1/100 dilution, metal: 147 Sm, LotNo: 1, Cell signaling technology, Danvers, United states, catalog number 85873SF
• CD57 clone HNK-1 / Leu-7, 1/100 dilution, metal: 151 Eu, LotNo: GR3373313, Abcam, Cambridge, United Kingdom, catalog number ab212403
• Ki-67 clone 8D5, 1/100 dilution, metal: 152 Sm, LotNo: 11, Cell signaling technology, Danvers, United states, catalog number 62548SF
• IgG1 clone EPR4417, 1/100 dilution, metal: 154 Sm, Abcam, Cambridge, United Kingdom, catalog number ab232544
• IDO clone D5J4E(TM) , 1/100 dilution, metal: 162 Dy, LotNo: 7, Cell signaling technology, Danvers, United states, catalog number 91473SF
• CD45RO clone UCHL1, 1/100 dilution, metal: 165 Ho, LotNo: 1, Cell signaling technology, Danvers, United states, catalog number 36282SF
• D2-40 clone D2-40, 1/100 dilution, metal: 166 Er, LotNo: B316467,

Biolegend, San Diego, United states, catalog number 916606
• CD38 clone EPR4106, 1/100 dilution, metal: 169 Tm, LotNo: GR3378690-1, Abcam, Cambridge, United Kingdom, catalog number ab226034
• CD15 clone MC480, 1/100 dilution, metal: 171 Yb, LotNo: 5,
Cell signaling technology, Danvers, United states, catalog number 74180SF
• Bcatenin clone D10A8, 1/100 dilution, metal: 196 Pt, LotNo: 1, Cell signaling technology, Danvers, United states, catalog number 84441SF
• Histone H3 clone D1H2, 1/50 dilution, metal: 209 Bi, LotNo: 1, Cell signaling technology, Danvers, United states, catalog number 60932SF

scRNAseq hashing and protein markers:
• TotalSeq-C anti-human hashtag antibodies (clones LNH-94 and 2M2, numbers 1–13; final concentration 1µg/mL, BioLegend), catalog numbers 394661, 394663, 394665, 394667, 394669, 394671, 394673, 394675, 394677, 394679, 328941, 394683, 394685
• anti-CD45-PerCP-Cy5.5, clone HI30, 1/50 dilution, Invitrogen, catalog number 45-0459-42
• PD-1, clone EH12-EH7, 1/1000 dilution, TotalSeq-C, BioLegend, catalog number 329902
• CD39, clone A1, 1/1000 dilution, TotalSeq-C, BioLegend, catalog number 328237
• CD137, clone 4B4-1, 1/5000 dilution, TotalSeq-C, BioLegend, catalog number 309839
• CD8, clone SK1, 1/5000 dilution, TotalSeq-C, BioLegend, catalog number 344753
• CD4, clone RPA-T4, 1/2500 dilution, TotalSeq-C, BioLegend, catalog number 300567

Validation

For MLH1, MSH2, MSH6, and PMS2, IHC protocols have been developed and validated under standard operating procedures in a certified pathology lab (EN ISO15189, M258). Each new antibody lot is validated by testing multiple dilutions and evaluation by a pathologist using a standardized method, using positive control tissues suitable for the antibody (images and protocol details available upon request). Antibodies were validated as described on the manufacturer's websites.

All IMC antibodies have been selected based on extensive validation for use in immunohistochemistry on FFPE tissue by the respective companies. All antibodies are tested in house on FFPE tonsil and colon tissue and staining patterns were compared to company datasheets and reported literature. After metal conjugation, the staining patterns of each antibody are once again validated by IHC and compared to staining prior to conjugation and reported literature. Furthermore, for each antibody, colocalization with expected other markers was confirmed by IMC.

All scRNAseq antibodies were tested for human reactivity, and validated for proteogenomics by the manufacturer. Antibodies are quality control tested by immunofluorescent staining with flow cytometric analysis and the oligomer sequences are confirmed by sequencing. Anti-CD45 clone HI30 has been validated for flow cytometry applications "This HI30 antibody has been pre-titrated and tested by flow cytometric analysis of normal human peripheral blood cells".

Roche Diagnostics antibodies are validated for human reactivity, with visual inspection of CRC and tonsil material stainings, measuring specific and background staining intensities, are free of particulate matter and turbidity. Agilent antibodies are validated by IHC with system level controls to ensure the validity of the staining procedure. Abcam antibodies undergo biophysical quality controls with liquid mass chromatography mass spectrometry and high-performance liquid chromatography and are validated for applications with positive and negative cell lines and tissues, and antibodies are often knockout validated for specificity. Cell signaling technology antibodies are validated for IHC: "Western blot analysis is performed to demonstrate specific bands of the appropriate molecular weight(s), with minimal cross-reacting bands. Paraffin-embedded cell pellets of known target expression levels are used to verify target specificity. Antibody performance is assessed in relevant mouse models of cancer. Xenografts generated from cell lines with known target expression levels help verify target specificity. Human cancer tissue arrays are used to demonstrate antibody performance over a broad spectrum of tissue types. Staining on fresh frozen tissues is performed when appropriate. Tissue sections and cell pellets are subjected to phosphatase treatment to verify target phospho-specificity. The use of blocking peptides verifies specificity and rules out Fc-mediated binding and other non-specific staining. Thorough lot testing ensures the reproducibility necessary for accurate IHC results. Dilutions and protocols are predetermined and specified; control reagents are also available". All antibodies on the Thermo Fisher Scientific website that have undergone and passed the advanced verification testing are identified with an "Advanced Verification" badge. Advanced verification is additional testing that verifies that an antibody will bind to the correct target. BD Biosciences antibodies: "Our product development process includes testing on a combination of primary cells, cell lines and/or transfectant cell models with relevant controls using multiple immunoassays to ensure biological accuracy. We also perform multiplexing with additional antibodies to interrogate antibody staining in multiple cell populations."

Clinical data

Policy information about clinical studies
All manuscripts should comply with the ICMJE guidelines for publication of clinical research and a completed CONSORT checklist must be included with all submissions.

Clinical trial registration

ClinicalTrials.gov, NCT03026140

Study protocol

The study protocol is provided as supplementary information.

Data collection

Clinical data, including data regarding adverse events, were collected from time of signing informed consent until 100 days after the last administration of study drugs. These data were collected through patient consultation in the outpatient clinic of the Netherlands Cancer Institute. Outcome data and long-term survival are collected until five years after initial diagnosis. These long-term data are collected through the outpatient clinic, by telephone consultation or through telemedicine.
The first patient was enrolled on June 29th, 2017 and the last patient on July 26th, 2021.

Outcomes

The primary outcome was to determine safety and feasibility. Patients were closely monitored for adverse events until 100 days after the last dose of immunotherapy and were graded according to the Common Terminology Criteria for Adverse Events (CTCTAE) version 4.03. Safety was evaluated on the basis of adverse events and serious adverse events. Feasibility was evaluated by treatment-realted adverse events leading to surgical delay past the predefined 6 weeks after study enrollment.

Secondary and translational endpoints included efficacy evaluated by histopathologic response and clinical outcomes as well as assessment of associations between respons and genomic, transcriptomic and imaging mass cytometry findings of the tumor microenvironment.

Plants

Seed stocks	Not applicable
Novel plant genotypes	Not applicable
Authentication	Not applicable
